# Another Look at Pyrroloiminoquinone Alkaloids—Perspectives on Their Therapeutic Potential from Known Structures and Semisynthetic Analogues

**DOI:** 10.3390/md15040098

**Published:** 2017-03-29

**Authors:** Sheng Lin, Erin P. McCauley, Nicholas Lorig-Roach, Karen Tenney, Cassandra N. Naphen, Ai-Mei Yang, Tyler A. Johnson, Thalia Hernadez, Ramandeep Rattan, Frederick A. Valeriote, Phillip Crews

**Affiliations:** 1 Department of Chemistry and Biochemistry, University of California, Santa Cruz, CA 95064, USA; linsheng2014cn@gmail.com (S.L.); emccaule@ucsc.edu (E.P.M.); nlorigro@ucsc.edu (N.L.-R.); ktenney@ucsc.edu (K.T.); cnaphen@gmail.com (C.N.N.); aimeiyang@163.com (A.-M.Y.); tyler.johnson@dominican.edu (T.A.J.); taj_@chemistry.ucsc.edu (T.H.); 2 State Key Laboratory of Bioactive Substance and Function of Natural Medicines, Institute of Materia Medica, Chinese Academy of Medical Sciences and Peking Union Medical College, Beijing 100050, China; 3 School of Life Science and Engineering, Lanzhou University of Technology, Lanzhou 730050, China; 4 Department of Internal Medicine, Division of Hematology and Oncology, Henry Ford Hospital, Detroit, MI 48202, USA; rrattan1@hfhs.org (R.R.); FVALERI1@hfhs.org (F.A.V.)

**Keywords:** makaluvamine, *Zyzzya fuliginosa*, marine sponge, MS-MS fragmentation profiling, PANC-1 and OVCAR-5 cytotoxicity

## Abstract

This study began with the goal of identifying constituents from *Zyzzya fuliginosa* extracts that showed selectivity in our primary cytotoxicity screen against the PANC-1 tumor cell line. During the course of this project, which focused on six *Z*. *fuliginosa* samples collected from various regions of the Indo-Pacific, known compounds were obtained consisting of nine makaluvamine and three damirone analogues. Four new acetylated derivatives were also prepared. High-accuracy electrospray ionization mass spectrometry (HAESI-MS) *m*/*z* ions produced through MS^2^ runs were obtained and interpreted to provide a rapid way for dereplicating isomers containing a pyrrolo[4,3,2-*de*]quinoline core. In vitro human pancreas/duct epithelioid carcinoma (PANC-1) cell line IC_50_ data was obtained for 16 compounds and two therapeutic standards. These results along with data gleaned from the literature provided useful structure activity relationship conclusions. Three structural motifs proved to be important in maximizing potency against PANC-1: (i) conjugation within the core of the ABC-ring; (ii) the presence of a positive charge in the C-ring; and (iii) inclusion of a 4-ethyl phenol or 4-ethyl phenol acetate substituent off the B-ring. Two compounds, makaluvamine J (**9**) and 15-*O*-acetyl makaluvamine J (**15**), contained all three of these frameworks and exhibited the best potency with IC_50_ values of 54 nM and 81 nM, respectively. These two most potent analogs were then tested against the OVCAR-5 cell line and the presence of the acetyl group increased the potency 14-fold from that of **9** whose IC_50_ = 120 nM vs. that of **15** having IC_50_ = 8.6 nM.

## 1. Introduction

A priority in our study of marine-derived alkaloids has been to explore bioactive products isolable from both sponges and microorganisms. Exploring such molecules offers the prospect of rapidly assembling multi-compound libraries for comprehensive bioactivity and biosynthetic investigations. High profile, contemporary examples of such campaigns include studies from our lab and from others involving bioactive entities, such as: (i) bengamides from sponges (*Jaspis coriacea*) [1] and Gram-negative bacteria (*Myxococcus virescens*) [2,3]; (ii) manzamines from sponges (*Acanthostrongylophora* sp.) [4] and Gram-positive bacteria (*Micromonospora* sp. strain M42) [5]; and (iii) onnamide A from sponges (*Theonella swinhoei*, yellow chemotype) [6] and the sponge-associated *Candidatus entotheonella* [7]. Motivated by the potential to expand on this circumstance we began a deep examination of *Zyzzya* sponge metabolites to further assess their bioactivity and to design future experiments that probe the potential of this sponge as a source of chemically prolific bacteria.

**Figure 1:**
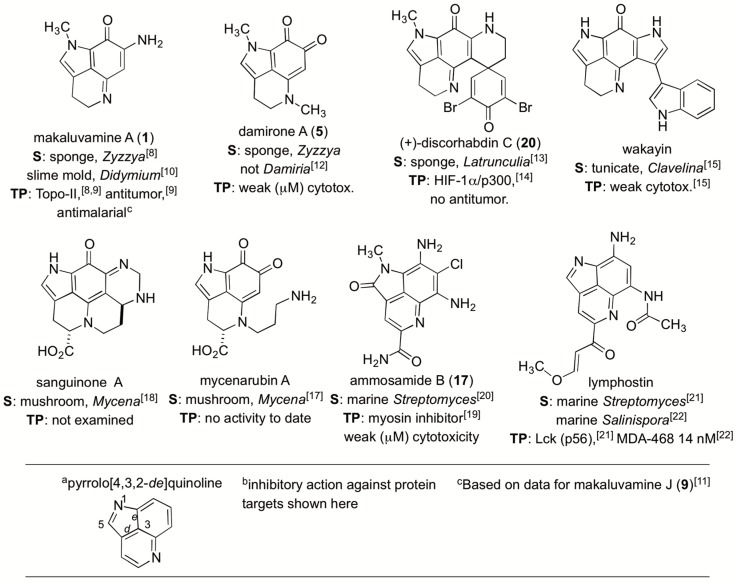
A summary of similar natural products containing the C_10_N_2_ pyrrolo[4,3,2-*de*]quinoline core along with their sources (**S**) and therapeutic potential (**TP**).

During expeditions to multiple Indo-Pacific sites, our attention was repeatedly drawn to *Zyzzya fuliginosa*, a ubiquitous, black burrowing sponge that exudes black mucus on collection. *Z. fuliginosa* reliably affords compounds possessing the pyrrolo[4,3,2-*de*]quinoline core shown in Figure 1. Two years ago several specimens in our repository were flagged for a priority study when a methanol extract fraction of *Z*. *fuliginosa* (coll. No. 93132 DMM) obtained from Papua New Guinea exhibited potent and selective in vitro cytotoxic activity against the human pancreas/duct epithelioid carcinoma (PANC-1) cell line. Solvent partitioning of the crude extract concentrated the activity into a highly pigmented methanol fraction whose ^1^H-NMR spectrum displayed low-field singlets at δ 7.3/δ 6.3 and upfield A_2_X_2_ multiplets δ 3.8/δ 2.9 characteristic of makaluvamines [8,9,10,11,12,13,14,15]. We recognized that: (i) the initial lead bioactive compounds of this family (Figure 1) were reported decades ago; makaluvamine A (**1**) in 1993 [8,9], and discorhabdins A-C in 1986 [13]; and (ii) no such analogs have been evaluated in human clinical trials. The paucity of pancreatic cancer selective agents useful as either therapeutic leads or clinical agents motivated our exploratory work to isolate *Z*. *fuliginosa* constituents and then prepare new semi-synthetic analogs of the most active makaluvamines. The major goal was to identify one or more potent (low nanomolar active) compounds active against pancreatic tumor cell lines.

At an early stage in the project we sought understanding on the scope of natural “makaluvamine-type” scaffolds known from the marine and terrestrial environment. Highlights of important patterns shown in Figure 1 are organized around compounds containing the C_10_N_2_ pyrrolo[4,3,2-*de*]quinoline. This framework is now considered to be created by ribosomally synthesized and post-translationally modified peptide (RiPP) pathways delivering a C-terminal tryptophan building block [16]. The compilation in Figure 1 also abstracts therapeutic assessment outcomes for eight different lead structures [8,9,10,11,12,13,14,15,17,18,19,20,21,22]. Two sponge genera, *Zyzzya* and *Latrunculia*, [23] are abundant sources of pyrrolo[4,3,2-*de*]quinolines which now number close to 100 structures. To date only one compound, wakayin [15], has been isolated from a tunicate. Additionally, five of the molecules in Figure 1 are products of biosynthetic machinery present in microorganisms. Most notably, makaluvamine A (**1**) repeatedly isolated from *Zyzzya* [23] has also been reported from a terrestrial slime mold [10]. Mushrooms produce further functionalized core skeletons represented by sanguinone A [18] and mycenarubin A [17]. Marine-derived Gram-positive microorganisms are the source of: (a) ammosamides [19,20] possessing halogen substituents; and (b) lymphostins [21,22] containing additional residues created by PKS biosynthesis. While several of the compounds in Figure 1 inhibit druggable cancer targets few of them shown here or elsewhere exhibit single or double digit nanomolar in vitro potency.

Many of the natural pyrrolo[4,3,2-*de*]quinolines (Figure 1) and the more than 80 designed synthetic analogues prepared to date [23] show interesting therapeutic potential. Inexplicably, none of these compounds are under current pre-clinical investigations even though great promise was expressed in 2005 by the National Cancer Institute—U. Utah collaborative study [9] which contained the following passages: “The makaluvamines promote topoisomerase II DNA cleavage in vitro.” and “There may be yet other mechanisms of action involved in the antitumor activity of the pyrroloiminoquinones…makaluvamine H and I may warrant further in vivo evaluations in a broader panel of solid tumors.” Overall, the lack of recent forward progress in the pre-clinical investigation of these compounds is dismaying; especially in light of evidence showing that their anti-tumor activity may be via mechanisms beyond topoisomerase II inhibition [24].

Motivated by the positive PANC-1 selective data for the *Z*. *fuliginosa* (coll. No. 93132) extract, we searched for additional literature data. These surveys revealed four pyrrolo[4,3,2-*de*]quinolines: isobatzelline A, C, D, and secobatzelline A from a Caribbean *Batzella* sp. of sponge [23], and one synthetic analog, FBA-TPQ (7-(4-fluorobenzylamino)-1,3,4,8-tetrahydropyrrolo[4,3,2-*de*]quinolin-8(1H)-one) [25] which displayed moderate cytotoxicity against PANC-1 cells. Thus far, there have been no comprehensive studies on the makaluvamines evaluating their cytotoxicity against PANC-1 cells; however the pyrrolo[4,3,2-*de*]quinoline, isobatzellin C, a poor topoisomerase II inhibitor exhibited an IC_50_ of 10 µM against PANC-1 [26]. We now describe the isolation, structure modification, and bioactivity assessment of nine makaluvamines, three damirones, and four new semi-synthetic acetate esters. Once the structures of this collection were established we obtained data against PANC-1 and OVCAR-5 cell lines to establish a structure in vitro cytotoxicity activity relationship for this class of molecules. We discuss below the rationale and possible next steps for the further development of these compounds as therapeutic leads.

## 2. Results

The experimental design employed in this study involved the assembly of a small compound library consisting of natural and semi-synthetic pyrrolo[4,3,2-*de*]quinolines for additional study of their potential as leads for marine derived anti-cancer drugs. For decades the major mechanism of action for this class has been repeatedly described as involving topoisomerase II inhibition, which has dampened enthusiasm for their further study. The new twist here involved assembling a library of compounds for detailed evaluation as selective cytotoxins that could involve different molecular targets. Even though no new natural analogues were being pursued in our study it appeared productive to further explore bioassay properties of the pyrrolo[4,3,2-*de*]quinolines by further functionalizing the amino side chain with various substituents. Such structures are analogous to the family of TPQ synthetic analogs extensively explored by Velu and Zhang [27] whose properties relative to those of the makaluvamines will be further discussed below.

### 2.1. The Isolation Campaign

We began in this study with a focused re-investigation of *Zyzzya* sponges in order to isolate and re-screen makaluvamines C, H, and I described in 2005 [9] as “most potent and differential.” Of further relevance to this plan is that two of these compounds exhibited promising in vivo T/C% (tumor volume in treated/tumor volume in untreated) in KB mouse model xenografts (H = 38%, I = 34%) [9]. Since that publication none of these compounds or other natural congeners have been further investigated. Consequently, we formulated a campaign to obtain this trio of compounds. A second goal was to explore a University of California Santa Cruz (UCSC) repository sample (*Z. fuliginosa*, coll. No. 93132) having semi-pure fractions with PANC-1 selective cytotoxicity. Its methanol-soluble fraction (coded DMM) exhibited an inhibitory zone differential of 8 cm between PANC-1 cells and “normal” CFU-GM cells at 180 µg per disk. NMR and LCMS evaluation of the crude extracts implied that four compounds could be obtained including makaluvamines C (**2**), D (**7**), J (**9**), and damirone A (**5**). While a multi-milligram sample of makaluvamine H (**4**) was available from the UCSC repository, there were no samples of makaluvamine I available.

**Figure 2:**
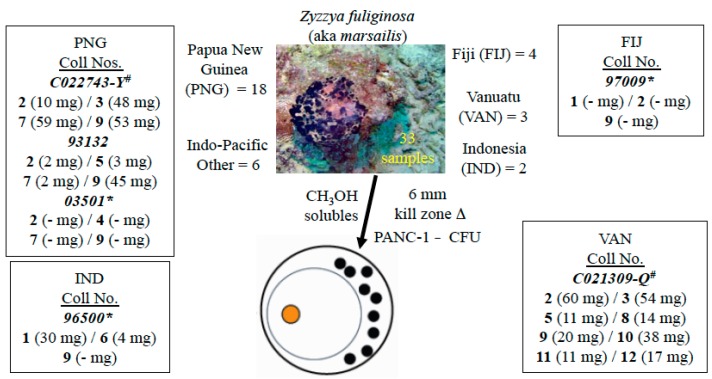
*Zyzzya fuliginosa* sponges for the isolation campaign of their cytotoxic pyrrolo[4,3,2-*de*]quinoline constituents (**1**–**12**). Six of the 33 repository samples available were extensively investigated using * UCSC and ^#^ NCI-DTP material.

Isolation work on the sample (collection number 93132) described above provided 10 mg of makaluvamine C (**9**). We were convinced that other compounds could be efficiently obtained once a repertoire of makaluvamine-containing *Z. fuliginosa* sponges was assembled. This was successfully achieved and summarized in Figure 2 along with a representative underwater photograph of *Z. fuliginosa*. There were 33 samples in total; the UCSC repository was the source of 15 of these and 18 were provided from the National Cancer Institute-Developmental Therapeutics Program (NCI-DTP) branch. Overall, these sponges were collected from more than four major Indo-Pacific zones and six of these samples, chosen to reflect diverse Indo-Pacific collection locales, were selected for further work-up.

**Figure 3:**
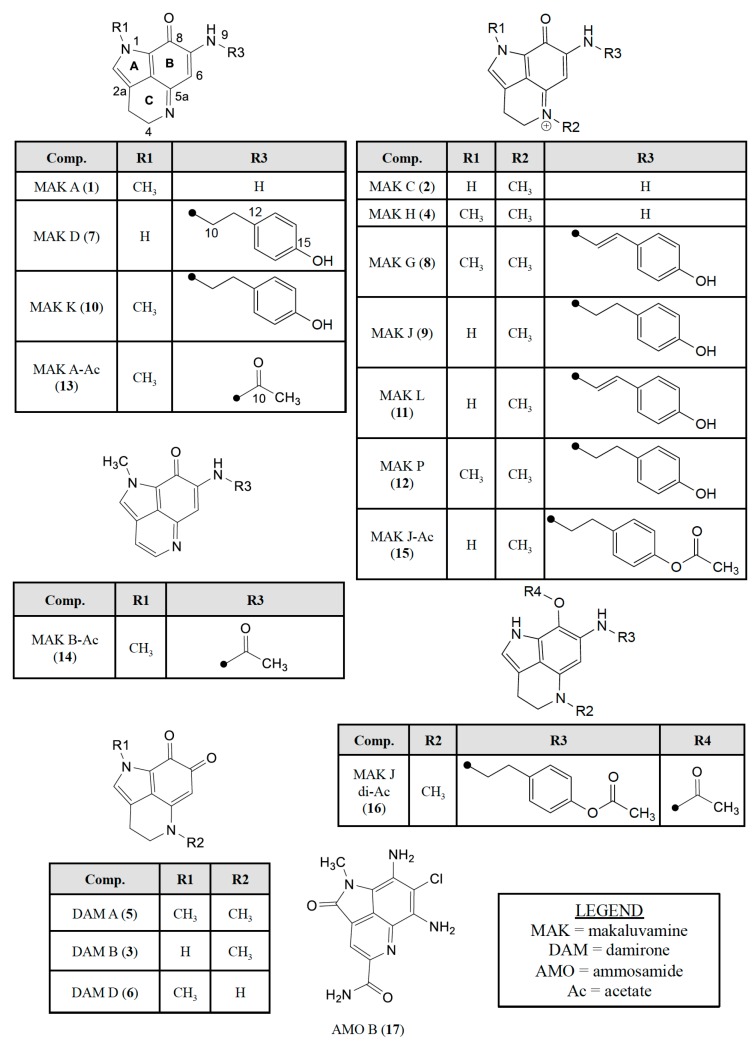
Pyrrolo[4,3,2-*de*]quinoline containing natural products screened against the PANC-1 cell line in this study consisting of: (i) makaluvamines A (**1**), C (**2**), H (**4**), D (**7**), G (**8**), J-L (**9**–**11**), P (**12**); (ii) damirones A (**5**), B (**3**), D (**6**); (iii) semisynthetic makaluvamine acetates 9-*N*-acetyl makaluvamine A (**13**), 9-*N*-acetyl makaluvamine B (**14**), 15-*O*-acetyl makaluvamine J (**15**), 8,15-*O*-diacetyl-8-hydroxy-5a,7,8a-trien-makaluvamine J (**16**); and (iv) ammosamide B (**17**).

The work flow on the six samples shown in Figure 2 proceeded uneventfully and subsequently provided known compounds **1**–**12** (Figure 3) all containing the pyrrolo[4,3,2-*de*]quinoline core (Figure 1). Details of the isolation results are shown in Supplementary Materials Schemes S1–S5. Highlights of the dereplication steps including ^1^H-NMR and MS^2^ data will be discussed later. The sustained work on these samples provided large multi-milligram quantities of four compounds: makaluvamine C (**2**) = 72 mg, damirone B (**3**) = 102 mg, makaluvamine D (**7**) = 61 mg, and makaluvamine J (**9**) = 118 mg. Smaller amounts of seven compounds were also obtained: makaluvamine A (**1**) = 30 mg, damirone A (**5**) = 14 mg, damirone D (**6**) = 4 mg, makaluvamine G (**8**) = 14 mg, makaluvamine K (**10**) = 38 mg, makaluvamine L (**11**) = 11 mg, and makaluvamine P (**12**) = 17 mg.

### 2.2. Dereplicating Pyrrolo[4,3,2-de]quinolines Using MS^2^ Patterns

**Scheme 1:**
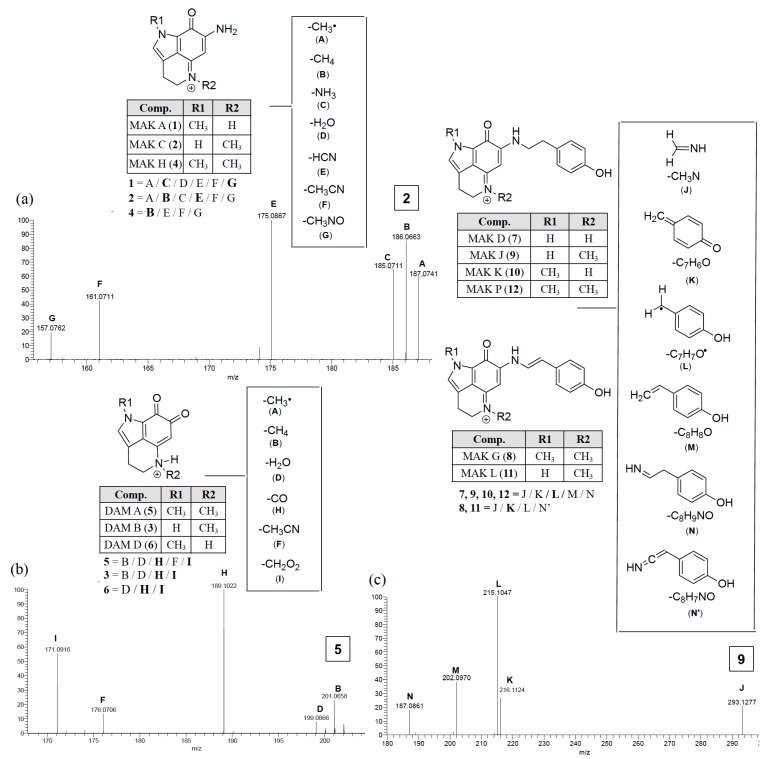
Makaluvamine and damirone MS^2^ fragmentation ions diagnostic for *N*-alkyl substitutions for the pyrolo[4,3,2-*de*]quinoline core family. Annotated peaks, especially intense *m*/*z* ions in bold, represent a signature fingerprint. The panels (**a**, **c**) illustrate fragmentations for the iminocyclohexadienones (examples MAK C (**2**) and MAK J (**9**)) and panel (**b**) shows fragmentation for the animocyclohexadienediones (example DAM A (**5**)).

Employing only ^1^H-NMR data to engage in rapid dereplication of a pyrrolo[4,3,2-*de*]quinolines can be challenging. The major problem is that the resonances that might be used to characterize the core skeleton are not rich with information. Signals for five to six distinct protons assignable to this core (Table S1) are either singlet or triplet resonances, which does not allow making interconnections between isolated spin systems. Consequently, we sought data from high accuracy electrospray ionization mass spectrometry (HAESI-MS) *m*/*z* ions produced through MS^2^ runs. Aside from a lone MS-MS study on synthetic makaluvamine analogs FBA-TPQ/PEA-TPQ [25] no such data can be found in the literature. Consequently, as each compound was purified and analyzed by ^1^H-NMR the dataset was expanded to include MS^2^ analysis. Typical results are shown in Scheme 1 and diagnostic patterns could be identified as discussed further below.

Distinct MS^2^ fingerprints (Scheme 1) resulted from the loss of specific functional groups from the parent ions depending on the class of compound. The functional groups were coded with specific letters (**A**–**N**) and the MS^2^ peaks that correspond to the parent mass minus that functional group are labeled with that letter code. There were four distinct MS^2^ fingerprints that arose from **1** to **12**. The first was observed from the makaluvamines that did not contain a *N*-aryl phenol substituent off the B-ring, makaluvamines A (**1**), C (**2**), and H (**4**) (Scheme 1a). These compounds generated MS^2^ fragments that corresponded to the loss of the following functional groups: CH_3_^•^ (**A**), CH_4_ (**B**), NH_3_ (**C**), H_2_O (**D**), HCN (**E**), CH_3_CN (**F**), and CH_3_NO (**G**). The most intense of which were the loss of **C** from **1**, **E** from **2**, and **B** from **4**. The second fingerprint observed was from damirones B (**3**), A (**5**), and D (**6**) (Scheme 1b), these compounds generated fragments that resulted from the loss of similar groups as the makaluvamines A (**1**), C (**2**), and H (**4**), such as **A**, **B**, **D**, and **F**. However, the most intense fragments resulted from the loss of CO (**H**) and CH_2_O_2_ (**I**), two fragmentation ions that did not arise from any of the makaluvamine analogs and were diagnostic of the damirone ring core. The third MS^2^ fingerprint observed was from the makaluvamines with a 4-ethyl phenol substituent off the B-ring, D (**7**), J (**9**), K (**10**), and P (**12**) (Scheme 1c). These compounds had identical MS^2^ fingerprints that arose from the loss of CH_3_N (**J**), C_7_C_6_O (**K**), C_7_H_7_O^•^ (**L**), C_8_H_8_O (**M**), and C_8_H_9_NO (**N**), with the most intense ion resulting from the loss of L. The last MS^2^ fingerprint observed in this study arose from the makaluvamines with a 4-ethenyl phenol substituent off the B-ring, G (**8**) and L (**11**). The MS^2^ fingerprints from **8** and **11** arose from the loss of **E**, **K**, **L**, and an unsaturated form of **N** (**N’**), with the most intense ion resulting from the loss of **K**.

Once these distinct MS^2^ fingerprints were understood it became possible to engage in MS^2^-driven dereplication and quickly identify **1**–**12** in *Z. fuliginosa* extracts. For example, **1** and **2** have the same *m*/*z* but different fragmentation ion ratios, in that the major fragment from **1** is **C** and **2** is **E**, the same is true of **3** and **6**, where **3** generates a **B** fragment and **6** does not. Additionally, the loss of **H** and **I** from the damirones (**3**, **5** and **6**) made these readily identifiable from the makaluvamines without substituents off the B-ring (**1**, **2**, and **4**) compounds that are otherwise similar in mass and ^1^H-NMR signals. All MS^2^ spectra and predicted fragmentation structures are shown in the Supplementary Materials (Figures S17 and S31) along with a summary of the makaluvamine and damirone analogs present in some of the *Z. fuliginosa* extracts in the UCSC repository (Table S2).

### 2.3. Semi-Synthesis of Acetylated Makaluvamines and Their Identification Using ^1^H-NMR and MS^2^ Data

**Scheme 2:**
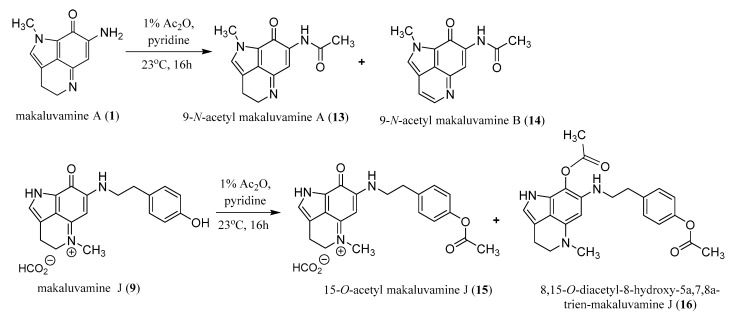
Outcomes of the acetylation of makaluvamines A (**1**) and J (**9**).

To determine if the makaluvamines could be candidates for further clinical development as antibody drug conjugates (ADC) we wanted to ensure that the addition of a functional group to the amide or *N*-alkyl phenol would not result in the loss of cytotoxic activity. Therefore, acetylated derivatives were prepared from makaluvamine A (**1**) and J (**9**) (Scheme 2). The acetylation of **1** (Scheme S6) with acetic anhydride in pyridine resulted in the production of 9-*N*-acetyl makaluvamine A (**13**). The structure of **13** was confirmed by HAESI-MS and MS^2^ data in addition to comparison of its ^1^H-NMR spectrum to that of **1**. The HAESI-MS supported a molecular formula of C_13_H_13_N_3_O_2_, and the MS^2^ fingerprint (Figure S29) contained a fragment corresponding to the loss of the acetyl group to give **1**. Comparison of the ^1^H-NMR spectra (Table 1; Figures S1 and S13) of **1** and **13** showed very little variation with the exception of an additional singlet at δ 2.34 corresponding to the acetate methyl and the downfield shift of the resonance for the proton at position 6 from δ 5.61 to δ 6.31. In addition to **13** the aromatized 9-*N*-acetyl makaluvamine B (**14**) were also obtained from the acetylation of **1**. The evidence for aromatization in **14** is a marked downfield shift of the resonances for protons 3 and 4 from triplets at δ 2.70 and δ 3.95 to doublets at δ 7.75 and δ 8.43, respectively (Table 1; Figures S1 and S14). Additionally, the HAESI-MS *m*/*z* data supported a molecular formula of C_13_H_11_N_3_O_2_ and the MS^2^ spectrum revealed the loss of the acetyl group resulting in a fragment with the correct *m*/*z* for makaluvamine B.

Attempts to acetylate makaluvamine C (**2**) proved unsuccessful, perhaps due to the presence of the methyl at position *N*-5 hindering the aromatization of the C-ring. However, the presence of the *N*-5 methyl did not hinder the acetylation of the B-ring carbonyl or 4-ethyl phenol substituent as was seen by the successful acetylation of **9** (Scheme S7), which yielded 15-*O*-acetyl makaluvamine J (**15**) and 8,15-*O*-diacetyl-8-hydroxy-5a,7,8a-trien-makaluvamine J (**16**). The structure of **15** was confirmed by HAESI-MS and MS^2^ fingerprint (Figure S31). The observed *m*/*z* supports a molecular formula of C_21_H_22_N_3_O_3_ for the cation, and the MS^2^ fragments observed corresponding to the loss of an acetyl plus **J**, **L**, **M**, and **N**. This fragmentation pattern is similar to the MS^2^ fingerprint of **9** (Scheme 1c), which could only be observed if the acetyl group was on the 4-ethyl phenol substituent. Furthermore, there was little variation between the ^1^H-NMR spectra of **9** and **15** aside from the appearance of the methyl acetate singlet at δ 2.25 and the downfield shift for the resonance of protons at position 14 from δ 6.69 to δ 7.29 (Table 1; Figures S9 and S15). The HAESI-MS of **16** confirmed a molecular formula of C_23_H_25_N_3_O_4_ and the MS^2^ fingerprint yielded fragments consistent with those predicted for **9** with acetyl groups on both the B-ring carbonyl and 4-ethyl phenol (Figure S32). This was further confirmed by comparison of the ^1^H-NMR of **16** to **9** where the spectrum of **16** shows the presence of two methyl acetate singlets at δ 2.09 and δ 2.27 and a downfield shift of the resonance for the proton at position 6 from δ 5.59 to δ 6.17 (Table 1; Figures S9 and S16). While the presence of the *N*-5 methyl hindered the acetylation of **2**, its presence along with the absence of the *N*-1 methyl appears to be critical in the acetylation of makaluvamines containing a 4-ethyl phenol substituent, as **9** was the only one to be successfully acetylated under the conditions used in this study.

## 3. Discussion

This study was launched in part to explore the following questions—why are the makaluvamine analogues first discovered in 1986 still of interest as potential therapeutics? Previous studies on the pyrrolo[4,3,2-*de*]quinoline framework have identified the natural products makaluvamines C (**2**), H (**4**), I (not shown here) or the synthetic analogue FBA-TPQ (**18**) as being at the top of the list of promising compounds. We created an experimental design to add structural features missing in these natural products, no aryl groups at the *N*-R3 (Figure 3) but present in the *N*-R3 of the synthetic analogs. Two recent reviews in 2009 [28] and 2016 [27] outline the prospects for further promise of the *N*-aryl containing synthetic analogues. Further insights can be now added but this requires the introduction and discussion of the additional biological screening results obtained in our work and gleaned from the literature.

### 3.1. Evaluating Cytotoxicity Data for a Mini-Library of 22 Compounds

The task of IC_50_ assessment for the natural products isolated in this study against the PANC-1 tumor cells line began immediately following the purification and characterization of each pyrrolo[4,3,2-*de*]quinoline obtained (Figure 3). Sufficient amounts for assay screening were obtained for analogues **1**–**12**, and three additional semi-synthetic acetates **14**–**16** (Figure 3) were prepared from makaluvamines A (**1**) and J (**9**). We attempted to obtain other related compounds, based on frameworks shown in Figure 1 from US academic colleagues, but this was largely unsuccessful. However, a sample of ammosamide B (**17**) was obtained from the NCI Molecular Targets Laboratory. Two additional clinically used compounds etoposide and teniposide [29] were also run in the assay. Finally, literature or unpublished data was retrieved for four molecules: FBA-TPQ (**18**) [25], isobatzelline C (**19**) [26], discorhabdin C (**20**) [unpublished NCI-DTP], and gemcitabine [30]. Several insights can be gained through closer examination of these data shown in Table 2.

The IC_50_ values against PANC-1 are spread out and range from 0.04 µM for teniposide a chemotherapy drug acting as a topoisomerase II inhibitor, to 26 µM for ammosamide B (**17**) a cell cycle modulator that targets myosin [19]. For the natural makaluvamines, the IC_50_ values ranged from 0.054 µM for makaluvamine J (**9**) to 6.2 µM for makaluvamine G (**8**), over a 100-fold difference in potency. As for the semi-synthetic analogs from makaluvamine A (**1**) not enough 9-*N*-acetyl makaluvamine A (**13**) was obtained for IC_50_ determination, but the IC_50_ of 9-*N*-acetyl makaluvamine B (**14**) was 91 µM, over a 200-fold reduction in potency vs. that of **1** (IC_50_ = 0.45 µM). There was greater success from one of the semi-synthetic analogs obtained from **9**. The acetate derivative 15-*O*-acetyl makaluvamine J (**15**) retained the double-digit nanomolar potency (IC_50_ = 0.081 µM) observed from **9**, making it a viable candidate for further clinical development as an ADC. 

By contrast, the di-acetate compound 8,15-*O*-diacetyl-8-hydroxy-5a,7,8a-trien-makaluvamine J (**16**), showed triple-digit nanomolar (IC_50_ = 0.59 µM) potency making it not a priority for further studies. Based on the PANC-1 IC_50_ dataset, makaluvamine J (**9**) and 15-*O*-acetyl makaluvamine J (**15**) were selected for additional IC_50_ determination against the human ovarian cancer cell line OVCAR-5. Makaluvamine J (**9**) had an IC_50_ value of 120 nM which was similar in potency to reported values for other pyrrolo[4,3,2-*de*]quinoline containing compounds against OVCAR-3 and OVCAR-5 cell lines (Table 2). Strikingly, the addition of the acetyl group on **15** resulted in a 14-fold increase in potency over **9** with single-digit nanomolar potency of 8.6 nM against OVCAR-5. In summary, compounds **9** and **15** exhibit the most potent IC_50_ values relative to all other natural and synthetic makaluvamine analogs studied to date. Obtaining a deeper understanding of the structural features responsible for the wide range of IC_50_ data shown in Table 2 will be analyzed next.

### 3.2. Assessing Relative PANC-1 Potencies of Pyrrolo[4,3,2-de]quinolines

**Figure 4:**
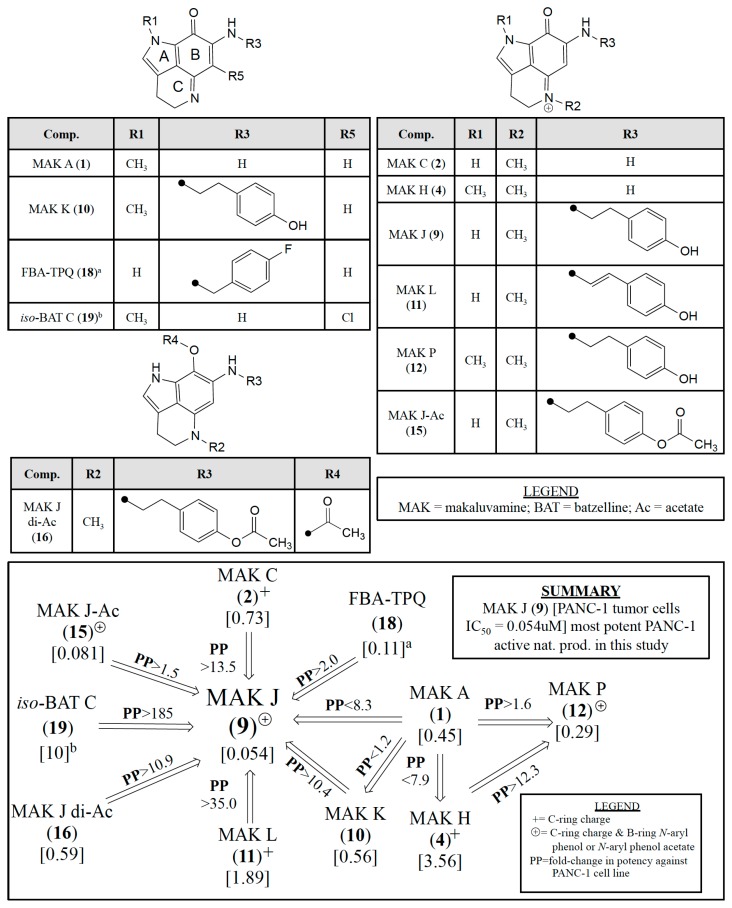
Impact on relative in vitro IC_50_ Potencies [uM] against PANC-1 (coded by **PP** either increasing or (>) decreasing (<)) as a function of: (i) ABC-ring conjugation; (ii) +C-ring charge, and (iii) ⊕C-ring charge with a B-ring *N*-aryl phenol or *N*-aryl phenol acetate substituent for pyrrolo[4,3,2-*de*]quinoline containing molecules. ^a^ data obtained from Zhang et al. [25]; ^b^ data obtained from Guzmán et al. [26].

We believe the quantitative responses of selected compounds against the PANC-1 cell line provide a fresh perspective to plan additional preclinical campaigns based on the makaluvamine framework. The IC_50_ variations for 12 makaluvamine analogs (**1**, **2**, **4**, **7**–**12**, **14**–**16**), one related halogenated compound isobatzelline C (**19**) [26], and the synthetic compound FBA-TPQ (**18**) [25] represent an important learning set. Even though all of the entries of Figure 4 contain an ABC-ring pyrroloiminoquinone core a wide range of PANC-1 IC_50_’s are represented; makaluvamine J (**9**) being the most potent and **19** being relatively inactive. The substituent variations on the tricyclic core shown in Figure 4 are key to review as follows. There are changes at: (i) R1 (CH_3_ or H) on the A-ring; (ii) R2 (CH_3_) on the C-ring (note the + charge at the nitrogen); and (iii) R3 (H, 4-ethyl phenol substituent, 4-ethenyl phenol substituent, 4-ethyl phenol acetate substituent, or 4-fluorobenzyl substituent). Additional variations occur at: (iv) R4 [C(=O)CH_3_/acetate] on the B-ring; and (v) R5 (Cl) on the B-ring of isobatzelline C (**19**) [26]. The inset panel of Figure 4 illustrates the relative impact of these substituent changes on the in vitro IC_50_ **P**otencies [µM] against **P**ANC-1. This impact is coded as **PP** with an increase in potency indicated by “>” and a decrease in potency indicated by “<”. Three relevant SAR structural motifs to note are (i) ABC-ring conjugation; (ii) a C-ring charge coded as “+”; and (iii) a C-ring charge and a B-ring 4-ethyl phenol or 4-ethyl phenol acetate substituent coded as “⊕”.

The boxed panel in Figure 4 highlights that makaluvamine J (**9**) with code “⊕” indicating a C-ring charge and a B-ring 4-ethyl phenol substituent is the most potent against PANC-1, exhibiting an IC_50_ of 0.054 μM. The designed synthetic compound, FBA-TPQ (**18**) [25], does not contain a positive charge on the C-ring but does contain a 4-fluorobenzyl substituent on the B-ring, is ~50% less potent than **9** with an IC_50_ of 0.11 μM. 15-*O*-acetyl makaluvamine J (**15**) which only differs from **9** by the addition of an acetyl group on the R3 substituent is only slightly less potent (**PP** < 1.5) than **9** with an IC_50_ of 0.081 μM. However, the diacetyl makaluvamine J (**16**) which differs from **9** in having an aromatized B-ring and substituents at position R3 and R4, is ~10-fold less potent than **9** as it has different ABC-ring conjugation and no positive charge on the C-ring. Additionally, when comparing **9** and makaluvamine K (**10**) which only differ in their *N*-methyl position (**9** R1 = H, R2 = CH_3_; **10** R1 = CH_3_, R2 = none [no + charge on C-ring]) there is a 10.4-fold decrease in potency, further indicating that the presence of the positive charge on the C-ring is important for potency.

Shown in Figure 4 are the functional group patterns required for the impressive PANC-1 potency of **9** that has R1 = H, R2 = CH_3_, R3 = 4-ethyl phenol. Specifically, less potency was observed for the substitution patterns present in makaluvamine C (**2**) (R1 = H, R2 = CH_3_, R3 = H) and makaluvamine L (**11**) (R1 = H, R2 = CH_3_, R3 = 4-ethenyl phenol). Which resulted in reduced potency compared to **9** with decreases in **PP** of 13.5 and 35.0, respectively. This highlights that the presence of an *N*-aryl substituent at R3 is important for increase potency against PANC-1, but that the flexibility of that substituent is also important as the ethyl phenol analogs (D (**7**), J (**9**), K (**10**), and (P) **12**) show greater potency that the ethenyl phenol analogs (G (**8**), L (**11**)). Comparison of makaluvamines A (**1**) and K (**10**), which only differ at R3 (**1** R3 = H; **10** R3 = 4-ethyl phenol), show minimal difference in potency (**PP** < 1.2). However, when comparing **1** to makaluvamine H (**4**) which only differ at R2 (**1** no R2; **4** R2 = CH_3_) a greater reduction in potency is observed (**PP** < 7.9). Notably, when comparing **4** to makaluvamine P (**12**) which only differ at R3 (**4** R3 = H; **12** R3 = 4-ethyl phenol) there is a markedly greater increase in potency (**PP** > 12.3), further confirming that the presence of an *N*-aryl substituent is important for potency. In summary, the SAR trends of Figure 4 support our selection of **9** and **15** targets for future pre-clinical development.

### 3.3. Secondary Screening

Data summarized above (Table 2, Figure 4) led to the next steps of secondary screening on the prioritized compound, makaluvamine J (**9**). The first follow-up experiments have been completed and utilized a clonogenic assay to assess cell survival through data plotted as a concentration-survival curve [32]. Sponge-derived compounds from our laboratory have shown a favorable profile in such evaluations and include: fascaplysin A [33], fijianolide B [34], japlakinolide [32], and psymberin [35].

**Figure 5:**
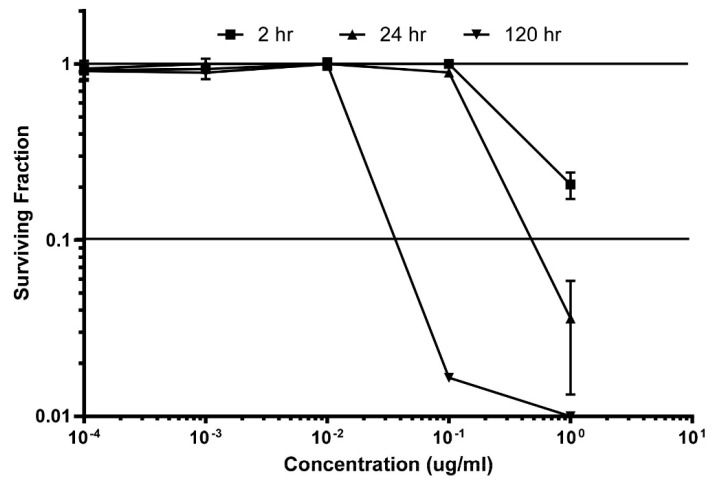
Preclinical evaluation of makaluvamine J (**9**) through clonogenic dose-response evaluation employing HCT-116 cells. Continuous exposure of the cells with **9** at different concentrations during periods of: 2 h, 24 h, or 120 h. Efficacy is indicated when the surviving fraction is less than 0.1.

Accurate determination of the exposures required to achieve a useful in vivo therapeutic effect is the outcome being sought through clonogenic study. Illustrated here are data measuring the cytotoxic effect of makaluvamine J (**9**) at varying concentrations during continuous exposure. Other relevant data to be obtained in the future consists of: (a) repeating the clonogenic runs on makaluvamine acetate J (**15**); (b) obtaining the maximum tolerated dose (MTD) for both; and (c) assessing the pharmacokinetic behavior of these compounds measured in both plasma and tumors, (PANC-1 and OVC-5) at the MTD, which will be tracked through by MS^2^ data. Results from the clonogenic assay of makaluvamine J (**9**) are shown in Figure 5. The key measurement involved determining the required time-concentration profile to obtain a 90% kill (10% survival-S_10_) of tumor cells. Little toxicity to HCT-116 cells was shown at the two hour dosing schedule; and effects observed are as follows: (i) 2 h exposure, _2_S_10_ = 3 µg/mL (extrapolated); (ii) 24 h exposure, _24_S_10_ = 400 ng/mL; and (iii) 120 h exposure, _120_S_10_ = 30 ng/mL. These results are very promising and predict success in the follow up evaluations with PANC-1 and OVCAR-5 tumors. At this juncture it appears that a chronic exposure for five days will be effective and the exact therapeutics regime will depend on the MTD determination data and future pharmacokinetic results. Once these results are in hand we will be able to fully define the necessary drug dose and schedule required to achieve a positive in vivo therapeutic effect [36].

## 4. Material and Methods

### 4.1. General Experimental Procedures

Standard pulse sequences were used for all NMR experiments, which were run on either a Varian UNITY INOVA spectrometer (600 MHz for ^1^H) (Agilent Technologies, Santa Clara, CA, USA) outfitted with a 5 mm triple resonance (HCN) cold probe, a Varian spectrometer (500 MHz for ^1^H) equipped with an inverse detection probe, or a Bruker spectrometer (800 MHz for ^1^H) (Bruker, Billerica, MA, USA) outfitted with a 5 mm triple-resonance (HCN) inverse cold probe. Residual solvent shifts for DMSO-*d*_6_ or CD_3_OD were referenced to δ_H_ 2.50 or δ_H_ 3.31, respectively. Accurate mass measurements for molecular formula determinations were obtained on a Thermo Velos Pro electrospray ionization (ESI) hybrid ion trap-Orbitrap mass spectrometer (Thermo Fisher, Waltham, MA, USA). All HPLC was done in reversed-phase (RP) and utilized HPLC grade CH_3_CN (solvent A) and Milli-Q H_2_O (solvent B), both adjusted to contain 0.1% formic acid (Thermo Fisher). The analytical LC-MS system was composed of Waters HPLC components (i.e., solvent pumps and autosampler) and controlled by Empower software (Waters, Milford, MA, USA). A 150 × 4.60 mm 5 μm Luna C18 column (Phenomenex, Torrance, CA, USA) was utilized, and the system operated at a flow rate of 1 mL/min. The eluent first passed through a photodiode array (Waters) and then was split (95:5) between an evaporative light-scattering detector (ELSD) (SEDEX model 75) (SEDERE, Paris, Ile-de-France, France) and an ESI-time-of-flight mass spectrometer (Applied Biosystems Mariner, Temecula, CA, USA). Column chromatography (CC) was performed using Sephadex LH-20 (40–70 μm; Amersham Pharmacia Biotech AB, Uppsala, Sweden) with MeOH as eluent. The preparative RP-HPLC system was composed of Waters HPLC components (i.e., solvent pumps and gradient controller) and equipped with a 250 × 21 mm 10 μm Synergi MAX-RP column (Phenomenex) and Pharmacia LKB UV-absorbance detector.

### 4.2. Animal Material

Specimens of *Z. fuliginosa* were collected from Papua New Guinea (PNG) (collection number 93132, 1.8 kg wet weight and 03501, 1.2 kg wet weight), Indonesia (96500; 1.2 kg wet weight), and Fiji (97009; 1.9 kg wet weight) using SCUBA at depths between 15 and 30 m by members of the UCSC team, a representative underwater photograph of this organism is shown in Figure 2. Two *Z. fuliginosa* extracts, obtained from the NCI-DTP repository were collected from PNG (C022743-Y) and Vanuatu (C021309-Q).

### 4.3. Extraction and Isolation

*Z. fuliginosa* samples collected by the UCSC laboratories (collection numbers 93132, 96500, 03501, and 97009) were preserved in the field and subsequently extracted using either a standard solvent partition (SSP) or an accelerated solvent extraction (ASE) according to previously described protocols [37]. Extracts obtained from the NCI-DTP collection (collection numbers C021309-Q and C022743-Y) were processed according to NCI protocol [38]. Semi-pure extracted fractions that exhibited selective bioactivity against the PANC-1 cell line were further purified.

Samples coded as 93132 (Scheme S1) and 96500 (Scheme S2) were extracted using the SSP method and the dichloromethane-methanol fraction (coded DMM) contained bioactivity against the PANC-1 cell line. The DMM fractions were further divided into four fractions using preparative HPLC (10:90 CH_3_CN:H_2_O to 100% CH_3_CN, with 0.1% formic acid, 35 min) and labeled H1-H4. From sample 93132, the DMM-H4 fraction was subjected to CC to yield makaluvamine D (**7**) (2 mg) and makaluvamine J (**9**) (45 mg). The DMM-H2 and DMM-H3 fractions were further purified by HPLC (5:95 CH_3_CN: H_2_O to 60:40 CH_3_CN: H_2_O, with 0.1% formic acid) to yield makaluvamine C (**2**) (2 mg) and damirone D (**5**) (3 mg), respectively. From sample 96500, the DMM-H2 and DMM-H3 fractions were further purified by CC to yield makaluvamine A (**1**) (30 mg) and damirone D (**6**) (4 mg), respectively.

Two NCI-DTP extracts coded C021309-Q (Scheme S3) and C022743-Y (Scheme S4) were subjected to CC and divided into seven fractions labeled F1–F7. Workup on the first sample C021309-Q (Scheme S3), began with the F1 and F2 fractions and involved purification by HPLC (5:95 CH_3_CN:H_2_O to 60:40 CH_3_CN:H_2_O, with 0.1% formic acid) to yield makaluvamine P (**12**) (17 mg) and **5** (11 mg), respectively. The F3 fraction was subjected to the same HPLC conditions as fractions F1 and F2 to give 2 (60 mg), damirone B (**3**) (54 mg), and makaluvamine G (**8**) (14 mg). The F3-H4 fraction was further separated by HPLC (20:80 CH_3_CN:H_2_O to 35:65 CH_3_CN:H_2_O, with 0.1% formic acid) to yield **9** (20 mg) and makaluvamine K (**10**) (38 mg). The F4 and F5 fractions were combined and purified by HPLC (5:95 CH_3_CN:H_2_O to 50:50 CH_3_CN:H_2_O, with 0.1% formic acid) to yield makaluvamine L (**11)** (11 mg). Workup on the second sample C022743-Y (Scheme S4) began with the F2 fraction using HPLC (5:95 CH_3_CN:H_2_O to 60:40 CH_3_CN:H_2_O, with 0.1% formic acid) to yield **9** (53 mg). The F3 fraction was also subjected to the same HPLC condition as F2 to give **2** (10 mg), **3** (33 mg), and **7** (21 mg). The F4 fraction was purified by HPLC (5:95 CH_3_CN:H_2_O to 50:50 CH_3_CN:H_2_O with 0.1% formic acid) to yield **3** (15 mg) and **7** (32 mg), and the F5 fraction contained only **7** (6 mg). In total the four *Z. fuliginosa* extracts generated 30 mg of **1**, 72 mg of **2**, 102 mg of **3**, 14 mg of **5**, 4 mg of **6**, 61 mg of **7**, 14 mg of **8**, 118 mg of **9**, 38 mg of **10**, 11 mg of **11**, 17 mg of **12**.

Two additional samples 03501 and 97009 (Scheme S5) were each extracted using the ASE method and the methanol extracts (coded XFM) were determined to have selective bioactivity against the PANC-1 cell line. LCMS analysis was used to identify **2**, makaluvamine H (**4**), **7**, and **9** in the 03501-XFM fraction [37] and **1**, **2**, and **9** in the 97009-XFM fraction. In summary 12 known compounds (**1**–**12**) shown in Figure 3 were obtained and dereplicated by comparing their properties to those in the literature.

### 4.4. Compound Properties

*Makaluvamine A* (**1**): Red-brown solid; ^1^H-NMR (DMSO-*d*_6_, 500 MHz) data, see Table S1 and Figure S1; HAESI-MS *m*/*z* [M + H]^+^ 202.0977 (calcd. for C_11_H_12_N_3_O, 202.0975).

*Makaluvamine C* (**2**): Red-brown solid; ^1^H-NMR (DMSO-*d*_6_, 500 MHz) data, see Table S1 and Figure S2; HAESI-MS *m*/*z* M^+^ 202.0978 (calcd. for C_11_H_12_N_3_O, 202.0975).

*Damirone B* (**3**): Red-brown solid; ^1^H-NMR (DMSO-*d*_6_, 600 MHz) data, see Table S1 and Figure S3; HAESI-MS *m*/*z* [M + H]^+^ 203.0813 (calcd. for C_11_H_11_N_2_O_2_, 203.0810).

*Makaluvamine H* (**4**): Sample obtained from our repository, brown solid; ^1^H-NMR (DMSO-*d*_6_, 500 MHz) data, see Table S1 and Figure S4; HAESI-MS *m*/*z* M^+^ 216.1129 (calcd. for C_12_H_14_N_3_O, 216.1131).

*Damirone A* (**5**): Red-brown solid; ^1^H-NMR (DMSO-*d*_6_, 500 MHz) data, see Table S1 and Figure S5; HAESI-MS *m*/*z* [M + H]^+^ 217.0974 (calcd. for C_12_H_13_N_2_O_2_, 217.0972).

*Damirone D* (**6**): Red-brown solid; ^1^H-NMR (DMSO-*d*_6_, 500 MHz) data, see Table S1 and Figure S6; HAESI-MS *m*/*z* [M + H]^+^ 203.0817 (calcd. for C_11_H_11_N_2_O_2_, 203.0815).

*Makaluvamine D* (**7**): Red-brown solid; ^1^H-NMR (DMSO-*d*_6_, 500 MHz) data, see Table S1 and Figure S7; HAESI-MS *m*/*z* [M + H]^+^ 308.1396 (calcd. for C_18_H_18_N_3_O_2_, 308.1394).

*Makaluvamine G* (**8**): Green solid; ^1^H-NMR (CD_3_OD, 600 MHz) data, see Table S1 and Figure S8; HAESI-MS *m*/*z* M^+^ 334.334.1548 (calcd. for C_20_H_20_N_3_O_2_, 334.1550).

*Makaluvamine J* (**9**): Red-brown solid; ^1^H-NMR (DMSO-*d*_6_, 500 MHz) data, see Table S1 and Figure S9; HAESI-MS *m*/*z* M^+^ 322.1552 (calcd. for C_19_H_20_N_3_O_2_, 322.1550).

*Makaluvamine K* (**10**): Red-brown solid; ^1^H-NMR (DMSO-*d*_6_, 500 MHz) data, see Table S1 and Figure S10; HAESI-MS *m*/*z* [M + H]^+^ 322.1545 (calcd. for C_19_H_20_N_3_O_2_, 322.1550).

*Makaluvamine L* (**11**): Green solid; ^1^H-NMR (CD_3_OD, 500 MHz) data, see Table S1 and Figure S11; HAESI-MS *m*/*z* M^+^ 320.1393 (calcd. for C_19_H_18_N_3_O_2_, 320.1394).

*Makaluvamine P* (**12**): Red-brown solid; ^1^H-NMR (CD_3_OD, 500 MHz) data, see Table S1and Figure S12; HAESI-MS *m*/*z* M^+^ 336.1708 (calcd. for C_20_H_22_N_3_O_2_, 336.1707).

### 4.5. Mass Spectrometry

For each of the makaluvamines and damirones (**1**–**12**) the MS and MS^2^ spectra were obtained using a Thermo Velos Pro-ESI ion trap mass spectrometer using a collision-induced dissociation energy of 35V. Spectra were collected between *m*/*z* of 100 and 500 using XCalibur software (Thermo Fisher). MS^2^ spectra and predicted fragmentation structures are in the Supplementary Materials (Figures S17–S31) and are also shown in Scheme 1.

### 4.6. Acetylation of Makaluvamines A *(**1**)* and J *(**9**)*

Three mg samples of **1** and **9** were dissolved in 500 µL of dried pyridine in a 10 mL scintillation tube then 5 µL of acetic anhydride was added and the reaction solution was kept overnight at room temperature. After the solvent was evaporated under nitrogen, the reaction mixture was purified by HPLC (10:90 CH_3_CN: H_2_O to 60:40 CH_3_CN: H_2_O with 0.1% formic acid) to yield **13** and **14** from **1**, in addition to **15** and **16** from **9**. The overall results from these reactions are shown in Scheme 2 and Supplementary Schemes S6 and S7.

### 4.7. Semi-Synthetic Compound Properties

*9-N-Acetyl makaluvamine A* (**13**): Red-brown solid; ^1^H-NMR (DMSO-*d*_6_, 500 MHz) data, see Table 1 and Figure S13; HAESI-MS *m*/*z* [M + H]^+^ 244.1122 (calcd. for C_13_H_14_N_3_O_2_, 244.1081).

*9-N-Acetyl makaluvamine B* (**14**): Red-brown solid; ^1^H-NMR (CD_3_OD, 600 MHz) data, see Table 1 and Figure S14; HAESI-MS *m*/*z* [M + H]^+^ 242.0927 (calcd. for C_13_H_12_N_3_O_2_, 242.0924).

*15-O-Acetyl makaluvamine J* (**15**): Red-brown solid; ^1^H-NMR (DMSO-*d*_6_, 500 MHz) data, see Table 1 and Figure S15; HAESI-MS *m*/*z* M^+^ 364.1655 (calcd. for C_21_H_22_N_3_O_3_, 364.1656).

*8,15-O-Diacetyl-8-hydroxy-5a,7,8a-trien-makaluvamine J* (**16**): Colorless solid; ^1^H-NMR (CD_3_OD, 800 MHz) data, see Table 1 and Figure S16; HAESI-MS *m*/*z* [M + H]^+^ 408.1880 (calcd. for C_23_H_26_N_3_O_4_, 408.1918).

### 4.8. Cytotoxicity Assays

The soft agar disk diffusion assay, IC_50_ determination, and clonogenic assay were performed as previously described [36].

## 5. Conclusions

In summary, 12 previously known pyrrolo[4,3,2-*de*]quinoline containing makaluvamines and damirones were obtained from *Z. fuliginosa* samples collected from various regions of the Indo-Pacific. HAESI-MS^2^ profiling of these natural products revealed distinct fingerprint patterns that allowed for rapid identification of these compounds in complex sponge extracts. All 12 of these natural products, in addition to three acetylated makaluvamine derivatives, were assayed for cytotoxic activity against the PANC-1 cell line. Data retrieved from this assay identified three structural motifs that were important in maximizing potency against the PANC-1 cell line: (i) conjugation of the core ABC-ring; (ii) a positive charge on the C-ring; and (iii) the presence of a 4-ethyl phenol or 4-ethyl phenol acetate substituent off the B-ring. Makaluvamine J (**9**) and 15-*O*-acetyl makaluvamine J (**15**) containing all three of these motifs and exhibited the greatest nanomolar potency against the PANC-1 and OVCAR-5 cell lines. Previous studies investigating the cytotoxicity of pyrrolo[4,3,2-*de*]quinoline containing compounds for anticancer drug leads have primarily focused on makaluvamines C (**2**), H (**4**), and I, or the synthetic analog FBA-TPQ (**18**). No one to date has looked at makaluvamine J (**9**) as a promising anti-cancer drug lead, therefore we are going forward with makaluvamine J (**9**) and 15-*O*-acetyl makaluvamine J (**15**) for future pre-clinical development and results will be reported in the near future.

## Figures and Tables

**Table 1:** ^1^H-NMR data for makaluvamines A (**1**), J (**9**), and their acetate derivatives (**13**–**16**) prepared as shown in Scheme 2.

Position	A (1) ^a^	A-Ac (13) ^a^	B-Ac (14) ^b^	J (9) ^a^	J-Ac (15) ^a^	J di-Ac (16) ^b^
	δ_H_, mult, (*J*, Hz)					
2	7.27, s	7.17, s	8.15, s	7.31, s	7.13, s	7.32, s
3	2.81, t (7.5)	2.70, t (7.5)	7.75, d (6.0)	2.92, t (7.5)	2.95, brs	3.04, t (7.5)
4	3.77, t (7.5)	3.95, t (7.5)	8.43, d (6.0)	3.89, t (7.5)	3.78, brs	3.93, t (7.5)
6	5.61, s	6.31, s	8.67, s	5.59, s	5.42, s	6.17, s
10				3.60, t (7.0)	3.63, brs	3.76, t (7.5)
11				2.83, t (7.0)	2.85, brs	2.98, t (7.5)
13				7.05, d (8.0)	7.05, d (8.0)	7.03, d (8.0)
14				6.69, d (8.0)	7.29, d (8.0)	7.31, d (8.0)
N1-Me	3.89, s	3.83, s	4.39, s			
N5-Me				3.37, s	3.28, s	3.31, s
Ac		2.34, s	2.26, s		2.25, s	2.27, s 2.09, s

^a 1^H-NMR data measured in DMSO-*d*_6_ at 500 MHz; ^b 1^H-NMR data measured in CD_3_OD at 600 MHz.

**Table 2:** IC_50_ data for pyrrolo[4,3,2-*de*]quinolines and therapeutic standards against ovarian and pancreatic cancer tumor cell lines.

Compound	PANC-1	OVCAR-3/-5
	IC_50_ (μM)	
makaluvamine A (**1**)	0.45	
makaluvamine C (**2**)	0.73	0.24 ^a^/NT
damirone B (**3**)	19	
makaluvamine H (**4**)	3.6	0.96 ^a^/0.10 ^a^
damirone A (**5**)	160	
damirone D (**6**)	3.4	
makaluvamine D (**7**)	0.29	
makaluvamine G (**8**)	6.2	
makaluvamine J (**9**)	0.054	NT/0.12
makaluvamine K (**10**)	0.56	
makaluvamine L (**11**)	1.9	
makaluvamine P (**12**)	0.3	
9-*N*-acetyl makaluvamine B (**14**)	91	
15-*O*-acetyl makaluvamine J (**15**)	0.081	NT/0.0086
8,15-*O*-diacetyl-8-hydroxy-5a,7,8a-trien-makaluvamine J (**16**)	0.59	
ammosamide B (**17**)	26	
FBA-TBQ (**18**)	0.11 ^b^	0.95 ^c^/NT
isobatzelline C (**19**)	10 ^d^	
discorhabdin C (**20**)	NT	0.33 ^e^/2.6 ^e^
etoposide	0.39	
teniposide	0.041	
gemcitabine	7.2 ^f^	

NT = Not Tested; Refs: ^a^ data obtained from Dijoux et al. [9]; ^b^ data obtained from Zhang et al. [25]; ^c^ data obtained from Chen et al. [31]; ^d^ data obtained from Guzmán et al. [26]; ^e^ data obtained from the NCI-DTP 60 Human Tumor Cell Lines Database; ^f^ data obtained from Li et al. [30].

## Supplementary Materials

The following are available online at www.mdpi.com/1660-3397/15/4/98/s1, Schemes S1–S5: Isolation schemes for compounds **1**–**12**, Figures S1–S16: ^1^H-NMR spectra for compounds **1**–**16**, Table S1: ^1^H-NMR data (500/600 MHz) for the makaluvamines (**1**, **2**, **4**, **7**–**12**) and damirones (**3**, **5**, **6**) in DMSO-*d*_6_, Figures S17–S31: MS^2^ spectra for compounds **1**–**16**, Schemes S6 and S7: The acetylation reaction of makaluvamine A (**1**) and J (**9**), Table S2: HAESI-MS^2^ detection of **1**–**12** in *Z. fuliginosa* extracts.

## References

[1] Thale Z., Kinder F.R., Bair K.W., Bontempo J., Czuchta A.M., Versace R.W., Phillips P.E., Sanders M.L., Wattanasin S., Crews P. (2001). Bengamides revisited: New structures and antitumor studies. J. Org. Chem..

[2] Wenzel S.C., Hoffmann H., Zhang J., Debussche L., Haag-Richter S., Kurz M., Nardi F., Lukat P., Kochems I., Tietgen H. (2015). Production of the bengamide class of marine natural products in myxobacteria: Biosynthesis and structure-activity relationships. Angew. Chem. Int. Ed. Engl..

[3] Johnson T.A., Sohn J., Vaske Y.M., White K.N., Cohen T.L., Vervoort H.C., Tenney K., Valeriote F.A., Bjeldanes L.F., Crews P. (2012). Myxobacteria versus sponge-derived alkaloids: The bengamide family identified as potent immune modulating agents by scrutiny of LC-MS/ELSD libraries. Bioorg. Med. Chem..

[4] Furusato A., Kato H., Nehira T., Eguchi K., Kawabata T., Fujiwara Y., Losung F., Mangindaan R.E.P., de Voogd N.J., Takeya M. (2014). Acanthomanzamines A-E with new manzamine frameworks from the marine sponge *Acanthostrongylophora ingens*. Org. Lett..

[5] Waters A.L., Peraud O., Kasanah N., Sims J.W., Kothalawala N., Anderson M.A., Abbas S.H., Rao K.V., Jupally V.R., Kelly M. (2014). An analysis of the sponge *Acanthostrongylophora ingens* microbiome yields an actinomycete that produces the natural product manzamine A. Front. Mar. Sci..

[6] Sakemi S., Ichiba T., Kohmoto S., Saucy G., Higa T. (1988). Isolation and structure elucidation of onnamide A, a new bioactive metabolite of a marine sponge, *Theonella* sp. J. Am. Chem. Soc..

[7] Wilson M.C., Mori T., Rückert C., Uria A.R., Helf M.J., Takada K., Gernert C., Steffens U.A.E., Heycke N., Schmitt S. (2014). An environmental bacterial taxon with a large and distinct metabolic repertoire. Nature.

[8] Radisky D.C., Radisky E.S., Barrows L.R., Copp B.R., Kramer R.A., Ireland C.M. (1993). Novel cytotoxic topoisomerase II inhibiting pyrroloiminoquinones from Fijian sponges of the genus *Zyzzya*. J. Am. Chem. Soc..

[9] Dijoux M.-G., Schnabel P.C., Hallock Y.F., Boswell J.L., Johnson T.R., Wilson J.A., Ireland C.M., van Soest R., Boyd M.R., Barrows L.R. (2005). II Antitumor activity and distribution of pyrroloiminoquinones in the sponge genus *Zyzzya*. Bioorg. Med. Chem..

[10] Ishibashi M., Iwasaki T., Imai S., Sakamoto S., Yamaguchi K., Ito A. (2001). Laboratory culture of the Myxomycetes: Formation of fruiting bodies of *Didymium bahiense* and its plasmodial production of makaluvamine A. J. Nat. Prod..

[11] Davis R.A., Buchanan M.S., Duffy S., Avery V.M., Charman S.A., Charman W.N., White K.L., Shackleford D.M., Edstein M.D., Andrews K.T. (2012). Antimalarial activity of pyrroloiminoquinones from the Australian marine sponge *Zyzzya* sp. J. Med. Chem..

[12] Schmidt E.W., Harper M.K., Faulkner D.J. (1995). Makaluvamines H-M and Damirone C from the Pohnpeian sponge *Zyzzya fuliginosa*. J. Nat. Prod..

[13] Perry N.B., Blunt J.W., McCombs J.D., Munro M.H.G. (1986). Discorhabdin C, a highly cytotoxic pigment from a sponge of the genus *Latrunculia*. J. Org. Chem..

[14] Goey A.K.L., Chau C.H., Sissung T.M., Cook K.M., Venzon D.J., Castro A., Ransom T.R., Henrich C.J., McKee T.C., McMahon J.B. (2016). Screening and biological effects of marine pyrroloiminoquinone alkaloids: Potential inhibitors of the HIF-1α/p300 interaction. J. Nat. Prod..

[15] Copp B.R., Ireland C.M., Barrows L.R. (1991). Wakayin: A novel cytotoxic pyrroloiminoquinone alkaloid from the ascidian *Clavelina* species. J. Org. Chem..

[16] Jordan P.A., Moore B.S. (2016). Biosynthetic pathway connects cryptic ribosomally synthesized posttranslationally modified peptide genes with pyrroloquinoline alkaloids. Cell. Chem. Biol..

[17] Peters S., Spiteller P. (2007). Mycenarubins A and B, red pyrroloquinoline alkaloids from the mushroom *Mycena rosea*. Eur. J. Org. Chem..

[18] Peters S., Spiteller P. (2007). Sanguinones A and B, blue pyrroloquinoline alkaloids from the fruiting bodies of the mushroom *Mycena sanguinolenta*. J. Nat. Prod..

[19] Hughes C.C., MacMillan J.B., Gaudêncio S.P., Fenical W., La Clair J.J. (2009). Ammosamides A and B target myosin. Angew. Chem. Int. Ed. Engl..

[20] Hughes C.C., MacMillan J.B., Gaudêncio S.P., Jensen P.R., Fenical W. (2009). The ammosamides: Structures of cell cycle modulators from a marine-derived *Streptomyces* species. Angew. Chem. Int. Ed. Engl..

[21] Nagata H., Yano H., Sasaki K., Sato S., Nakanishi S., Takahashi I., Tamaoki T. (2002). Inhibition of lymphocyte kinase Lck and phosphatidylinositol 3-kinase by a novel immunosuppressant, lymphostin. Biosci. Biotechnol. Biochem..

[22] Miyanaga A., Janso J.E., McDonald L., He M., Liu H., Barbieri L., Eustáquio A.S., Fielding E.N., Carter G.T., Jensen P.R. (2011). Discovery and assembly-line biosynthesis of the lymphostin pyrroloquinoline alkaloid family of mTOR inhibitors in *Salinispora* bacteria. J. Am. Chem. Soc..

[23] Antunes E.M., Copp B.R., Davies-Coleman M.T., Samaai T. (2005). Pyrroloiminoquinone and related metabolites from marine sponges. Nat. Prod. Rep..

[24] Nag S., Nadkarni D.H., Qin J.-J., Voruganti S., Nguyen T., Xu S., Wang W., Velu S.E., Zhang R. (2012). Anticancer activity and molecular mechanisms of action of makaluvamines and analogues. Mol. Cell. Pharmacol..

[25] Zhang X., Xu H., Zhang X., Voruganti S., Murugesan S., Nadkarni D.H., Velu S.E., Wang M.-H., Wang W., Zhang R. (2012). Preclinical evaluation of anticancer efficacy and pharmacological properties of FBA-TPQ, a novel synthetic makaluvamine analog. Mar. Drugs.

[26] Guzmán E.A., Johnson J.D., Carrier M.K., Meyer C.I., Pitts T.P., Gunasekera S.P., Wright A.E. (2009). Selective cytotoxic activity of the marine-derived batzelline compounds against pancreatic cancer cell lines. Anticancer Drugs.

[27] Wang W., Nijampatnam B., Velu S.E., Zhang R. (2016). Discovery and development of synthetic tricyclic pyrroloquinone (TPQ) alkaloid analogs for human cancer therapy. Front. Chem. Sci. Eng..

[28] Wang W., Rayburn E.R., Velu S.E., Nadkarni D.H., Murugesan S., Zhang R. (2009). In vitro and In vivo anticancer activity of novel synthetic makaluvamine analogues. Clin. Cancer Res..

[29] Nitiss J.L. (2009). Targeting DNA topoisomerase II in cancer chemotherapy. Nat. Rev. Cancer.

[30] Li J.N., Zhu J.X., Melvin W.S., Bekaii-Saab T.S., Chen C.S., Muscarella P. (2006). A structurally optimized celecoxib derivative inhibits human pancreatic cancer cell growth. J. Gastrointest. Surg..

[31] Chen T., Xu T., Guo H., Liu Y., Hu P., Yang X., Li X., Ge S., Velu S.E., Nadkarni D.H. (2011). Experimental therapy of ovarian cancer with synthetic makaluvamine analog: In vitro and in vivo anticancer activity and molecular mechanisms of action. PLoS ONE.

[32] Watts K.R., Morinaka B.I., Arnagata T., Robinson S.J., Tenney K., Bray W.M., Gassner N.C., Lokey R.S., Media J., Valeriote F.A. (2011). Biostructural features of additional jasplakinolide (Jaspamide) analogues. J. Nat. Prod..

[33] Subramanian B., Nakeff A., Tenney K., Crews P., Gunatilaka L., Valeriote F. (2006). A new paradigm for the development of anticancer agents from natural products. J. Exp. Ther. Oncol..

[34] Johnson T.A., Tenney K., Cichewicz R.H., Morinaka B.I., White K.N., Amagata T., Subramanian B., Media J., Mooberry S.L., Valeriote F.A. (2007). Sponge-derived fijianolide polyketide class: Further evaluation of their structural and cytotoxicity properties. J. Med. Chem..

[35] Cichewicz R.H., Valeriote F.A., Crews P. (2004). Psymberin, a potent sponge-derived cytotoxin from *Psammocinia* distantly related to the pederin family. Org. Lett..

[36] Valeriote F.A., Tenney K., Media J., Pietraszkiewicz H., Edelstein M., Johnson T.A., Amagata T., Crews P. (2012). Discovery and development of anticancer agents from marine sponges: Perspectives based on a chemistry-experimental therapeutics collaborative program. J. Exp. Ther. Oncol..

[37] Johnson T.A., Morgan M.V.C., Aratow N.A., Estee S.A., Sashidhara K.V., Loveridge S.T., Segraves N.L., Crews P. (2010). Assessing pressurized liquid extraction for the high-throughput extraction of marine-sponge-derived natural products. J. Nat. Prod..

[38] McCloud T.G. (2010). High throughput extraction of plant, marine and fungal specimens for preservation of biologically active molecules. Molecules.

