# Supplementary material for: Another Look at Pyrroloiminoquinone Alkaloids—Perspectives on Their Therapeutic Potential from Known Structures and Semisynthetic Analogues
Source: Mar Drugs. 2017 Mar 29;15(4):98. doi: 10.3390/md15040098 (PMC5408244; doi:10.3390/md15040098)
Supplement: Supplementary file 1 [file marinedrugs-15-00098-s001.pdf]

``File: Makulav SI R7

Another Look at Pyrroloiminoquinone Alkaloids—New Perspectives on Their Therapeutic Potential from Known Structures and Unique Semisynthetic Analogues

Sheng Lin,[1,3] Erin McCauley,[1] Nicholas Lorig-Roach,[1] Karen Tenney,[1] Cassandra N. Naphen,[1] Ai-Mei Yang,[1,4] Tyler A. Johnson,[1] Thalia Hernadez,[1] Ramandeep Rattan,[2] Frederick A. Valeriote,[2] and Phillip Crews[1*]

[1]*Department of Chemistry and Biochemistry, University of California, Santa Cruz, CA 95064, United States*

[2] *Department of Internal Medicine, Division of Hematology and Oncology, Henry Ford Hospital, Detroit Michigan 48202, United States*

[3] *State Key Laboratory of Bioactive Substance and Function of Natural Medicines, Institute of Materia Medica, Chinese Academy of Medical Sciences and Peking Union Medical College, Beijing 100050, P. R. China.*

[4] *School of Life Science and Engineering，Lanzhou University of Technology, Lanzhou 730050, P. R. China,*

Supplementary Information

[*] To whom correspondence should be addressed.

Tel: (831) 459-2603. Email: pcrews@ucsc.edu

Table of Contents

		Page
Scheme S1	Isolation scheme for compounds **2**, **5**, **7**, and **9** from *Z. fuliginosa* 93132.	S4
Scheme S2	Isolation scheme for compounds **1** and **6** from *Z. fuliginosa* 96500.	S5
Scheme S3	Isolation scheme for compounds **2**, **3**, **5**, and **8**–**12** from *Z. fuliginosa* C021309-Q.	S6
Scheme S4	Isolation scheme for compounds **2**, **3**, **7**, and **9** from *Z. fuliginosa* C022743-Y.	S7
Scheme S5	LC-ELSD chromatograms showing compounds **2**, **4**, **7**, and **9** from *Z. fuliginosa* 03501, and **1**, **2**, and **9** from *Z. fuliginosa* 97009.	S8
Figure S1	The ^{1}H NMR spectrum of makaluvamine A (**1**) in DMSO-d_6 (500 MHz).	S9
Figure S2	The ^{1}H NMR spectrum of makaluvamine C (**2**) in DMSO-d_6 (500 MHz).	S10
Figure S3	The ^{1}H NMR spectrum of damirone B (**3**) in DMSO-d_6 (600 MHz).	S11
Figure S4	The ^{1}H NMR spectrum of makaluvamine H (**4**) in DMSO-d_6 (500 MHz).	S12
Figure S5	The ^{1}H NMR spectrum of damirone A (**5**) in DMSO-d_6 (500 MHz).	S13
Figure S6	The ^{1}H NMR spectrum of damirone D (**6**) in DMSO-d_6 (500 MHz).	S14
Figure S7	The ^{1}H NMR spectrum of makaluvamine D (**7**) in DMSO-d_6 (500 MHz).	S15
Figure S8	The ^{1}H NMR spectrum of makaluvamine G (**8**) in CD_3OD (500 MHz).	S16
Figure S9	The ^{1}H NMR spectrum of makaluvamine J (**9**) in DMSO-d_6 (500 MHz).	S17
Figure S10	The ^{1}H NMR spectrum of makaluvamine K (**10**) in DMSO-d_6 (500 MHz).	S18
Figure S11	The ^{1}H NMR spectrum of makaluvamine L (**11**) in CD_3OD (500 MHz).	S19
Figure S12	The ^{1}H NMR spectrum of makaluvamine P (**12**) in CH_3OD (500 MHz).	S20
Figure S13	The ^{1}H NMR spectrum of 9-*N*-acetyl makaluvamine A (**13**) in DMSO-d_6 (500 MHz).	S21
Figure S14	The ^{1}H NMR spectrum of 9-*N*-acetyl makaluvamine B (**14**) in CD_3OD (600 MHz).	S22
Figure S15	The ^{1}H NMR spectrum of 15-*O*-acetyl makaluvamine J (**15**) in DMSO-d_6 (500 MHz).	S23
Figure S16	The ^{1}H NMR spectrum of 8,15-*O*-diacetyl-8-hydroxy-5a,7,8a-trien-makaluvamine J (**16**) in CD_3OD (800 MHz).	S24
Table S1	^{1}H NMR data (500/600 MHz) for the makaluvamines (**1**, **2**, **4**, **7**–**12**) and damirones (**3, 5, 6**) in DMSO-d_6.	S25
Figure S17	MS^2 spectrum and predicted fragmentation structures of makaluvamine A (**1**).	S26
Figure S18	MS^2 spectrum and predicted fragmentation structures of makaluvamine C (**2**).	S27
Figure S19	MS^2 spectrum and predicted fragmentation structures of damirome B (**3**).	S28
Figure S20	MS^2 spectrum and predicted fragmentation structures of damirome A (**5**).	S29
Figure S21	MS^2 spectrum and predicted fragmentation structures of damirome D (**6**).	S30

Figure S22	MS^2 spectrum and predicted fragmentation structures of makaluvamine D (**7**).	S31
Figure S23	MS^2 spectrum and predicted fragmentation structures of makaluvamine G (**8**).	S32
Figure S24	MS^2 spectrum and predicted fragmentation structures of makaluvamine J (**9**).	S33
Figure S25	MS^2 spectrum and predicted fragmentation structures of makaluvamine K (**10**).	S34
Figure S26	MS^2 spectrum and predicted fragmentation structures of makaluvamine L (**11**).	S35
Figure S27	MS^2 spectrum and predicted fragmentation structures of makaluvamine P (**12**).	S36
Figure S28	MS^2 spectrum and predicted fragmentation structures of 9-*N*-acetyl makaluvamine A (**13**).	S37
Figure S29	MS^2 spectrum and predicted fragmentation structures of 9-*N*-acetyl makaluvamine B (**14**).	S38
Figure S30	MS^2 spectrum and predicted fragmentation structures of 15-*O*-acetyl makaluvamine J (**15**).	S39
Figure S31	MS^2 spectrum and predicted fragmentation structures of 8,15-*O*-diacetyl-8-hydroxy-5a,7,8a-trien-makaluvamine J (**16**).	S40
Table S2	HAESI-MS^2 detection of **1-12** in *Z. fuliginosa* extracts.	S41
Scheme S6	The acetylation reaction of makaluvamine A (**1**).	S42
Scheme S7	The acetylation reaction of makaluvamine J (**9**).	S43

Scheme S1. Isolation scheme for compounds **2**, **5**, **7**, and **9** from *Z. fuliginosa* 93132.

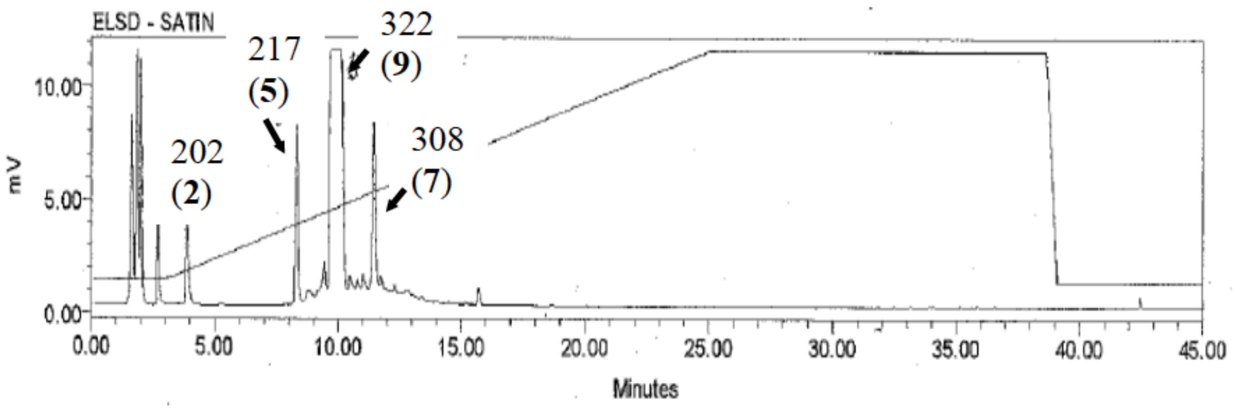

LC-ELSD chromatogram of 93132 DMM fraction

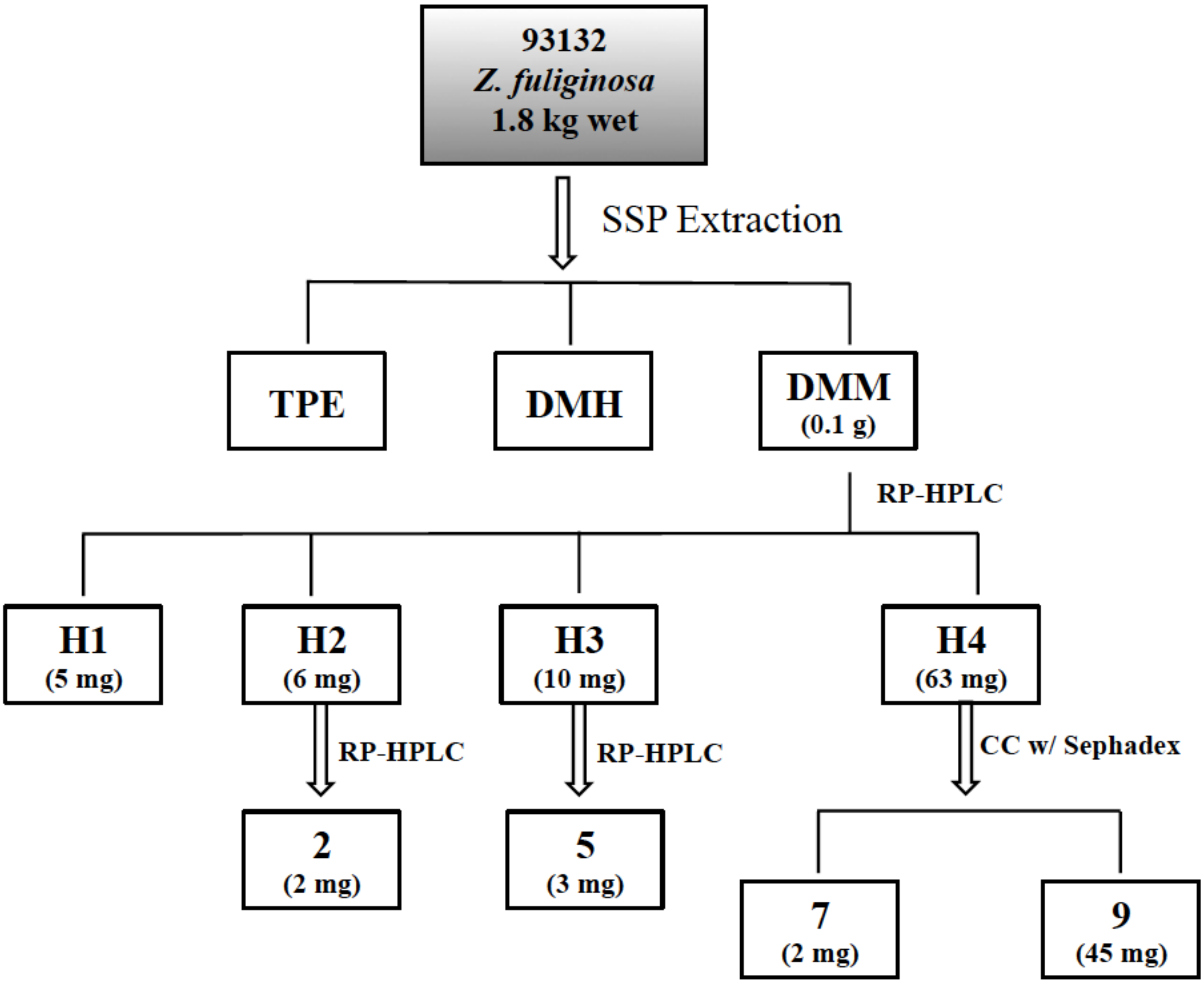

Scheme S2. Isolation scheme for compounds **1** and **6** from *Z. fuliginosa* 96500.

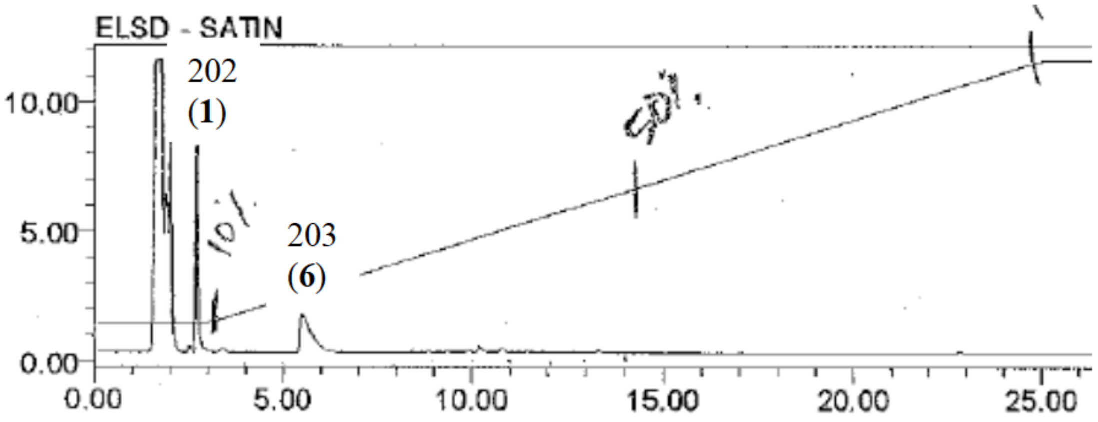

LC-ELSD chromatogram of 96500 DMM fraction

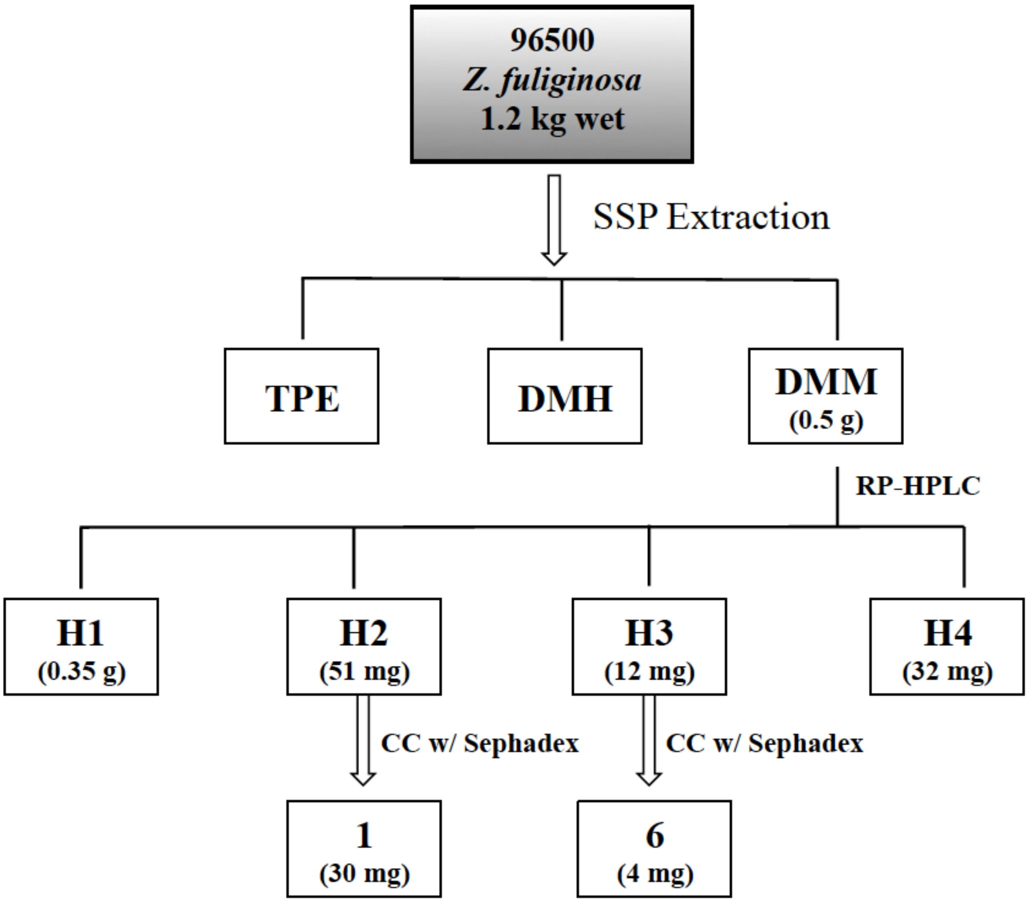

Scheme S3. Isolation scheme for compounds **2**, **3**, **5**, and **8–12** from *Z. fuliginosa* C021309-Q.

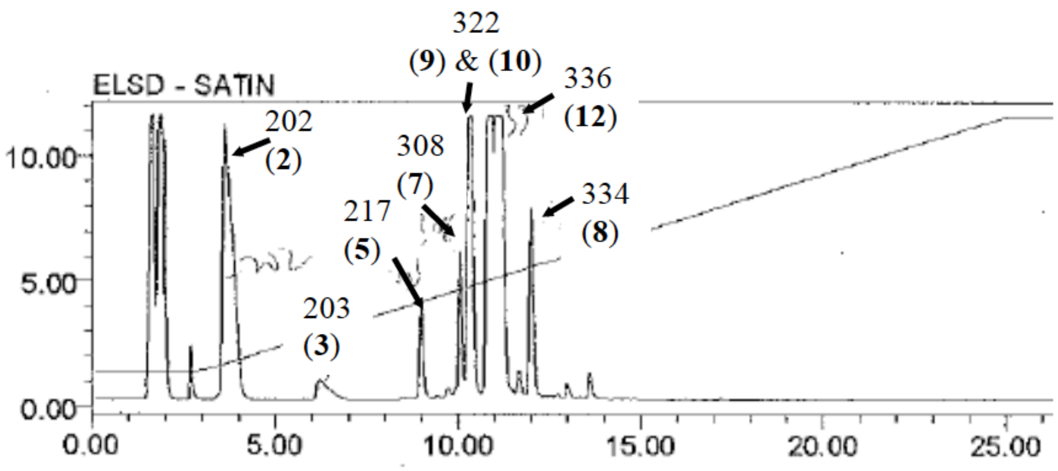

LC-ELSD chromatogram of Q021309-Q extract

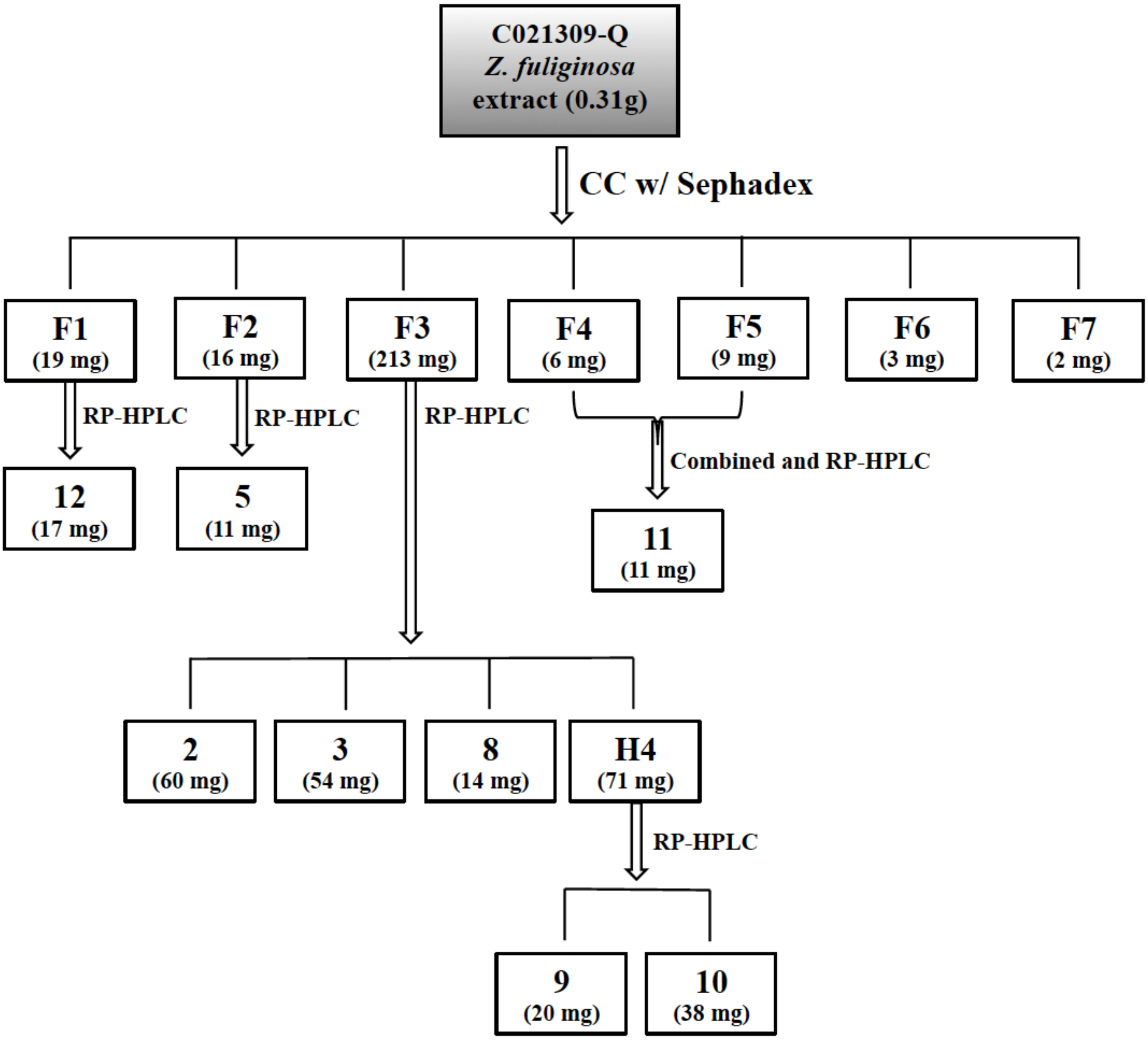

Scheme S4. Isolation scheme for compounds **2**, **3**, **7**, and **9** from *Z. fuliginosa* C022743-Y.

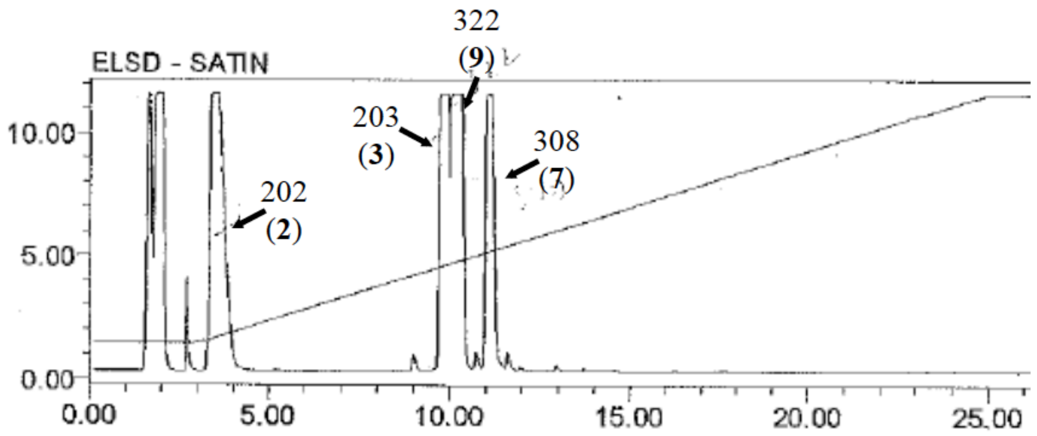

LC-ELSD chromatogram of C022743-Y extract

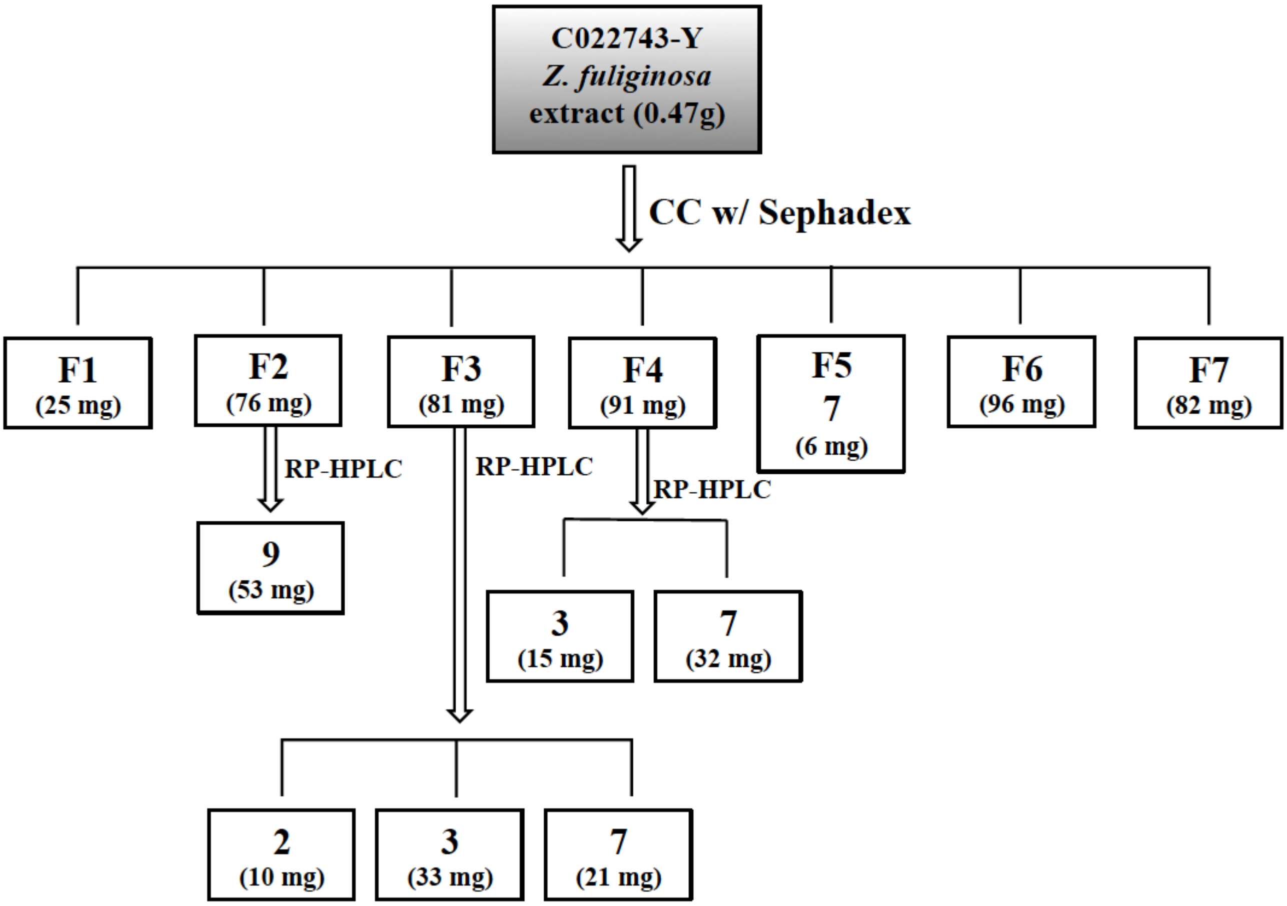

Scheme S5. LC-ELSD chromatograms showing compounds **2**, **4**, **7**, and **9** from *Z. fuliginosa* 03501, and **1**, **2**, and **9** from *Z. fuliginosa* 97009.

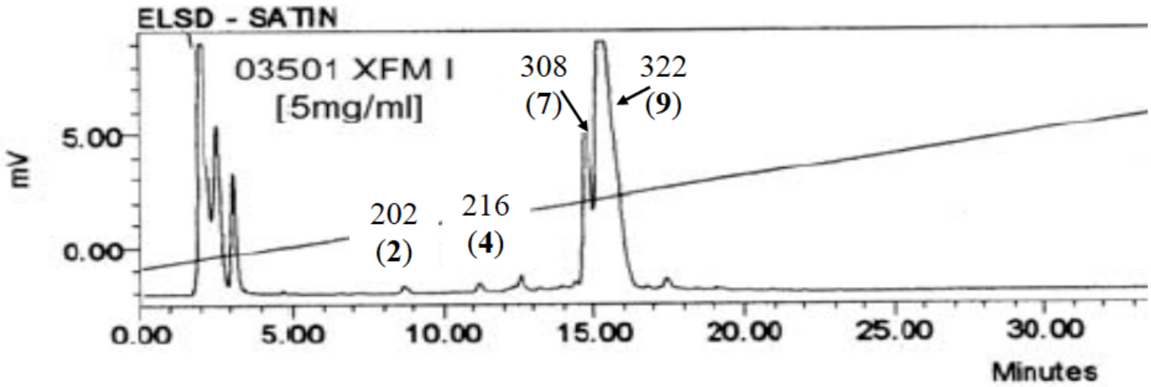

LC-ELSD chromatogram of 03501 XFM extract

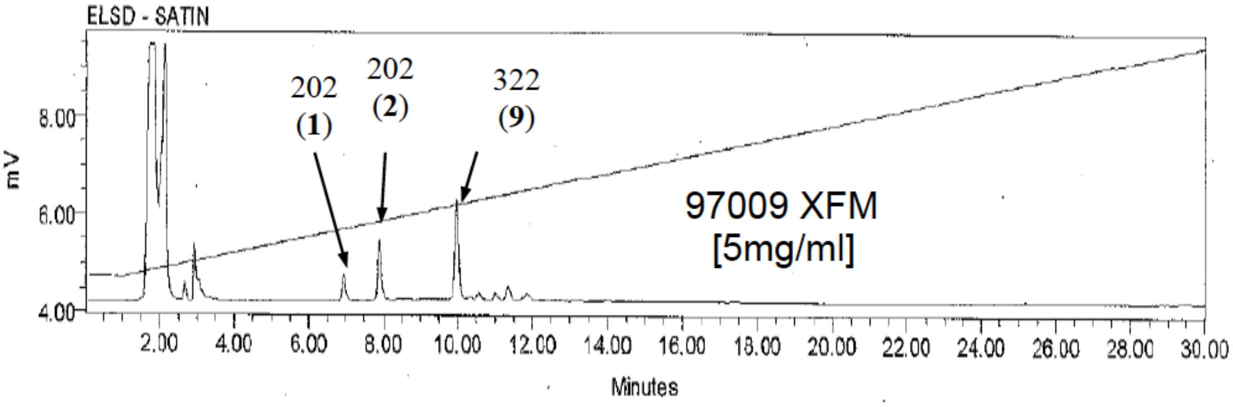

LC-ELSD chromatogram of 97009 XFM extract

Figure S1. The ^{1}H NMR spectrum of makaluvamine A (**1**) in DMSO-d_6 (500 MHz).

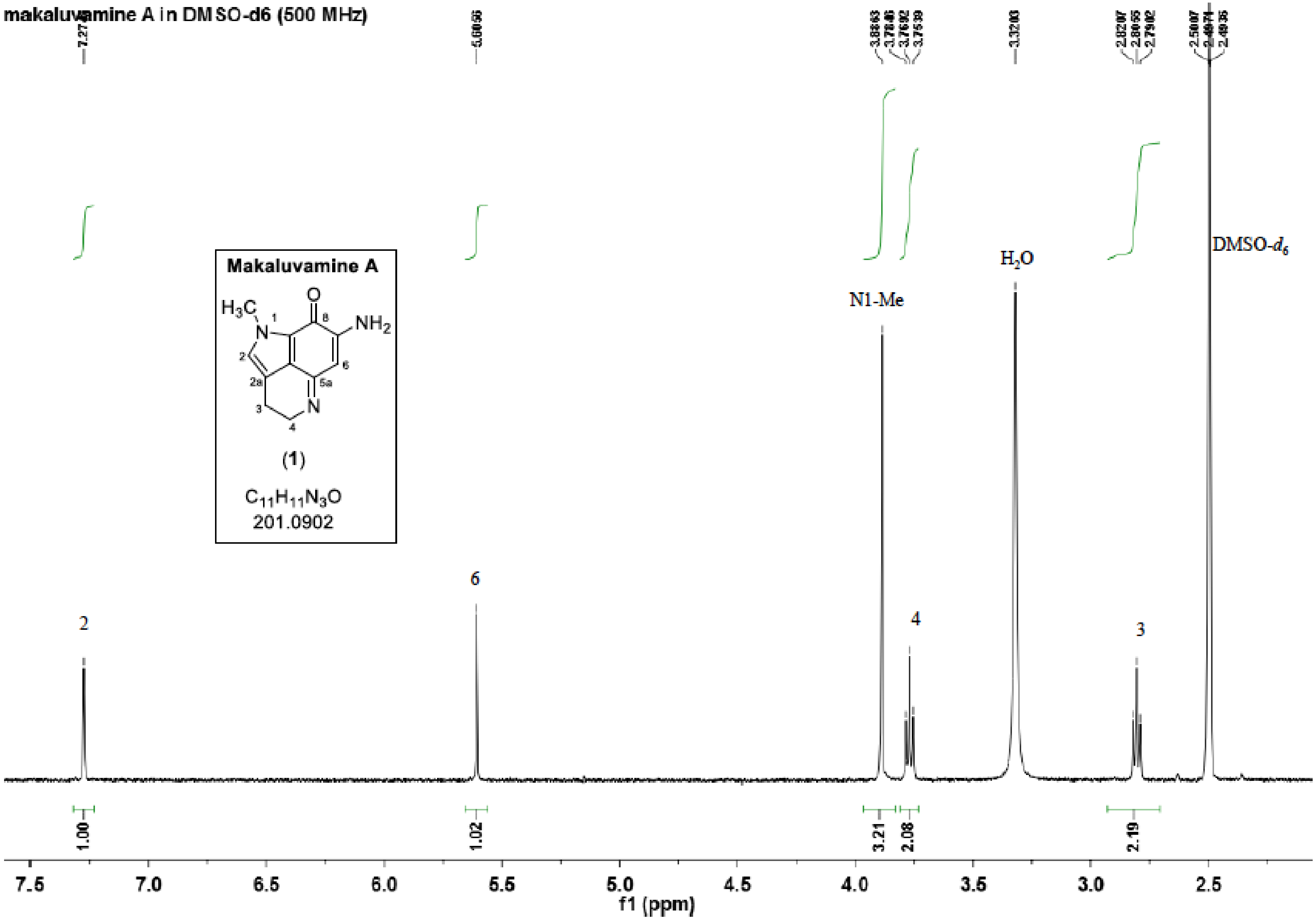

Figure S2. The ^{1}H NMR spectrum of makaluvamine C (**2**) in DMSO-d_6 (500 MHz).

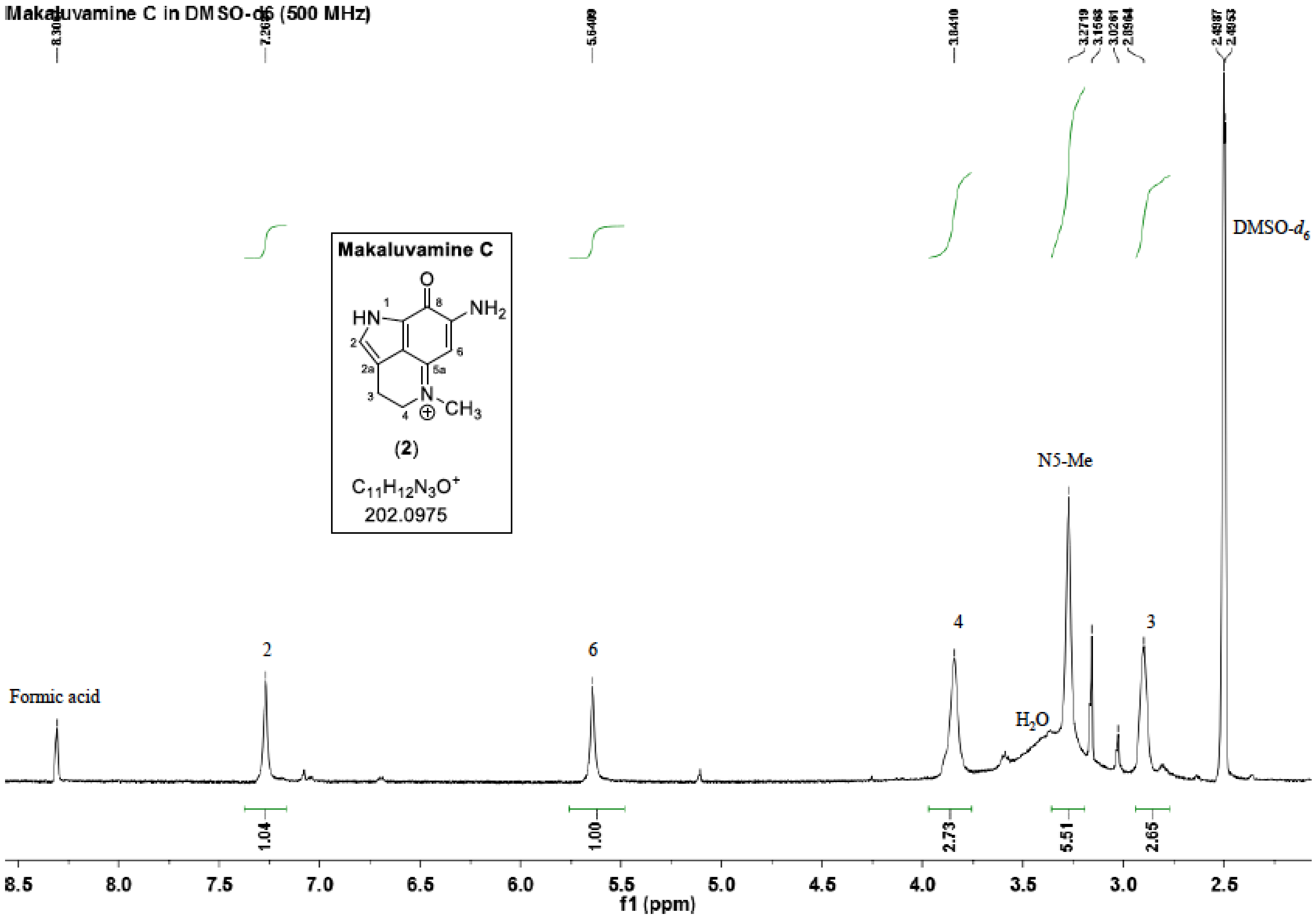

Figure S3. The ^{1}H NMR spectrum of damirone B (**3**) in DMSO-d_6 (600 MHz).

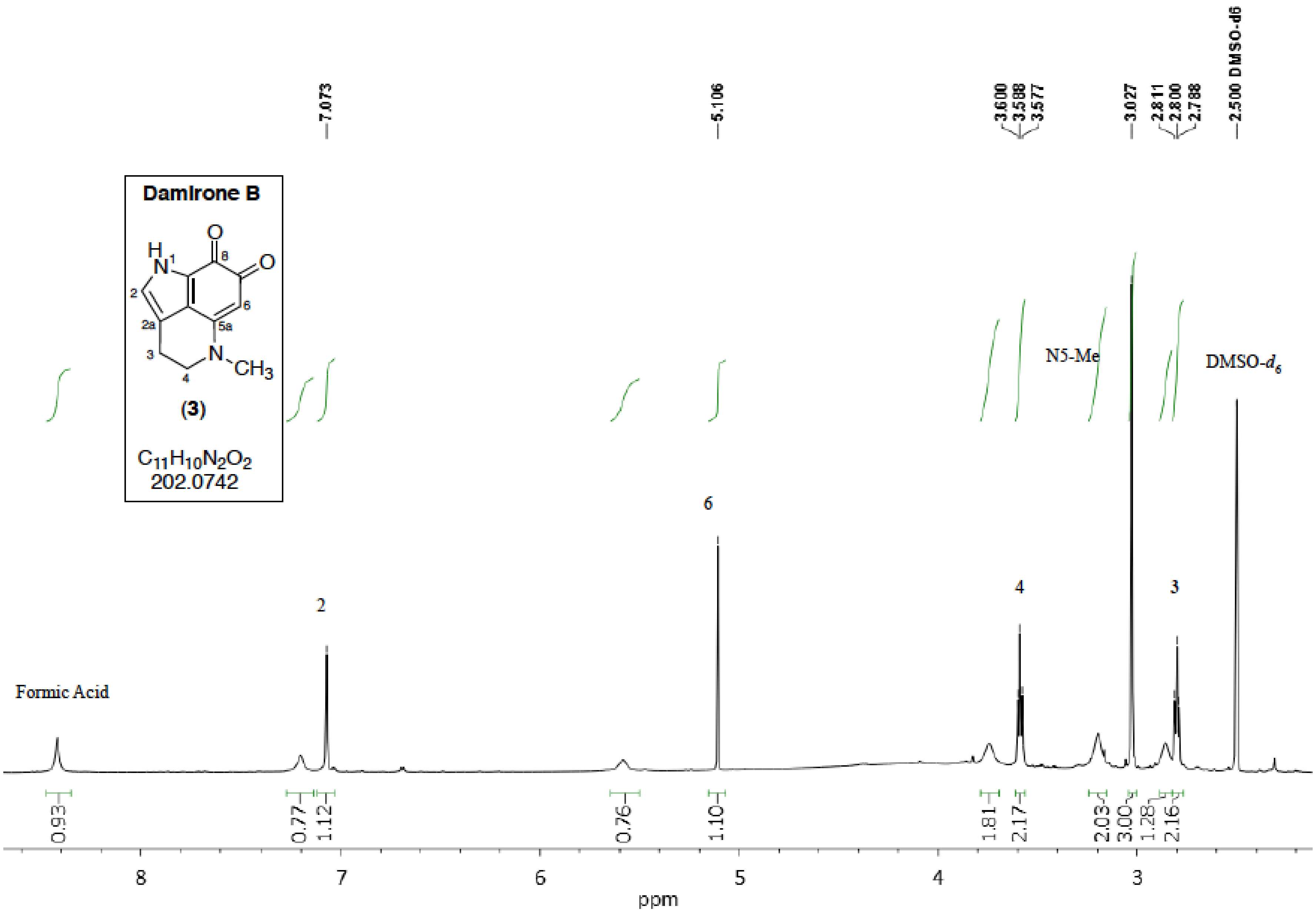

Figure S4. The ^{1}H NMR spectrum of makaluvamine H (**4**) in DMSO-*d*₆ (500 MHz).

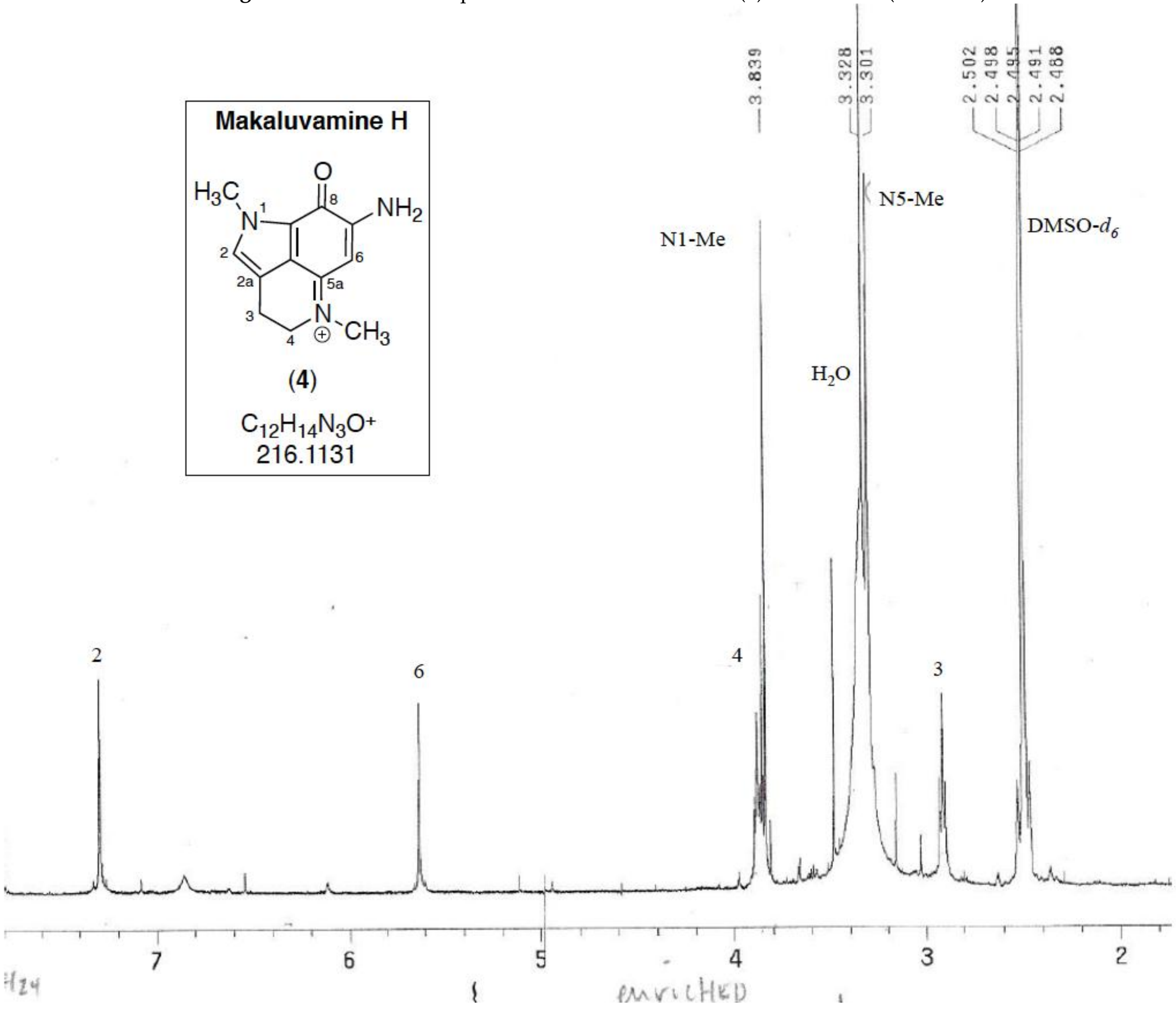

Figure S5. The ^{1}H NMR spectrum of damirone A (**5**) in DMSO-d_6 (500 MHz).

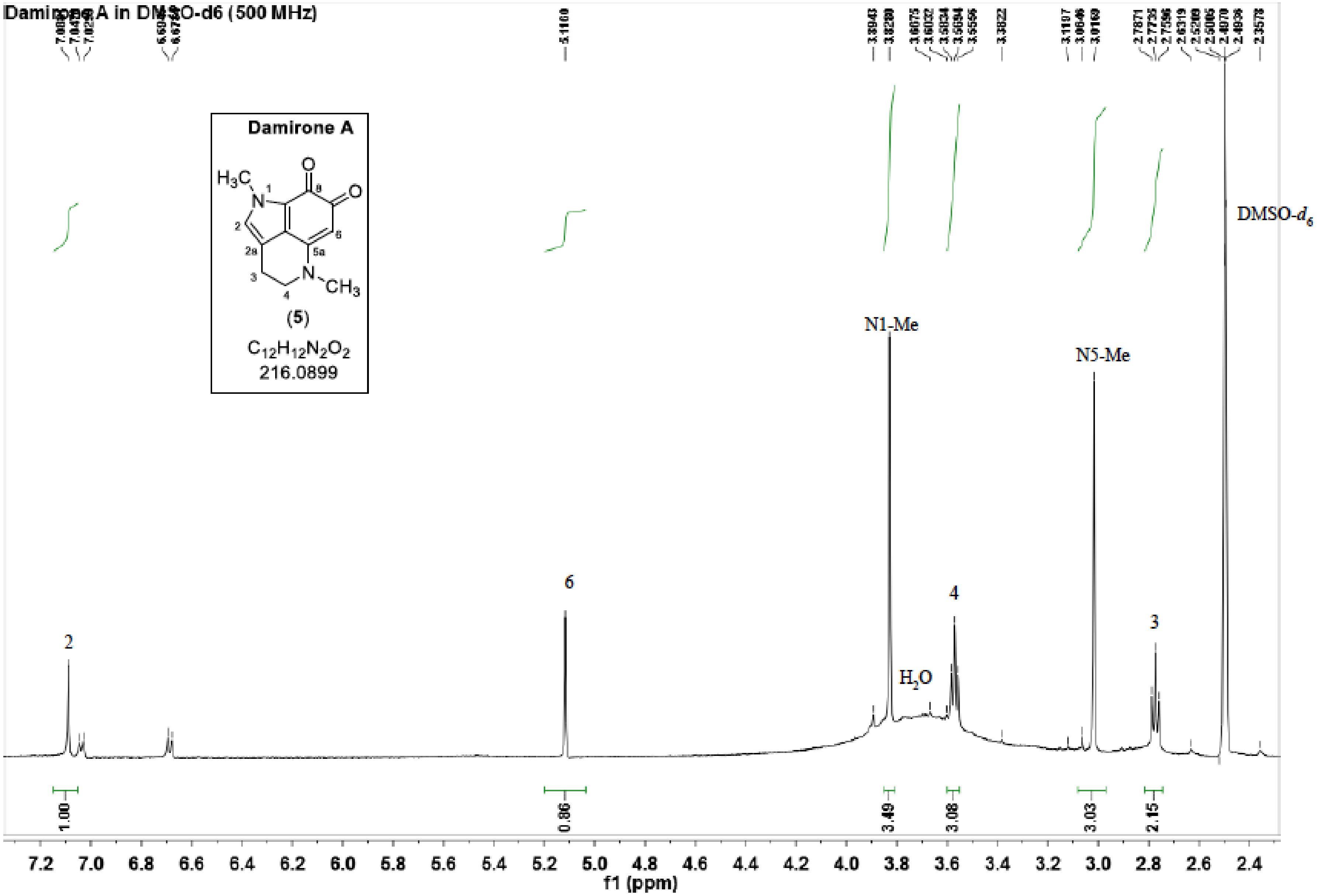

Figure S6. The ^{1}H NMR spectrum of damirone D (**6**) in DMSO-d_6 (500 MHz).

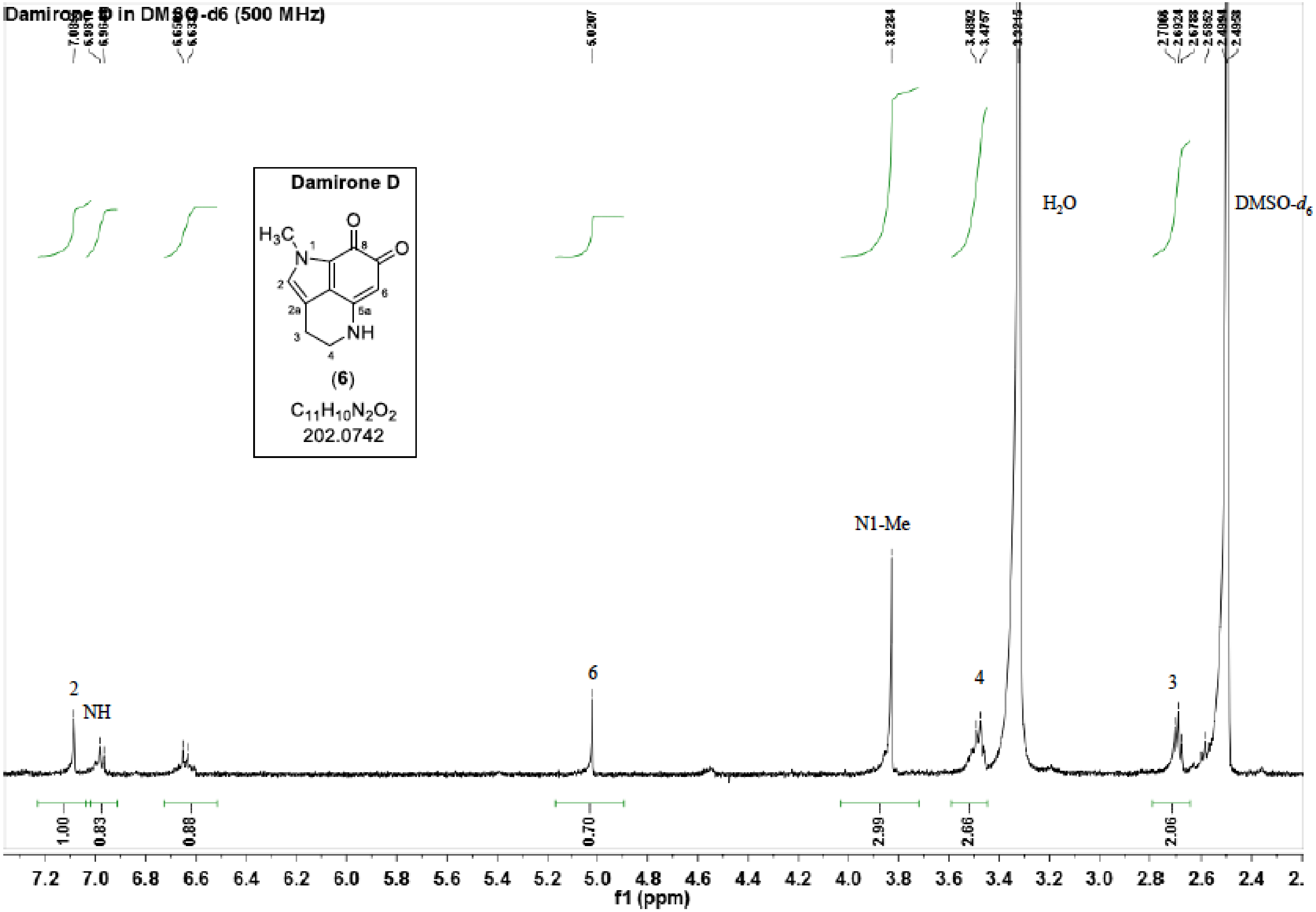

Figure S7. The ^1H NMR spectrum of makaluvamine D (**7**) in DMSO-d_6 (500 MHz).

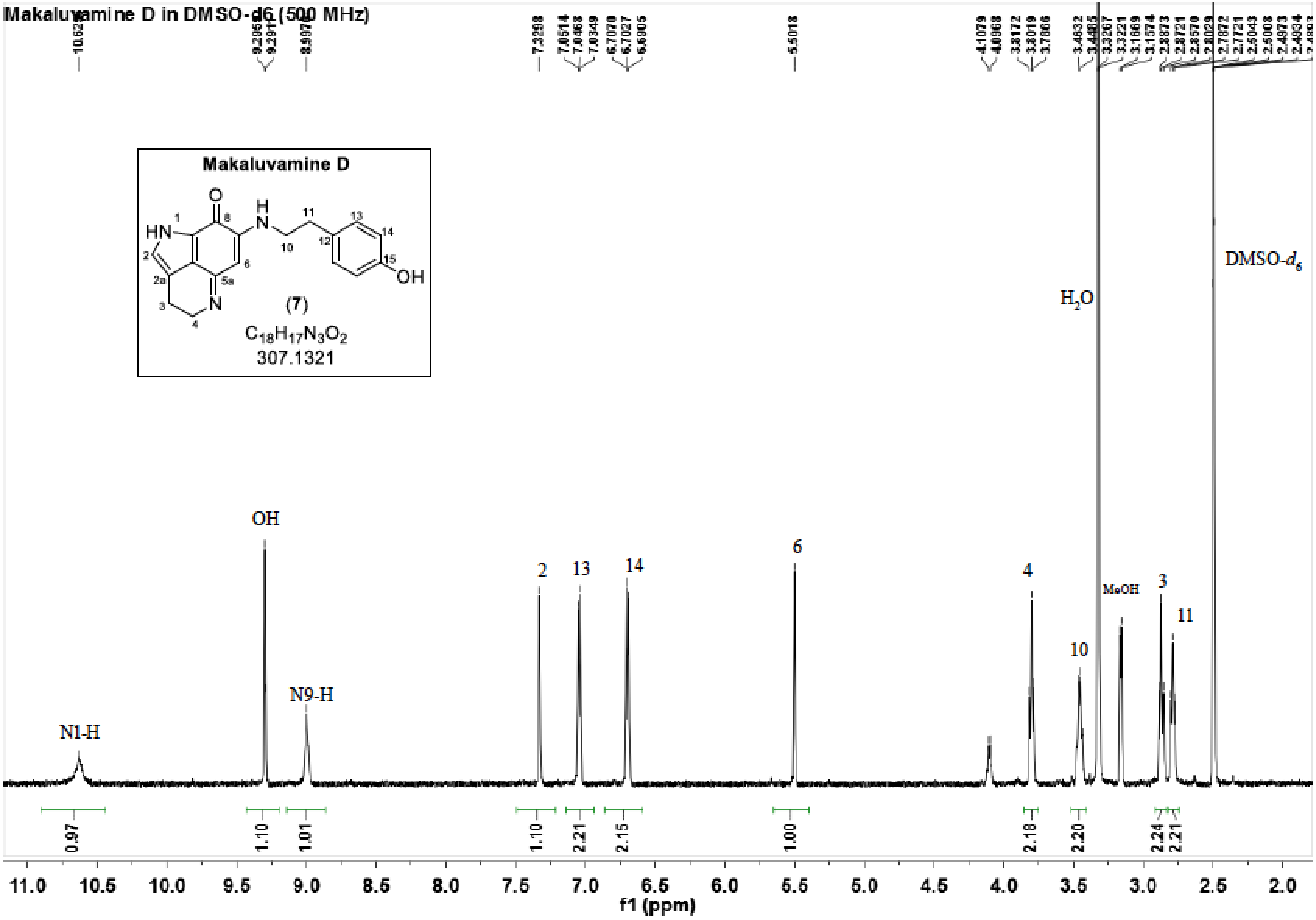

Figure S8. The ^{1}H NMR spectrum of makaluvamine G (**8**) in CD3OD (500 MHz).

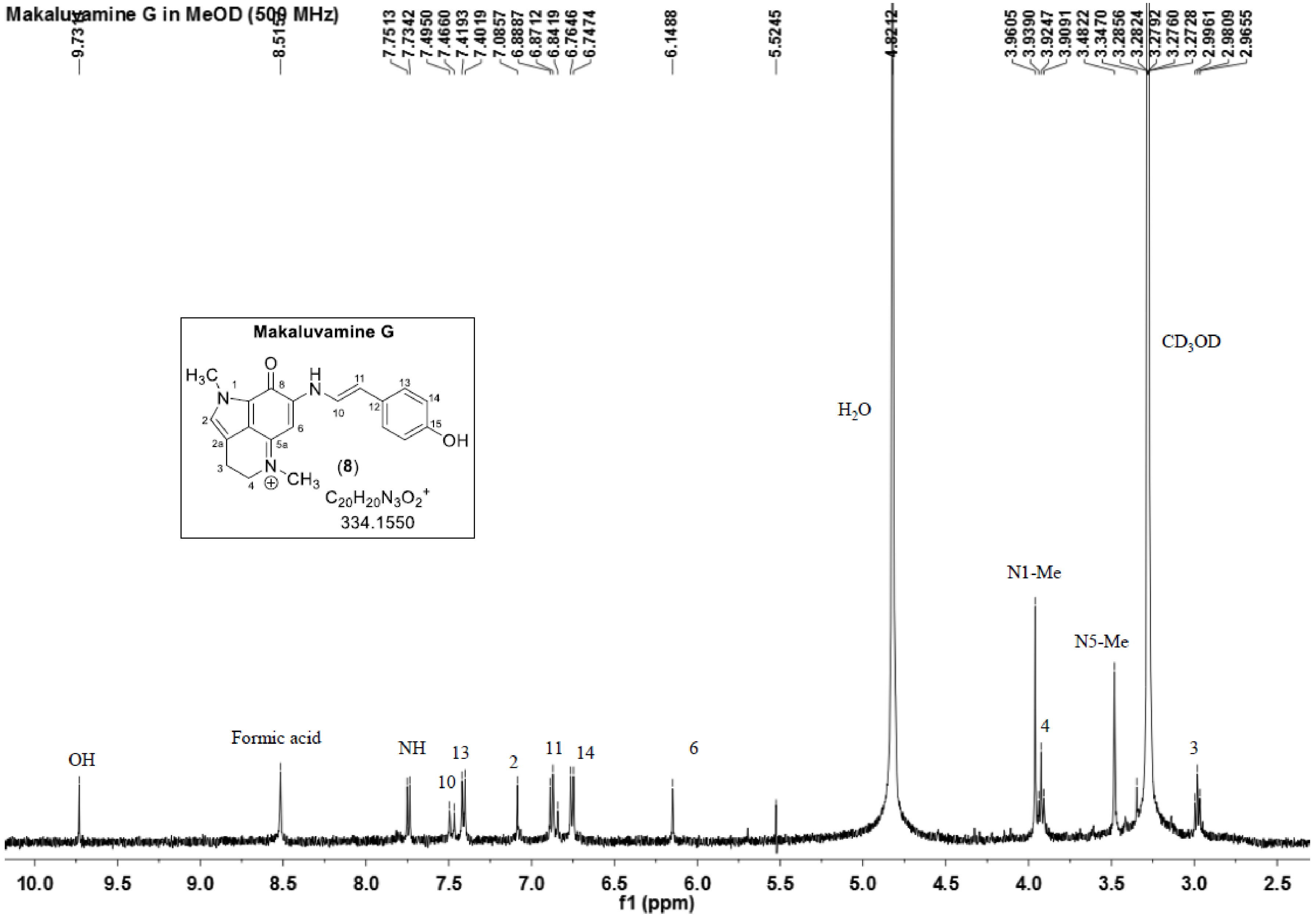

Figure S9. The ^{1}H NMR spectrum of makaluvamine J (**9**) in DMSO-d_6 (500 MHz).

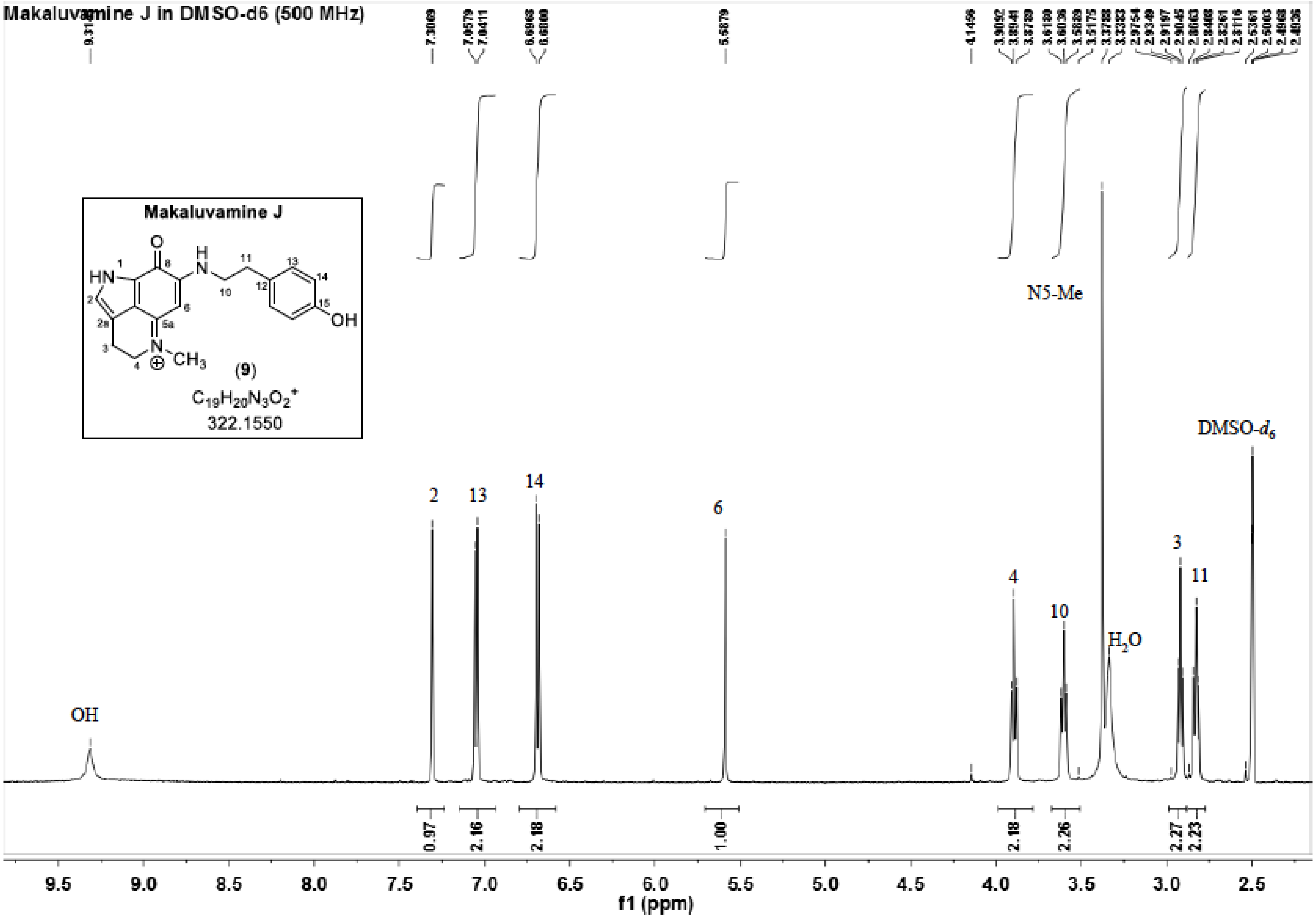

Figure S10. The ^{1}H NMR spectrum of makaluvamine K (**10**) in DMSO-d_6 (500 MHz).

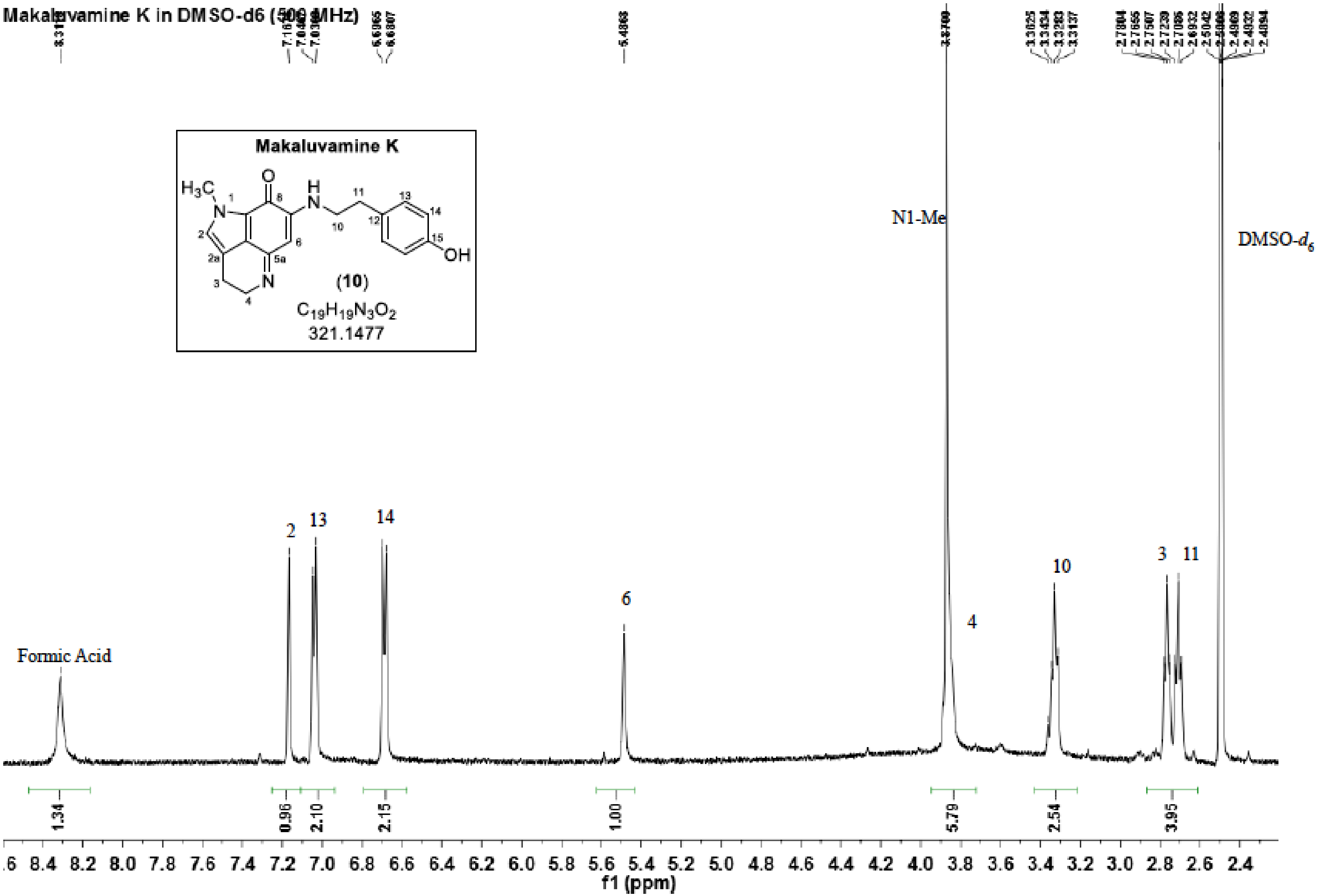

Figure S11. The ^{1}H NMR spectrum of makaluvamine L (**11**) in CD_3OD (500 MHz).

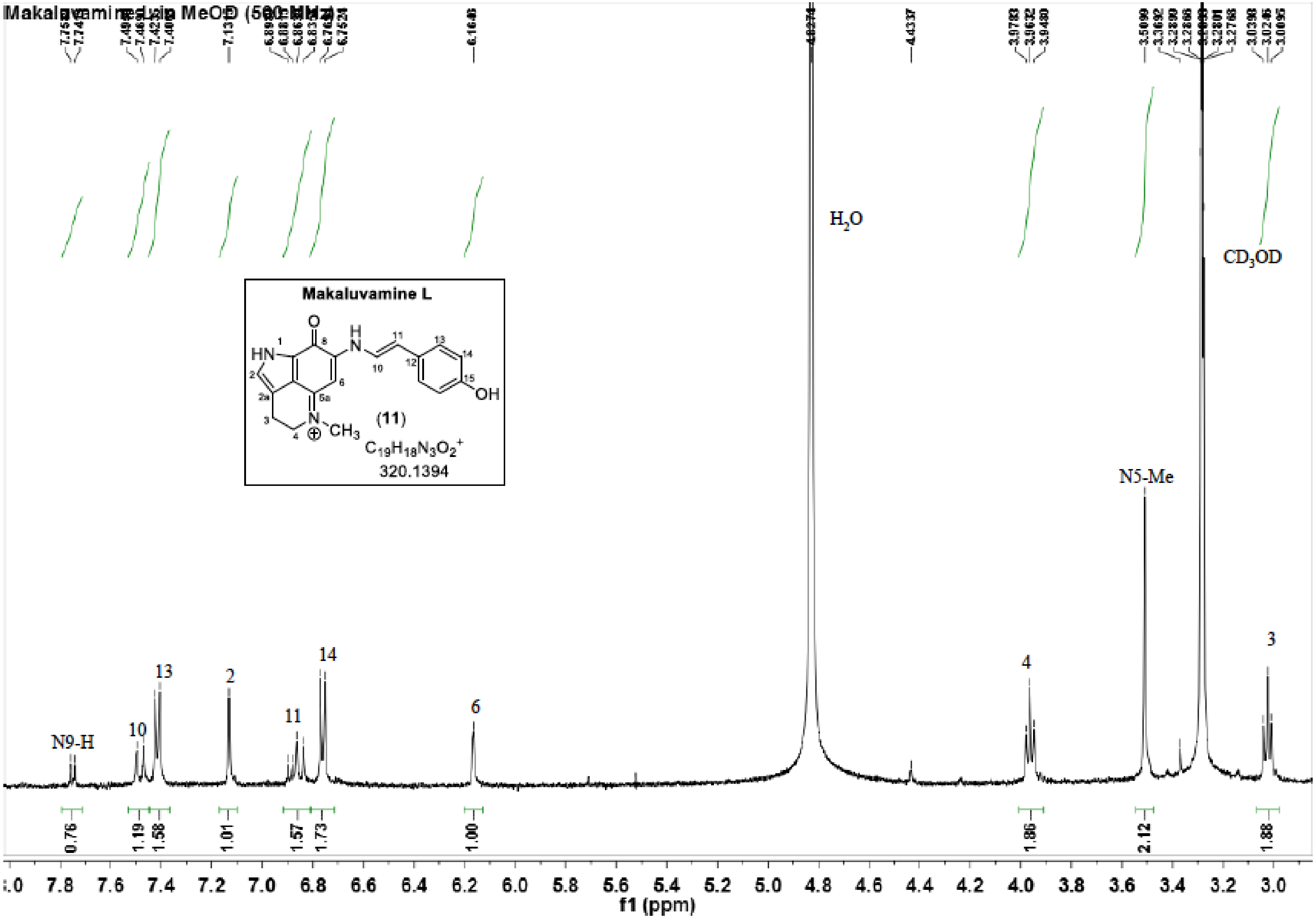

Figure S12. The ^{1}H NMR spectrum of makaluvamine P (**12**) in CH_3OD (500 MHz).

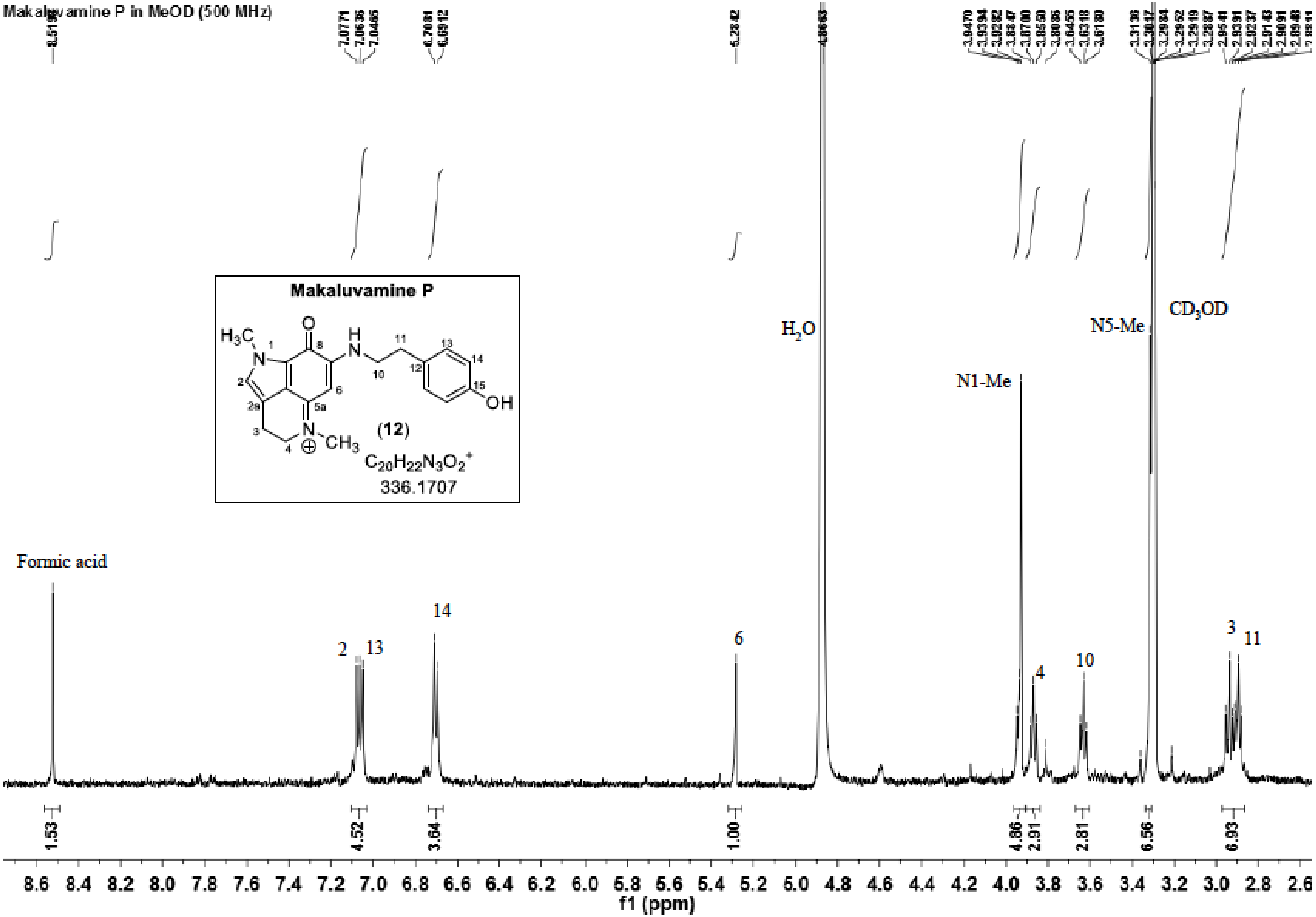

Figure S13. The ¹H NMR spectrum of 9-*N*-acetyl makaluvamine A (**13**) in DMSO-d_6 (500 MHz).

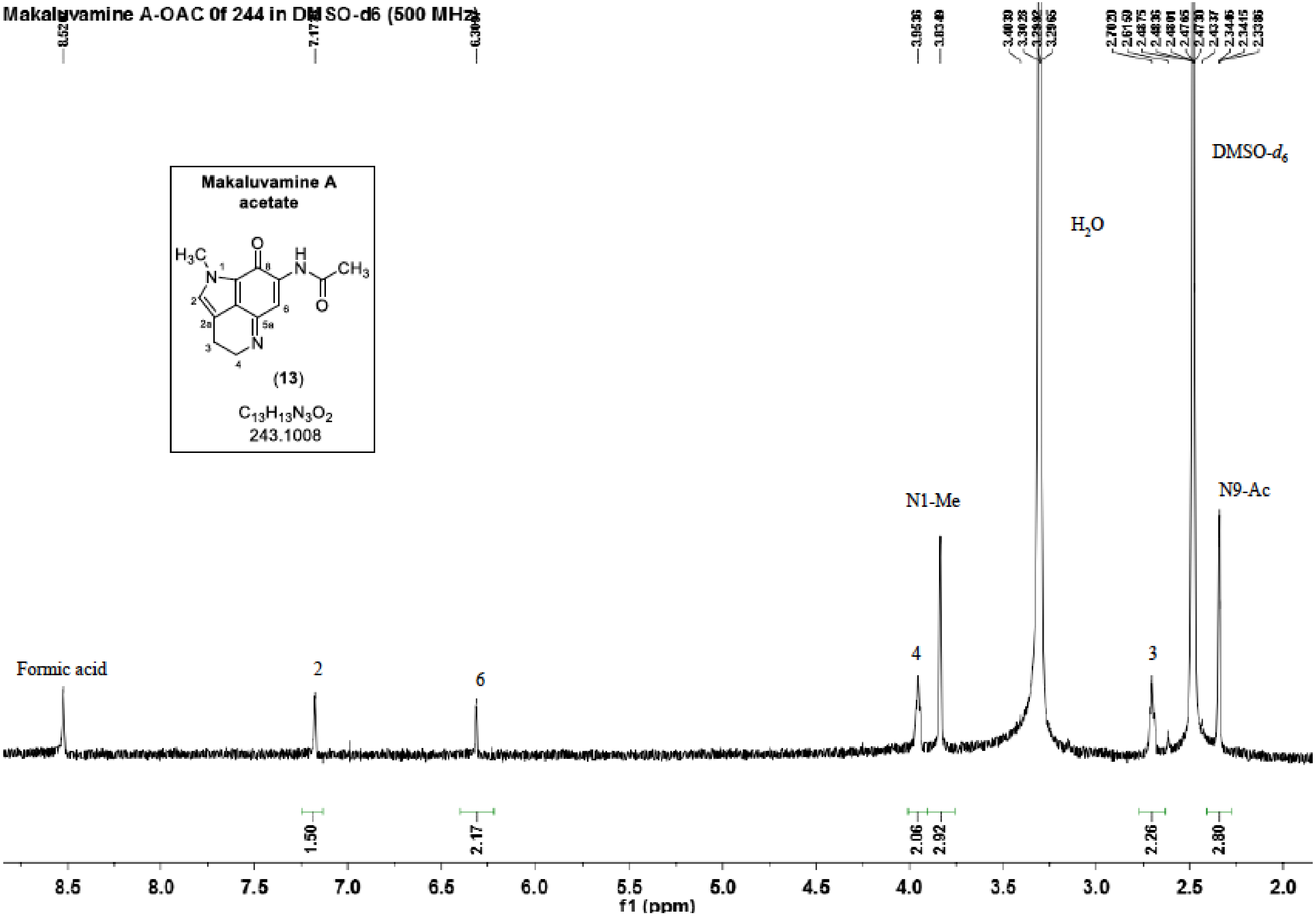

Figure S14. The ^1H NMR spectrum of 9-*N*-acetyl makaluvamine B (**14**) in CD_3OD (600 MHz).

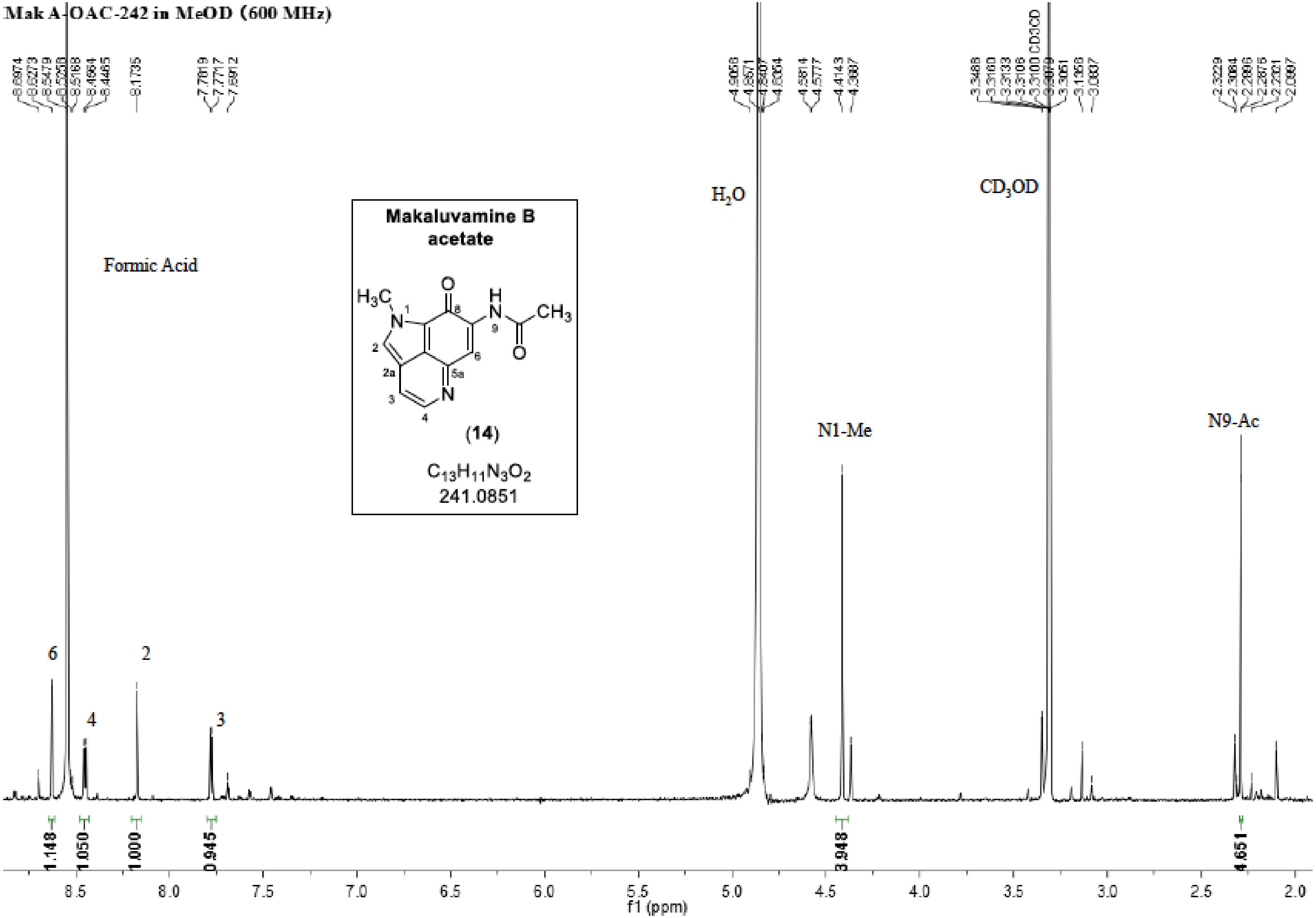

Figure S15. The ^{1}H NMR spectrum of 15-*O*-acetyl makaluvamine J (**15**) in DMSO-d_6 (500 MHz).

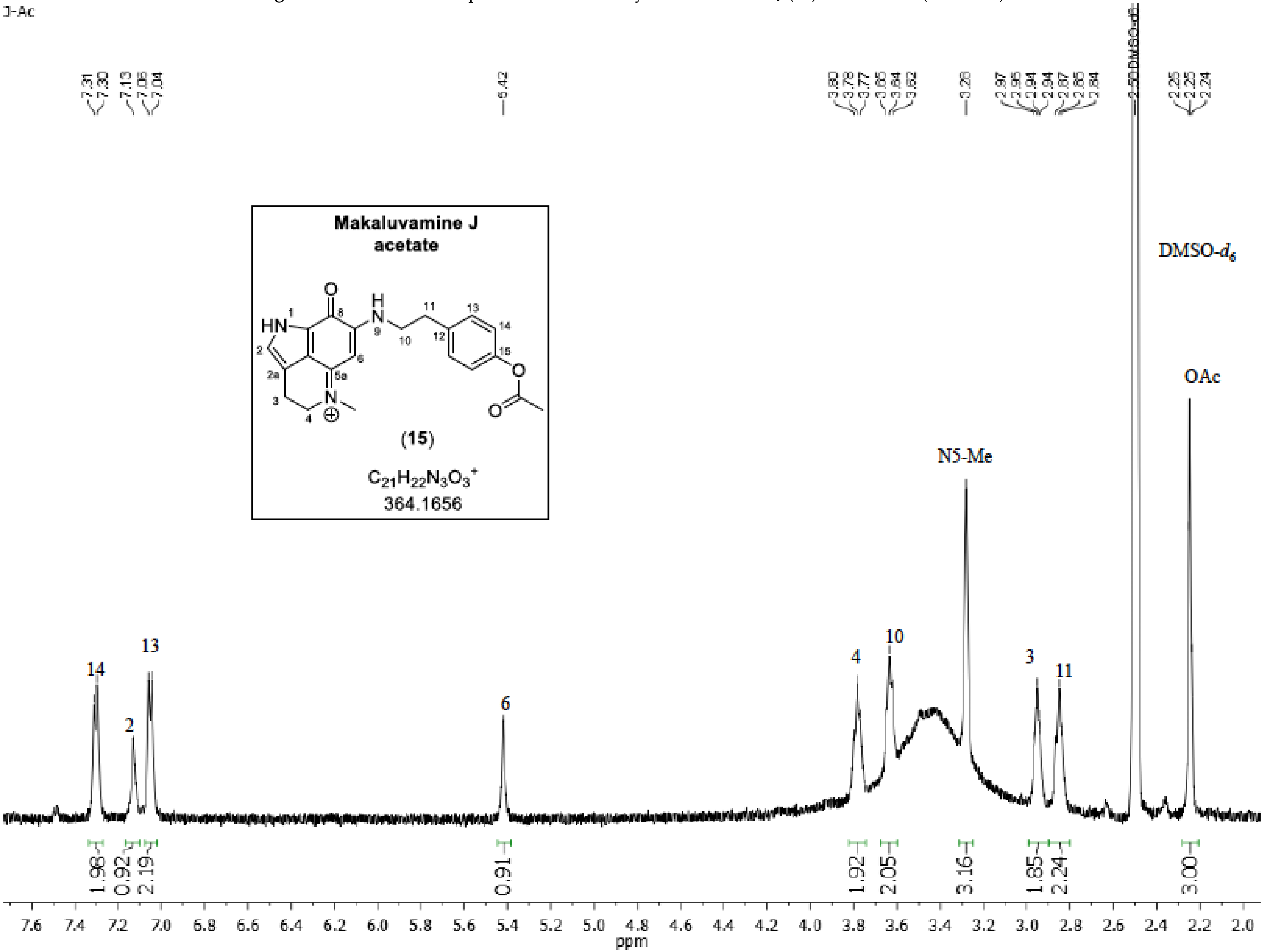

Figure S16. The ^1H NMR spectrum of 8,15-*O*-diacetyl-8-hydroxy-5a,7,8a-trien-makaluvamine J (**16**) in CD_3OD (800 MHz).

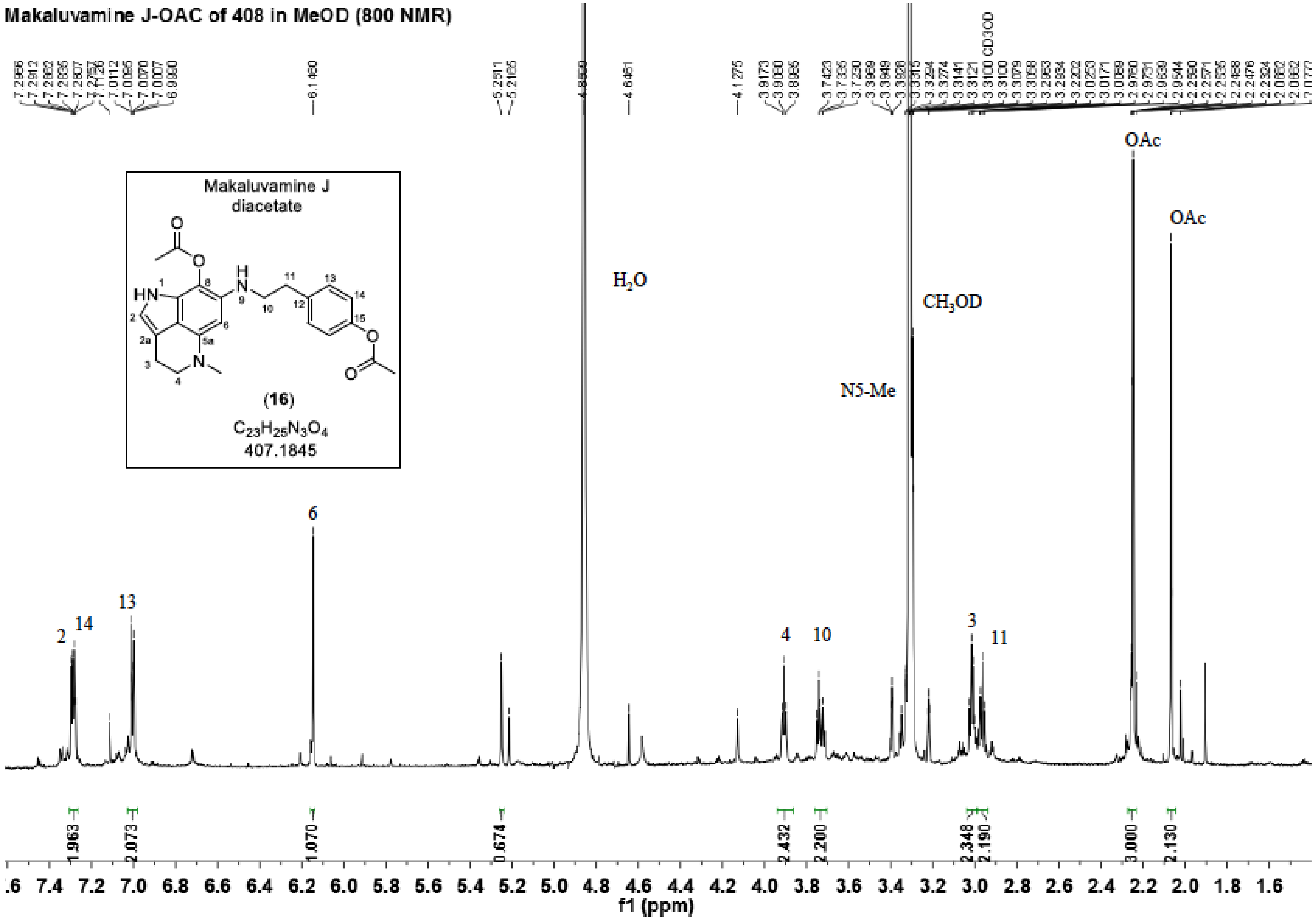

Table S1. 1H NMR data (500/600 MHz) for the makaluvamines (**1**, **2**, **4**, **7–12**) and damirones (**3**, **5**, **6**) in DMSO-d_6.

position	**Mak A (1)**	**Mak C (2)**	**Dam B (3)**	**Mak H (4)**	**Dam A (5)**	**Dam D (6)**	**Mak D (7)**	**Mak G (8)**[a]	**Mak J (9)**	**Mak K (10)**	**Mak L (11)** [a]	**Mak P (12)**[a]
2	7.27, s	7.27, s	7.07, s	7.30, s	7.09, s	7.09, s	7.33, s	7.09, s	7.31, s	7.17, s	7.13, s	7.08, s
3	2.81, t (7.5)	2.90, t (7.5)	2.80, t (7.0)	2.90, t	2.77, t (7.0)	2.69, t (7.0)	2.87, t (7.5)	2.98, t (7.5)	2.92, t (7.5)	2.77 t (7.5)	3.02, t (7.5)	2.94, t (7.5)
4	3.77, t (7.5)	3.84, t (7.5)	3.59, t (7.0)	3.90, m	3.57, t (7.0)	3.49, t (7.0)	3.80, t (7.5)	3.92, t (7.5)	3.89, t (7.5)	3.87, m	3.96, t (7.5)	3.87, t (7.5)
6	5.61, s	5.64, s	5.11, s	5.63, s	5.12, s	5.02, s	5.50, s	6.15, s	5.59, s	5.49, s	6.16, s	5.29, s
10							3.45, t (7.5)	7.48, d (14.5)	3.60, t (7.0)	3.33, brt (7.5)	7.47, d (13.5)	3.63, t (7.5)
11							2.80, t (7.5)	6.85, d (14.5)	2.83, t (7.0)	2.70, t (7.5)	6.84, d (13.5)	2.89, t (7.5)
13							7.05, d (8.5)	7.41, d (8.5)	7.05, d (8.0)	7.04, d (8.0)	7.41, d (8.5)	7.00, d (8.0)
14							6.70, d (8.5)	6.75, d (8.5)	6.69, d (8.0)	6.69, d (8.0)	6.76, d (8.5)	7.05, d (8.0)
N1-Me	3.89, s			3.84, s	3.83, s	3.83, s		3.96, s		3.87, s		3.93, s
N5-Me		3.27, s	3.03, s	3.33, s	3.02, s			3.48, s	3.37, s		3.51, s	3.31, s
N9-H							9.00, br	7.74, d (8.6)				
NH						6.97, d (8.7)	10.63, br				7.8, d (8.8)	
Ar-OH							9.29, s	9.73, s	9.31, s			

[a] ^{1}H NMR was measured in CD_3OD

Figure S17. MS^2 spectrum and predicted fragmentation structures of makaluvamine A (**1**).

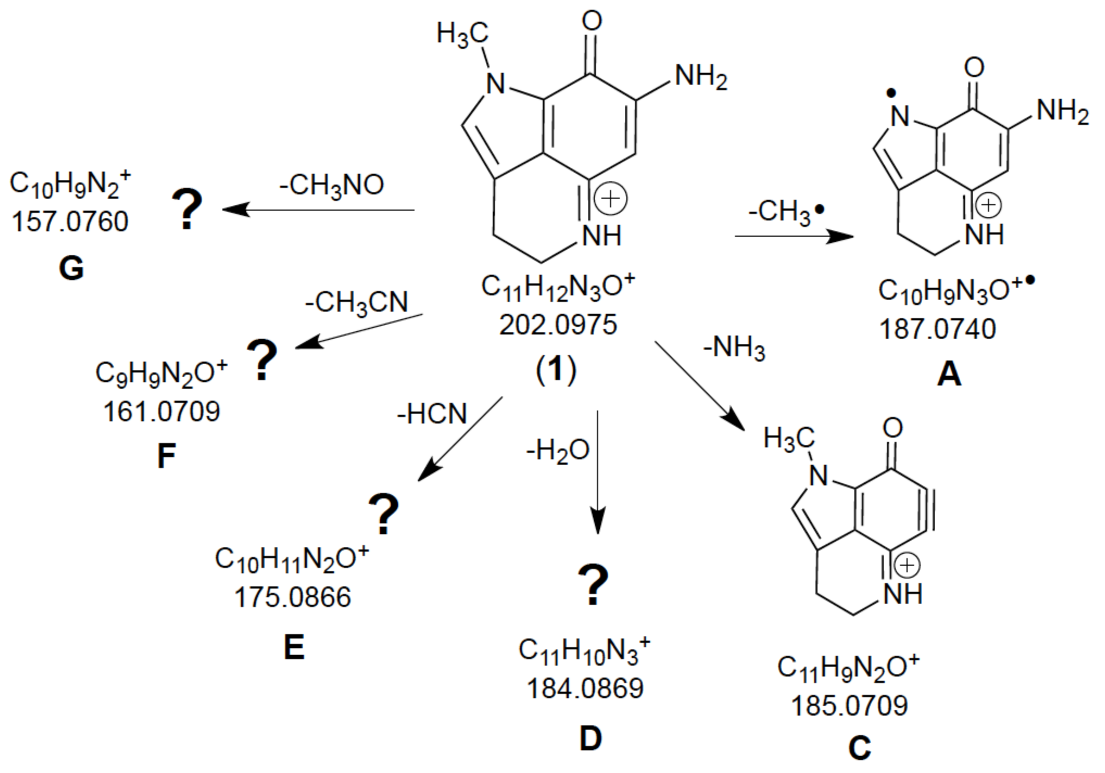

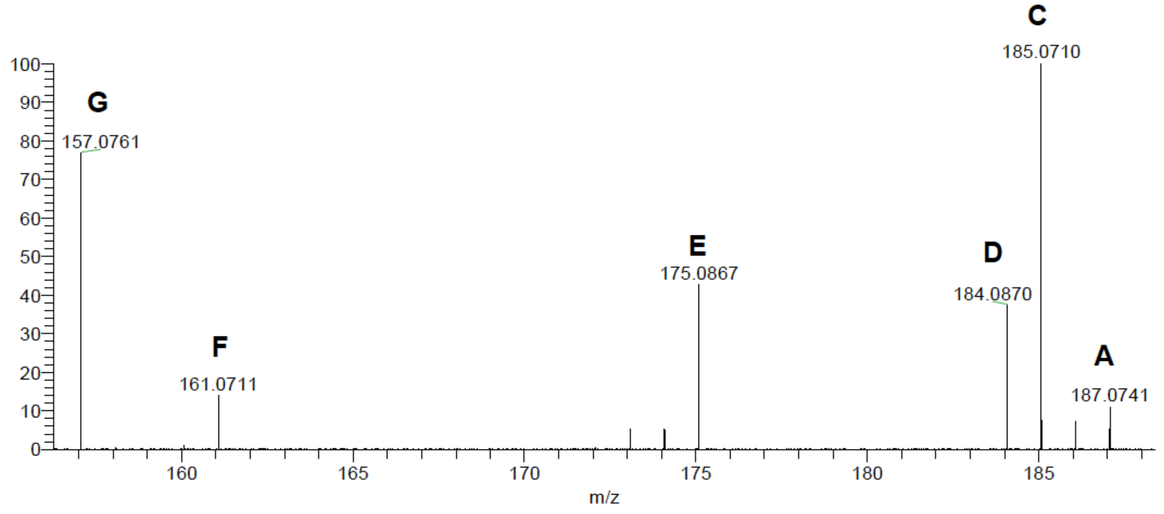

Figure S18. MS^2 spectrum and predicted fragmentation structures of makaluvamine C (**2**).

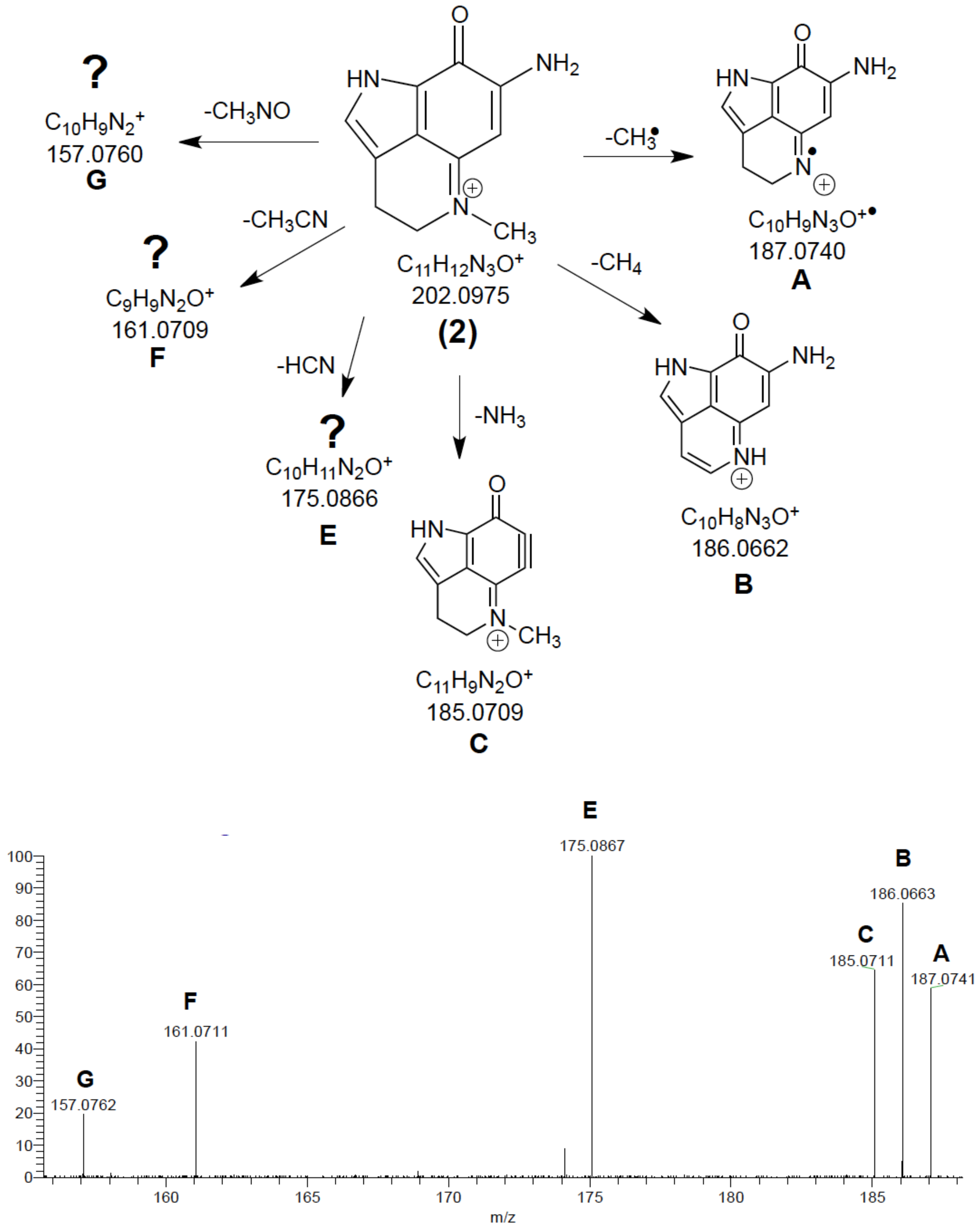

Figure S19. MS^2 spectrum and predicted fragmentation structures of damirone B (**3**).

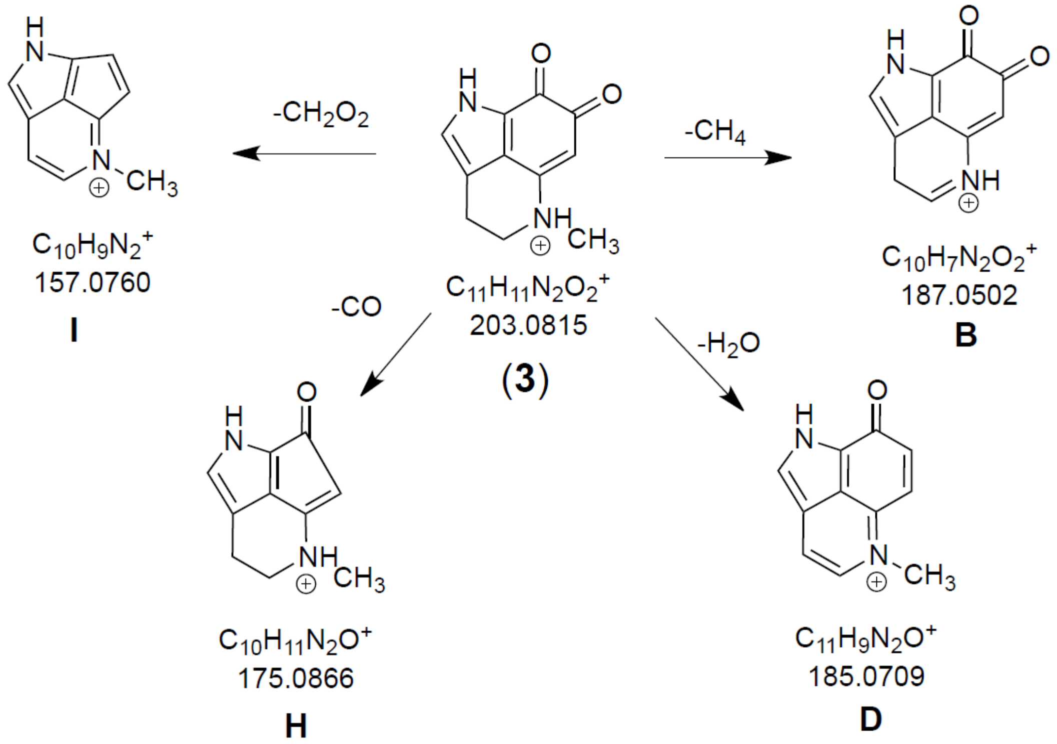

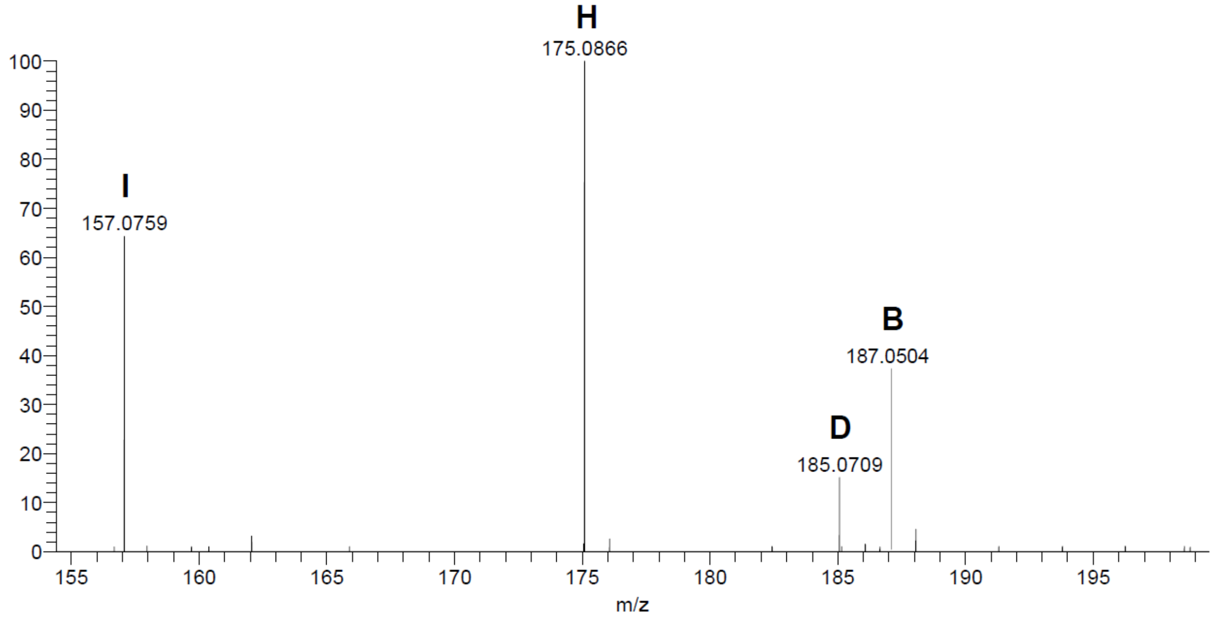

Figure S20. MS² spectrum and predicted fragmentation structures of makaluvamine H (**4**).

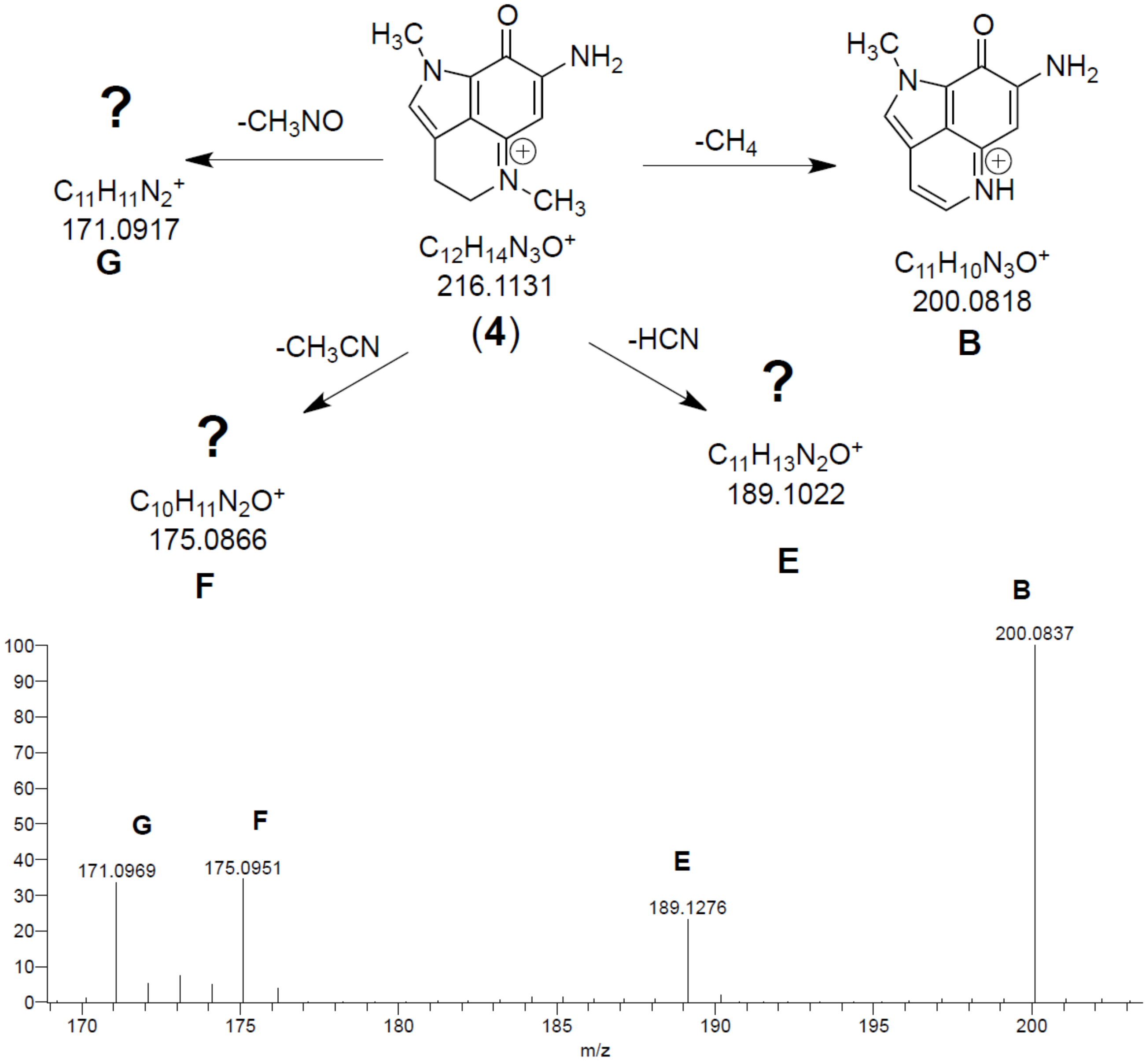

Figure S21. MS^2 spectrum and predicted fragmentation structures of damirone A (**5**).

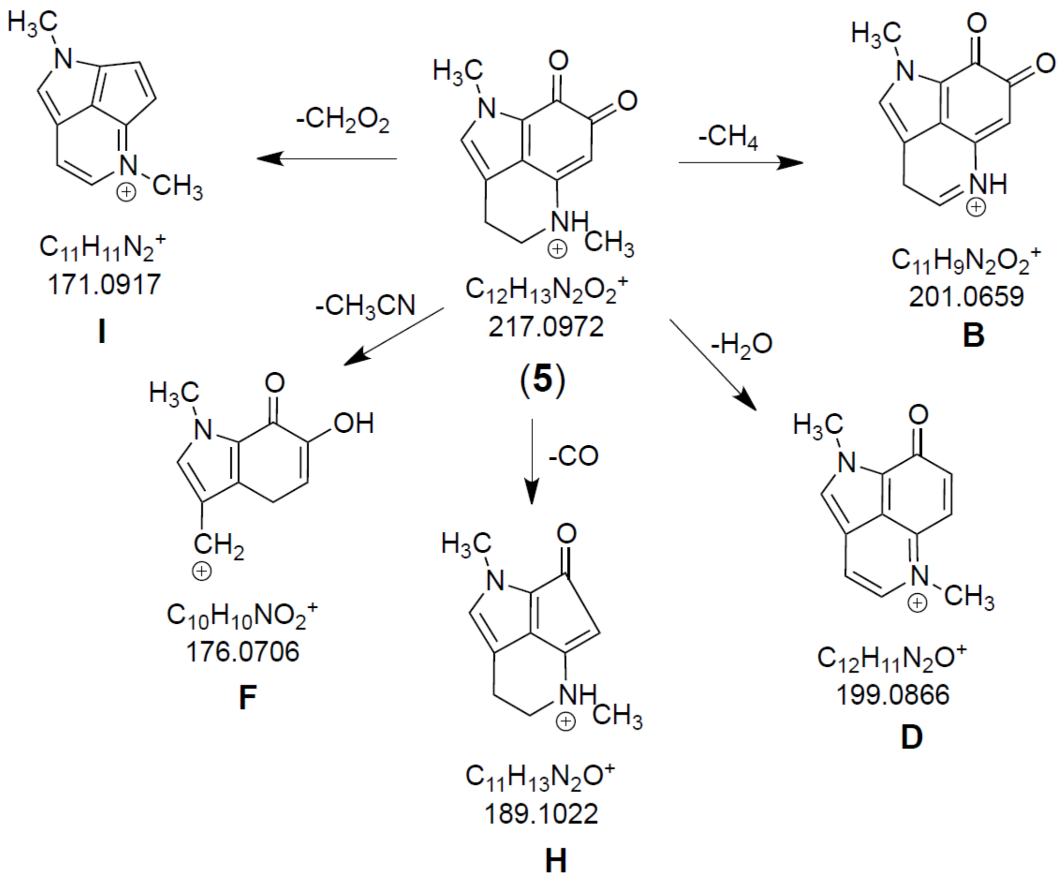

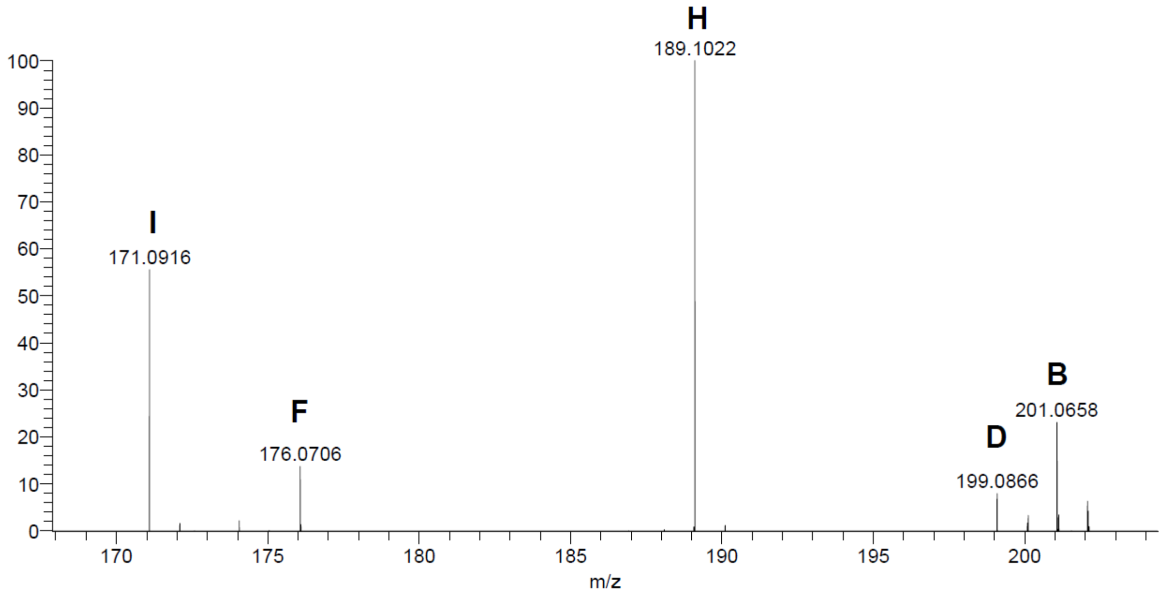

Figure S22. MS[2] spectrum and predicted fragmentation structures of damirone D (**6**).

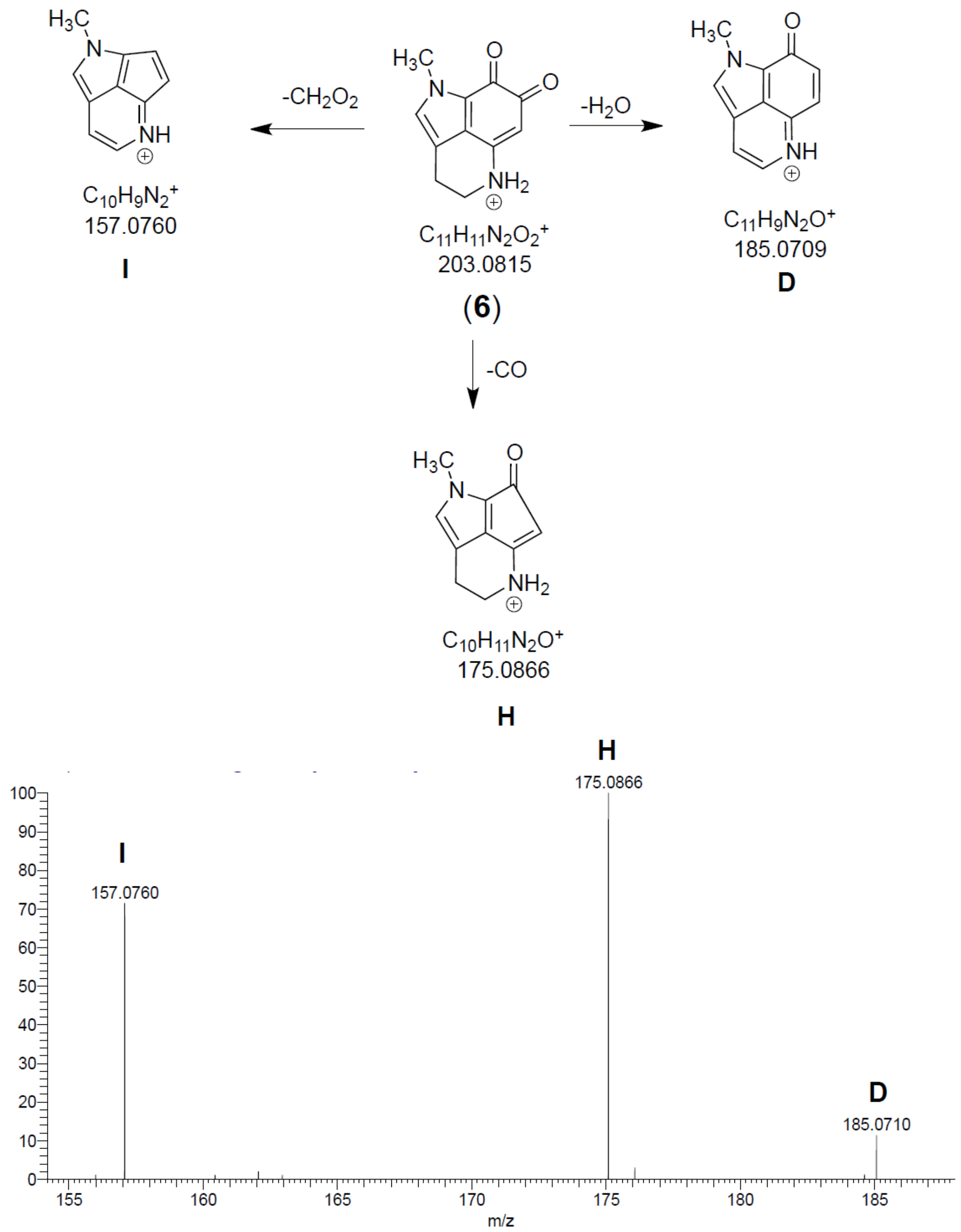

Figure S23. MS² spectrum and predicted fragmentation structures of makaluvamine D (**7**).

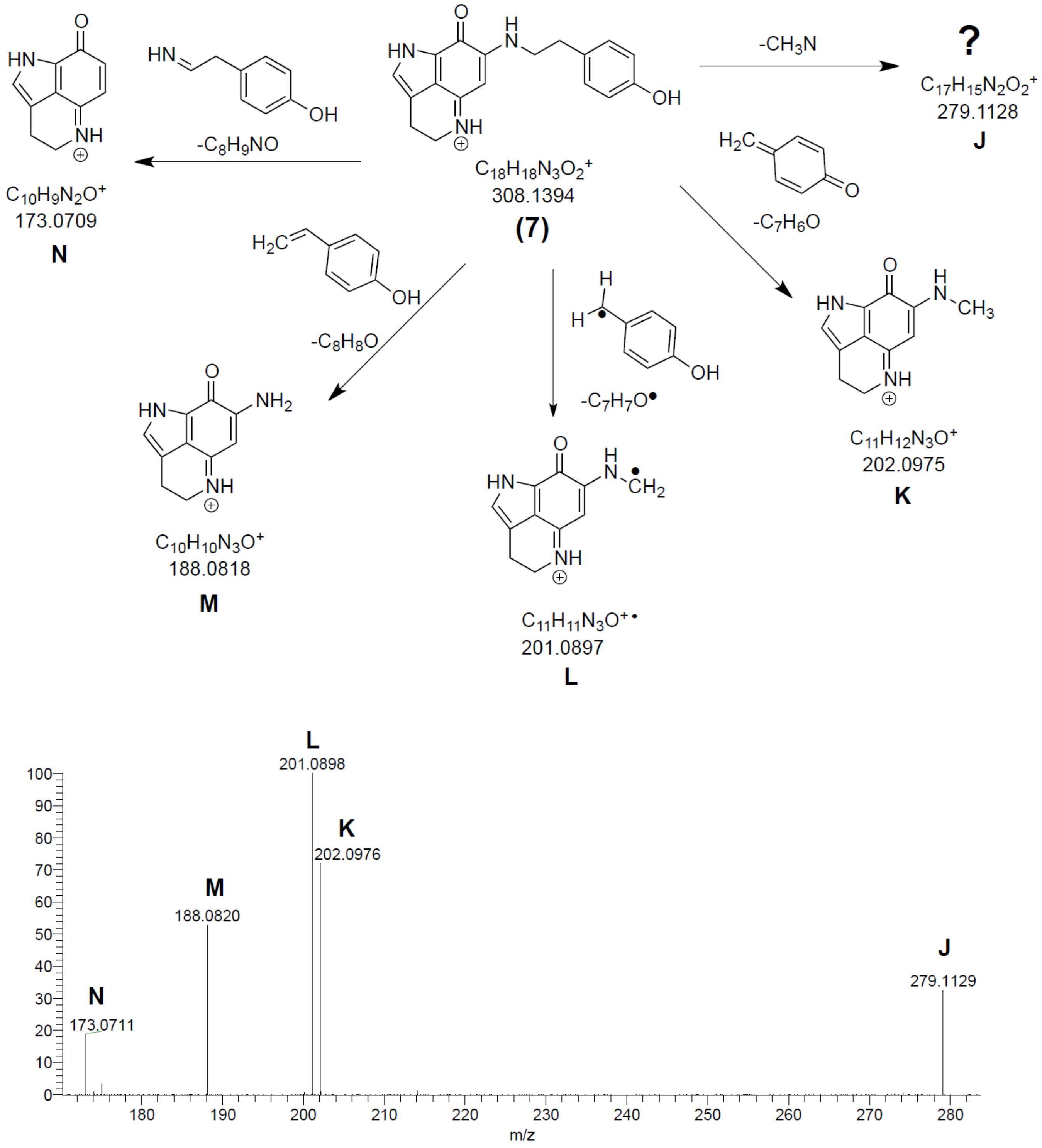

Figure S24. MS² spectrum and predicted fragmentation structures of makaluvamine G (**8**).

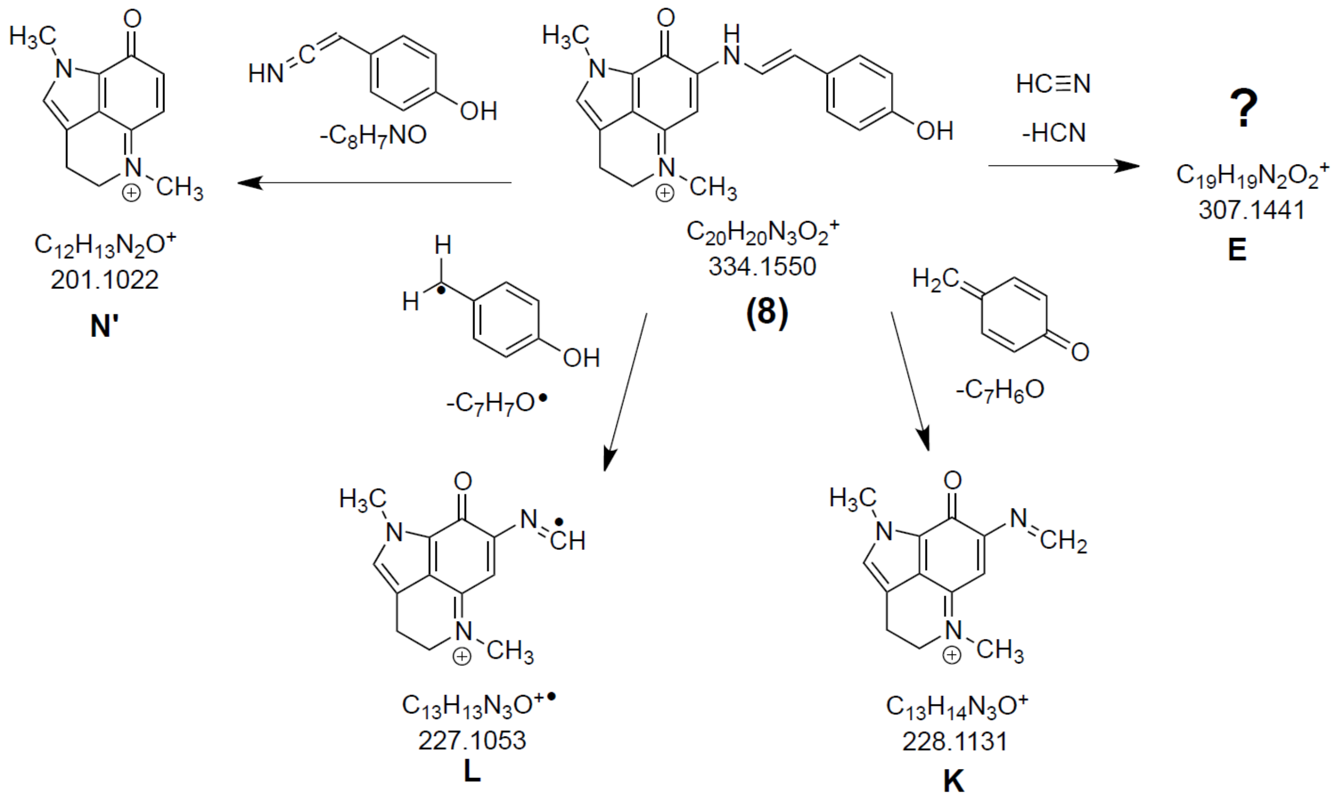

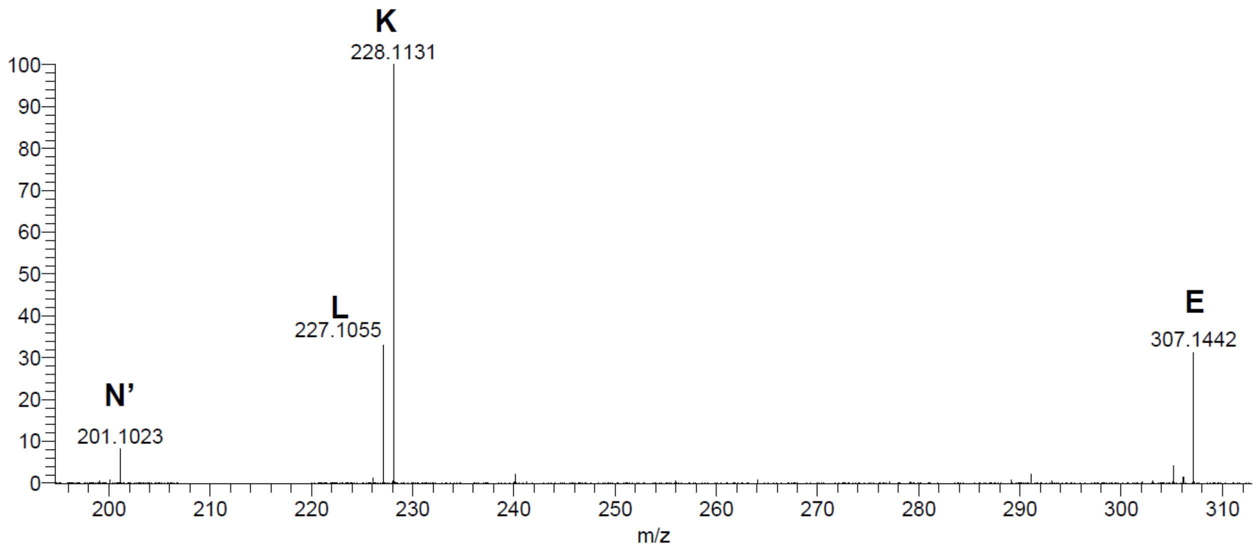

Figure S25. MS^2 spectrum and predicted fragmentation structures of makaluvamine J (**9**).

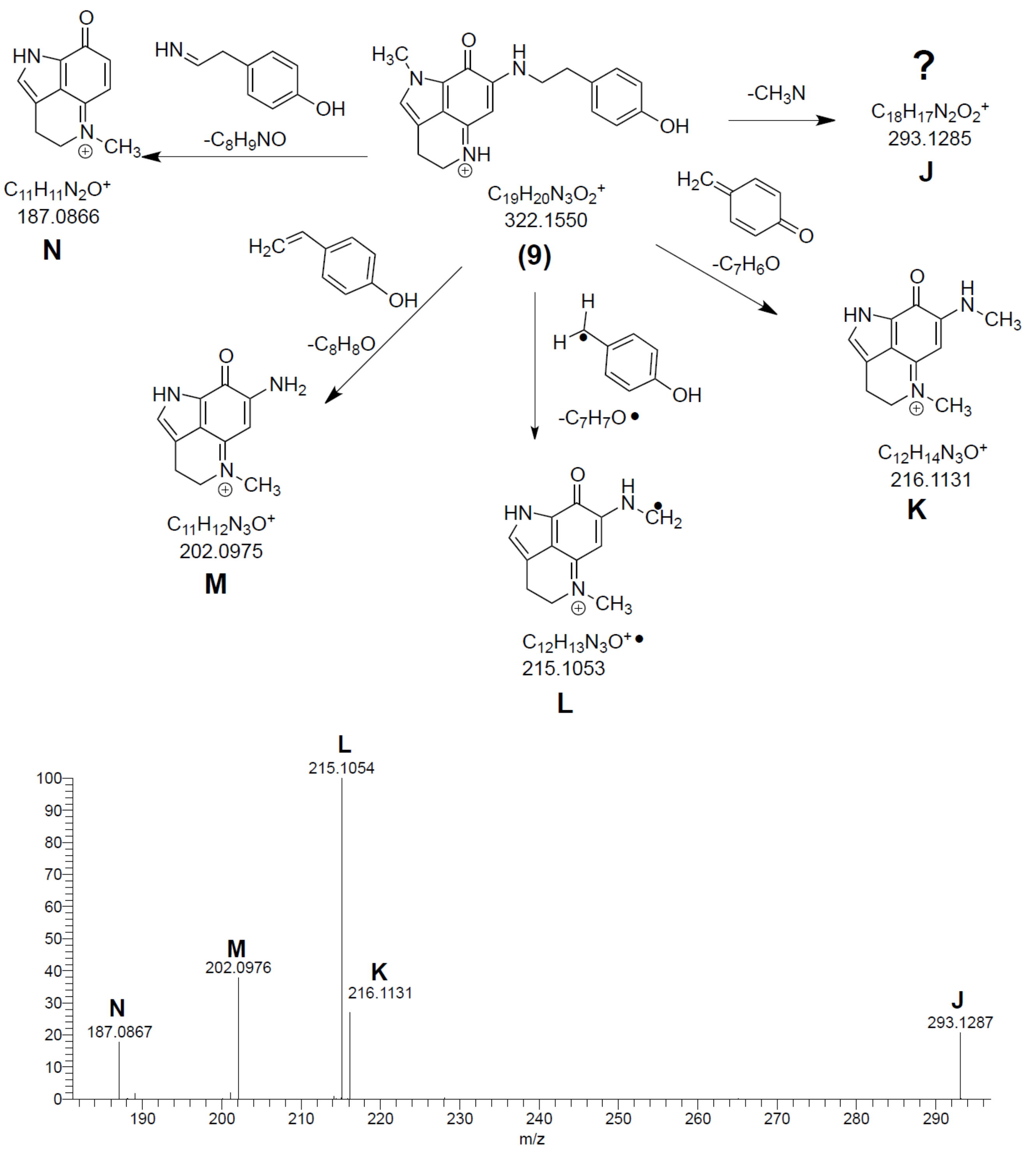

Figure S26. MS^2 spectrum and predicted fragmentation structures of makaluvamine K (**10**).

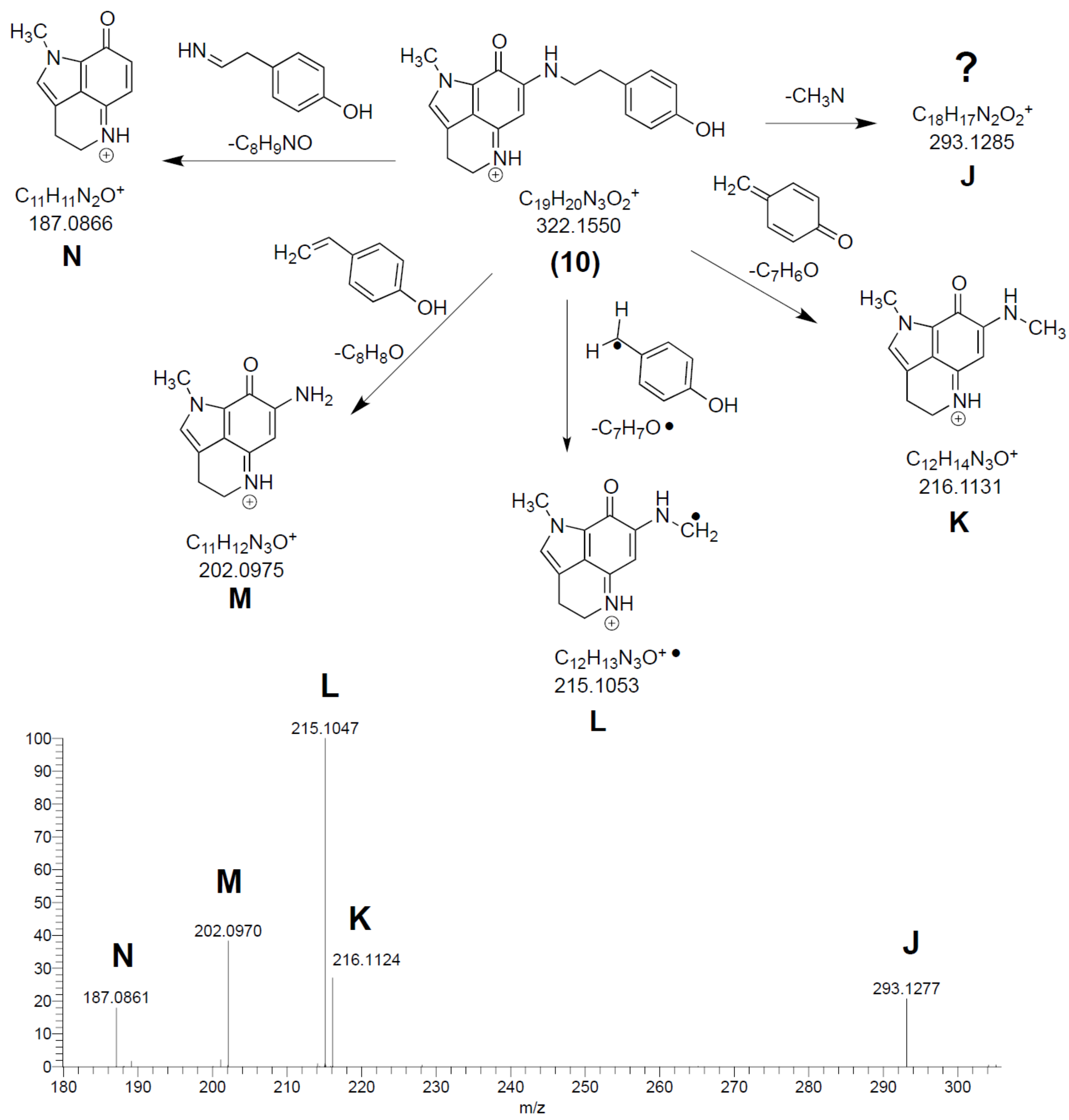

Figure S27. MS² spectrum and predicted fragmentation structures of makaluvamine L (**11**).

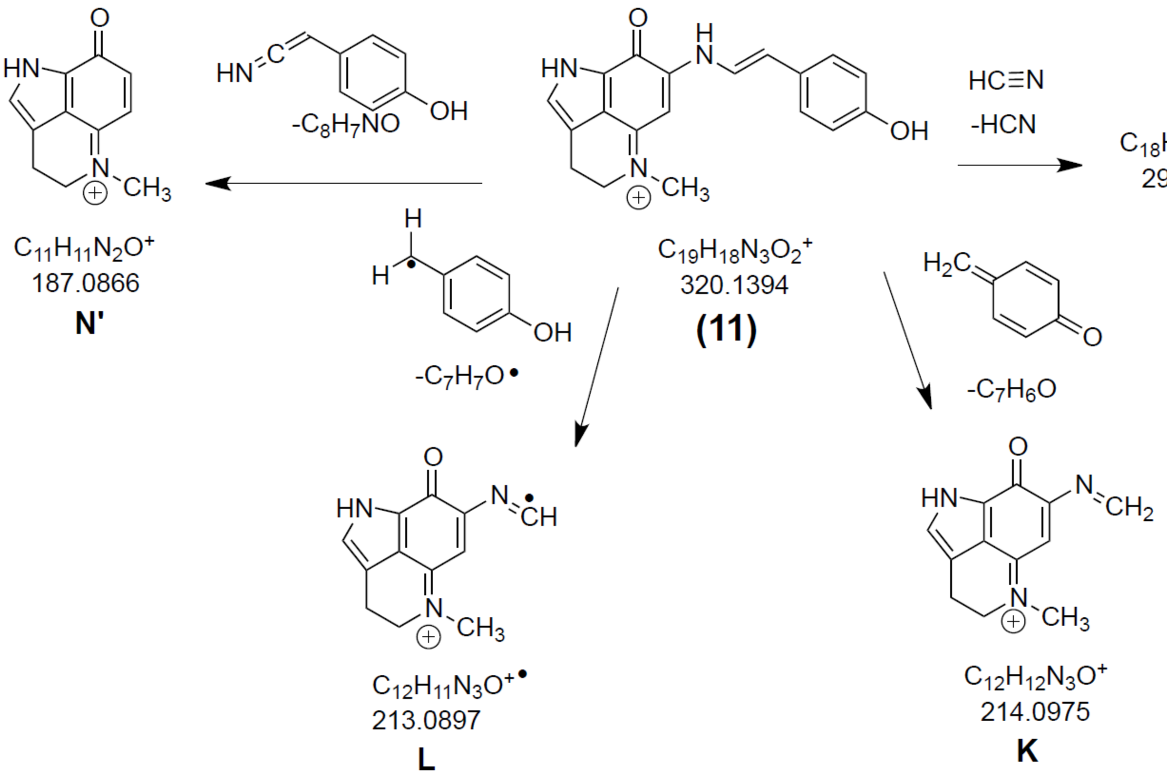

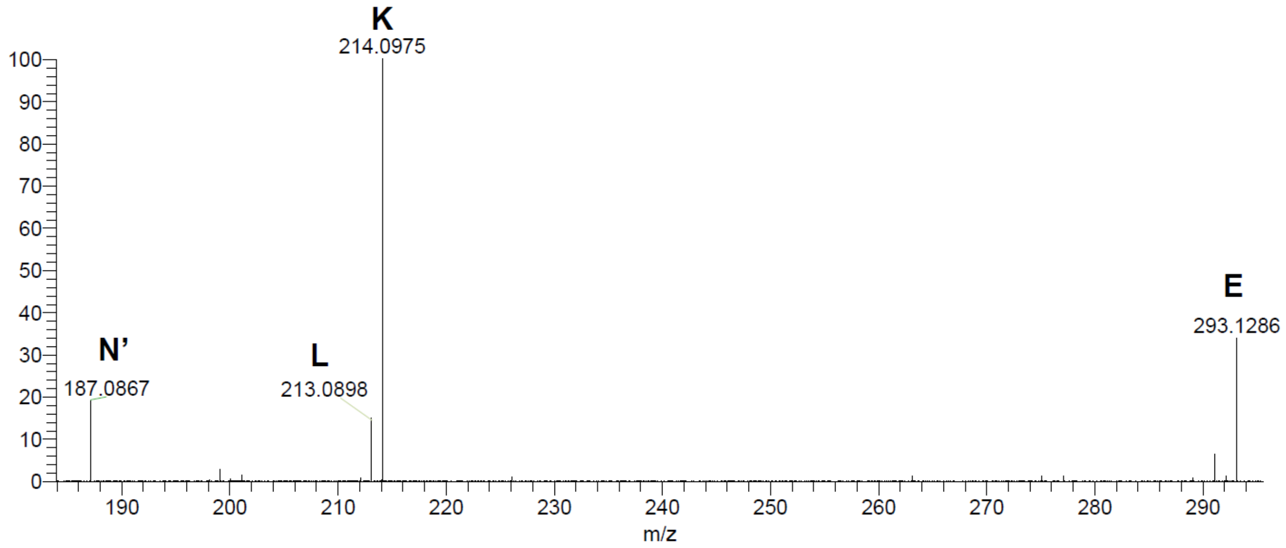

Figure S28. MS[2] spectrum and predicted fragmentation structures of makaluvamine P (**12**).

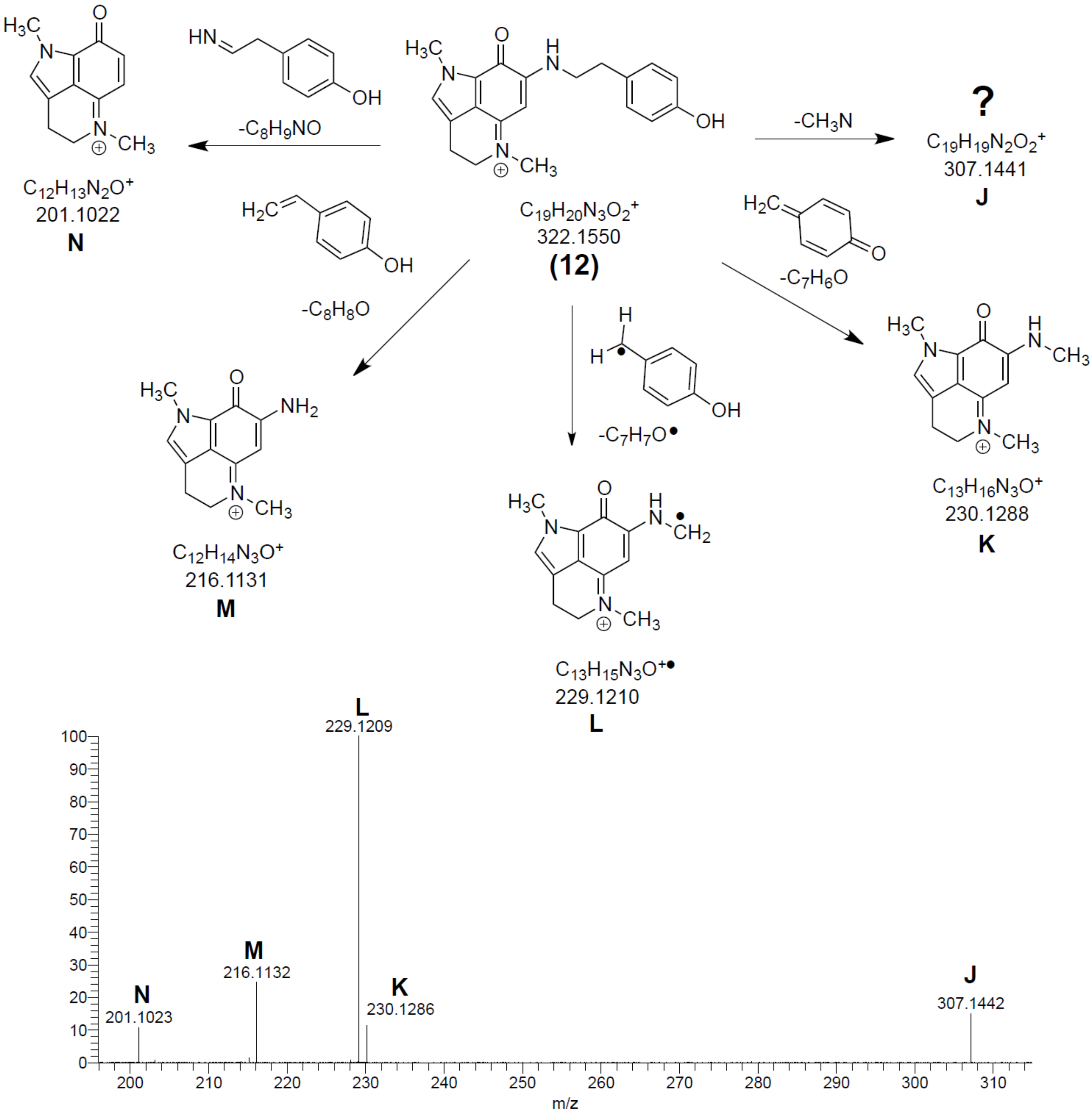

Figure S29. MS^2 spectrum and predicted fragmentation structures of 9-*N*-acetyl makaluvamine A (**13**).

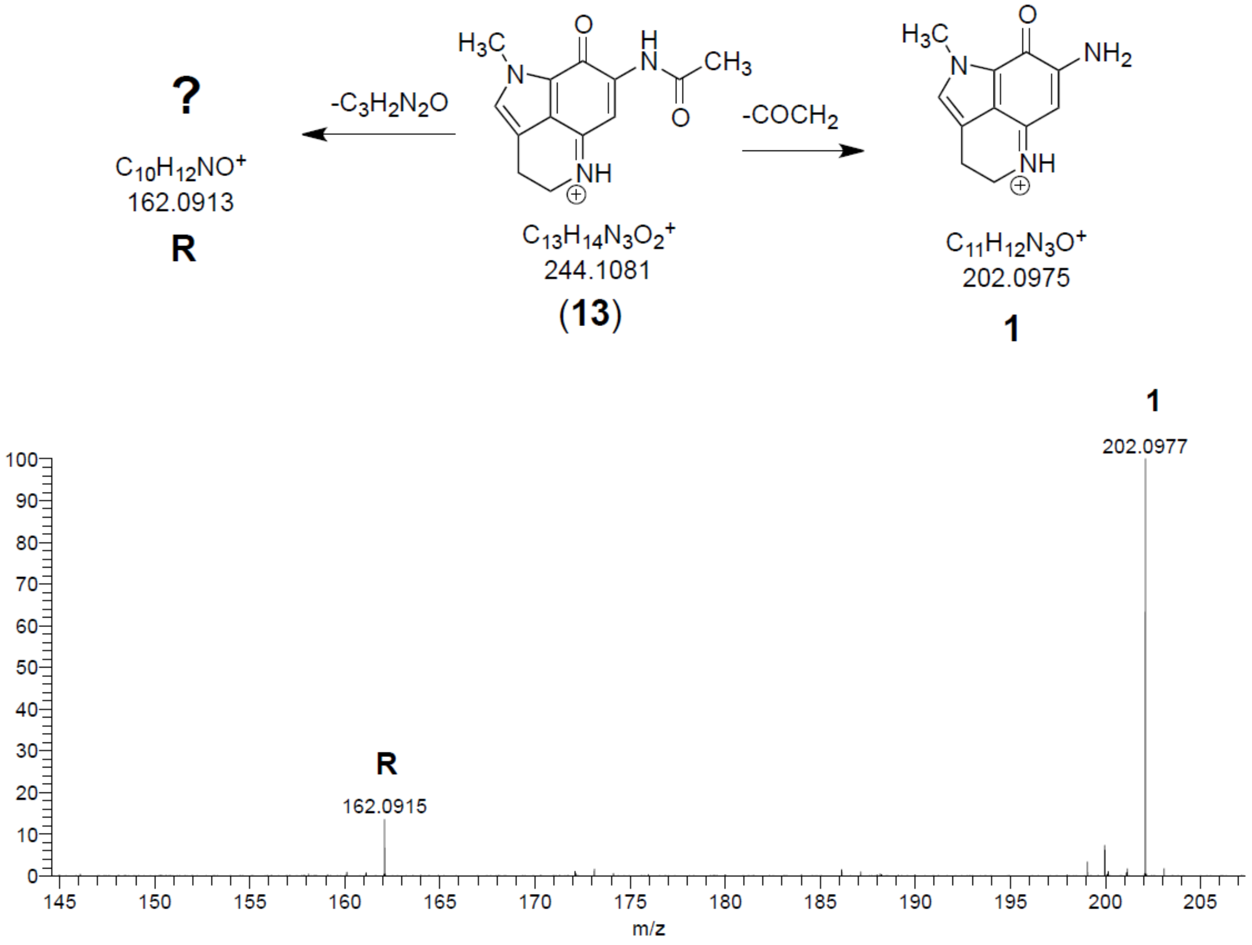

Figure S30. MS² spectrum and predicted fragmentation structures of 9-*N*-acetyl makaluvamine B (**14**).

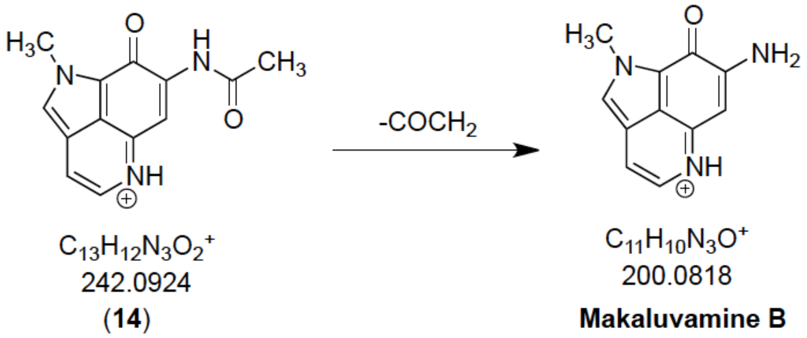

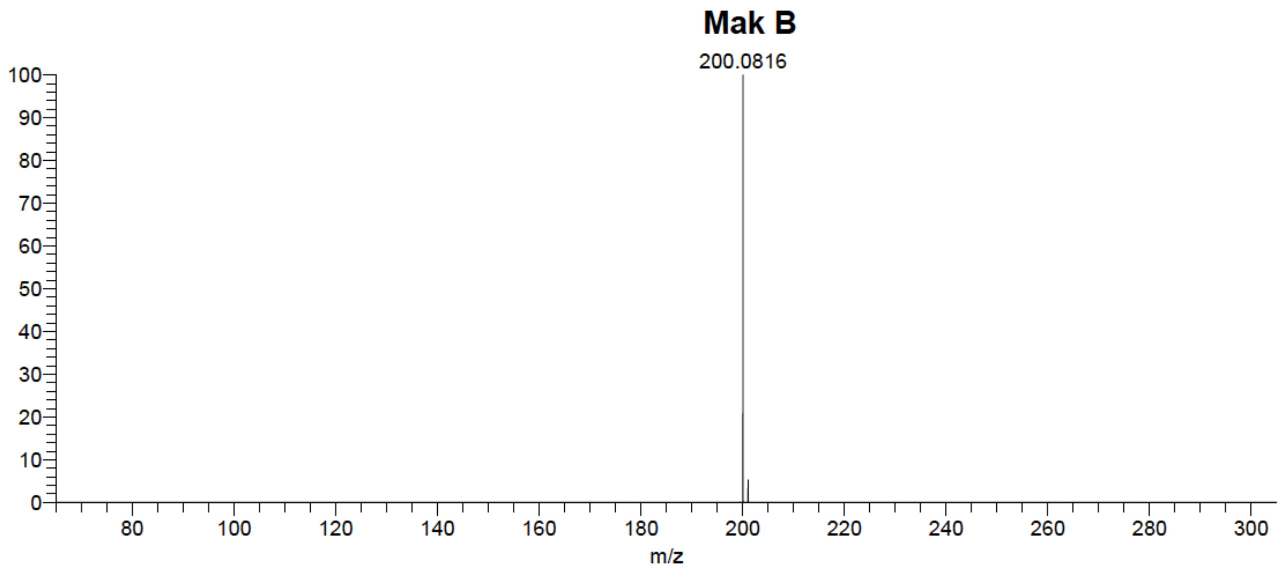

Figure S31. MS[2] spectrum and predicted fragmentation structures of 15-*O*-acetyl makaluvamine J (**15**).

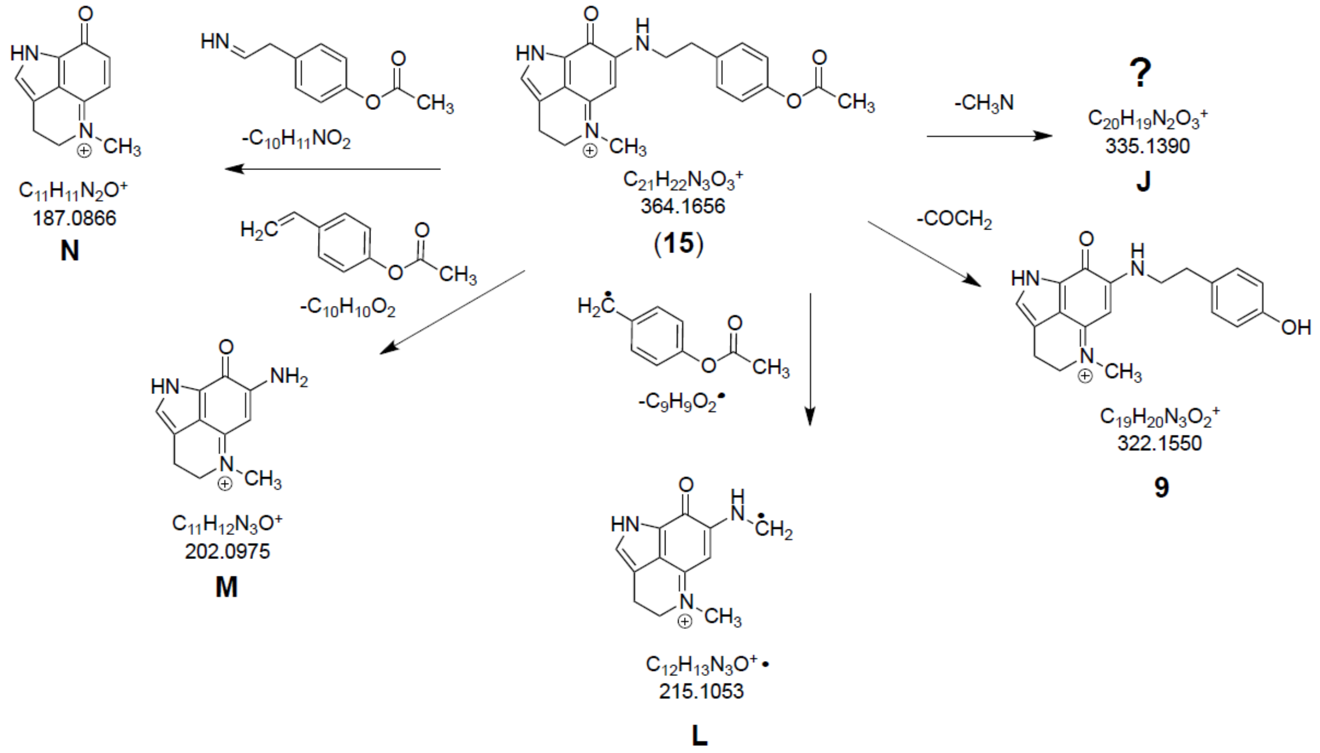

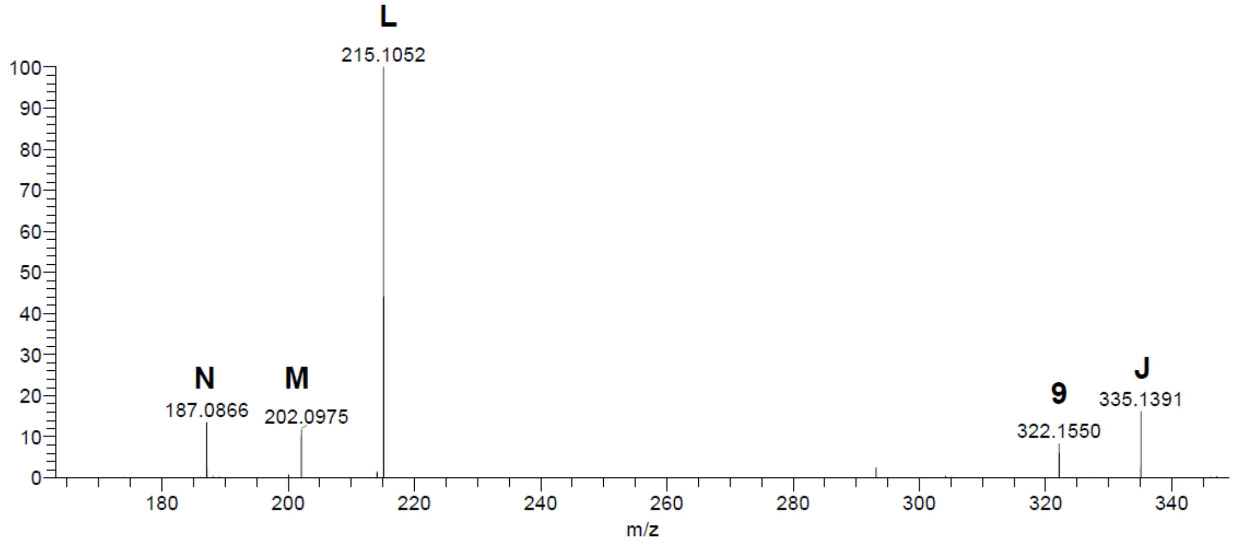

Table S2. HAESI-MS^2 detection of **1–12** in *Z. fuliginosa* extracts.

Sponge ID	Collection Location	Mak A (**1**)	Mak C (**2**)	Dam B (**3**)	Mak H (**4**)	Dam A (**5**)	Dam D (**6**)	Mak D (**7**)	Mak G (**8**)	Mak J (**9**)	Mak K (**10**)	Mak L (**11**)	Mak P (**12**)
02119	PNG		X							X			
03501	PNG		X		X			X		X			
05433	PNG		X	X			X			X			
06132	PNG		X	X				X		X	X		
07339	PNG		X	X		X	X				X		
90104	PNG							X			X		
91164	PNG		X		X	X				X			X
93132	PNG							X			X		
94599	Indonesia		X				X			X			
95000	PNG							X		X			
96183	PNG	X	X										
96500	Indonesia	X		X						X			
97009	Fiji	X	X					X		X	X		
C003369	Unknown		X					X		X			X
C009763	Caroline Islands	X	X						X	X	X	X	X
C010187	PNG		X		X			X	X	X	X		X
C011302	Celebes	X						X			X		
C011303	Celebes	X						X			X		
C012975	Philipine Islands		X										
C016059	Palau							X	X	X	X		X
C016289	Fiji		X					X	X		X		X
C017907	Tonga	X	X					X	X		X		X
C017995	PNG		X					X	X	X	X		X
C018839	PNG	X	X					X	X	X	X		X
C021251	Vanuatu		X										
C021309	Vanuatu		X	X		X			X	X	X	X	X
C022742	PNG		X								X		
C022743	PNG		X	X				X	X	X	X		X

Abbreviation: PNG = Papua New Guinea

Figure S32. MS^2 spectrum and predicted fragmentation structures of 8,15-*O*-diacetyl-8-hydroxy-5a,7,8a-trien-makaluvamine J (**16**).

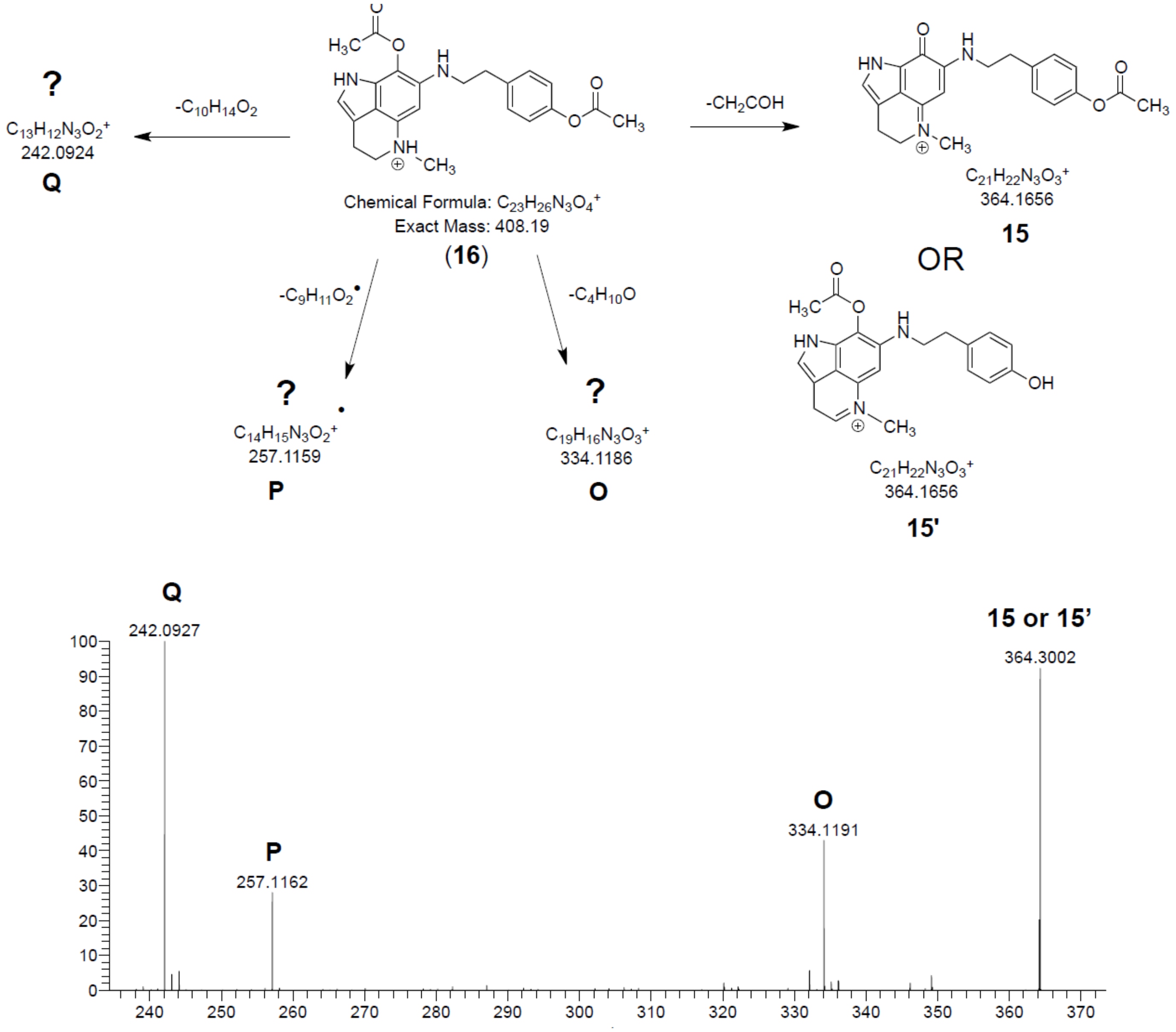

Scheme S6. The acetylation reaction of makaluvamine A (**1**).

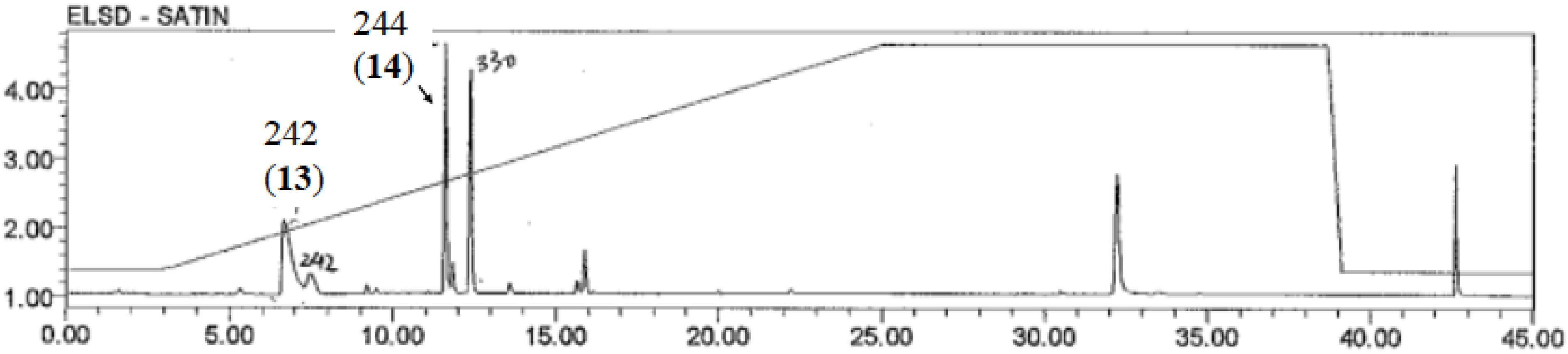

LC-ELSD analysis of the acetate product of makaluvamine A.

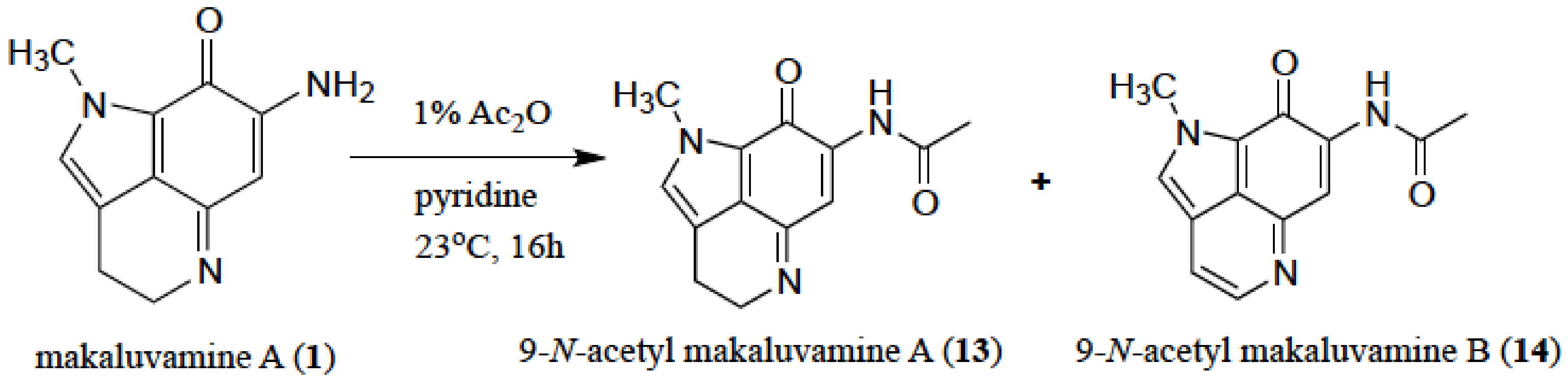

Scheme S7. The acetylation reaction of makaluvamine J (**9**).

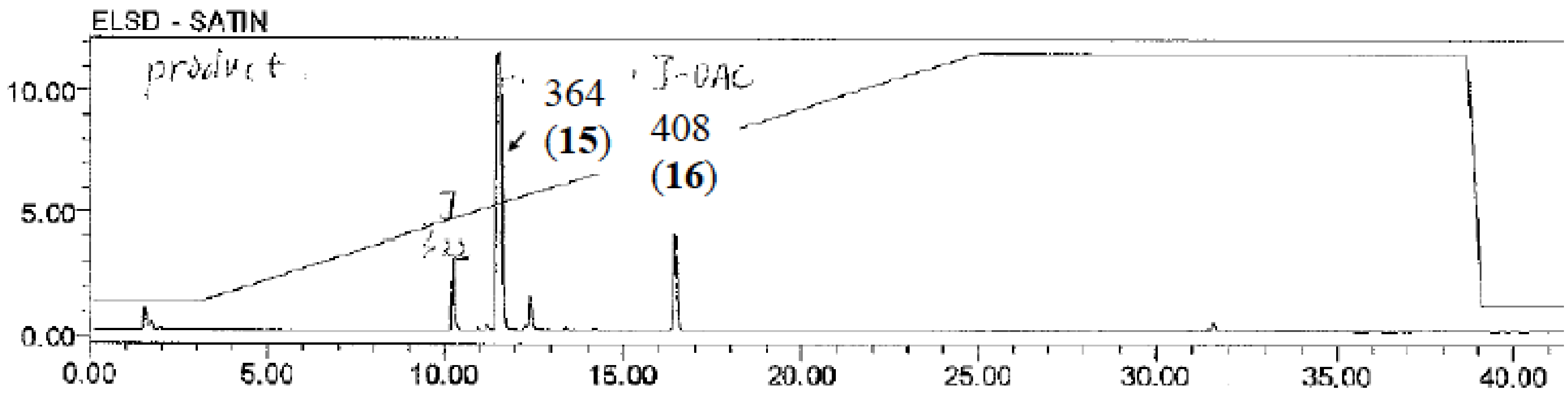

LC-ELSD analysis of the acetate product of makaluvamine J.

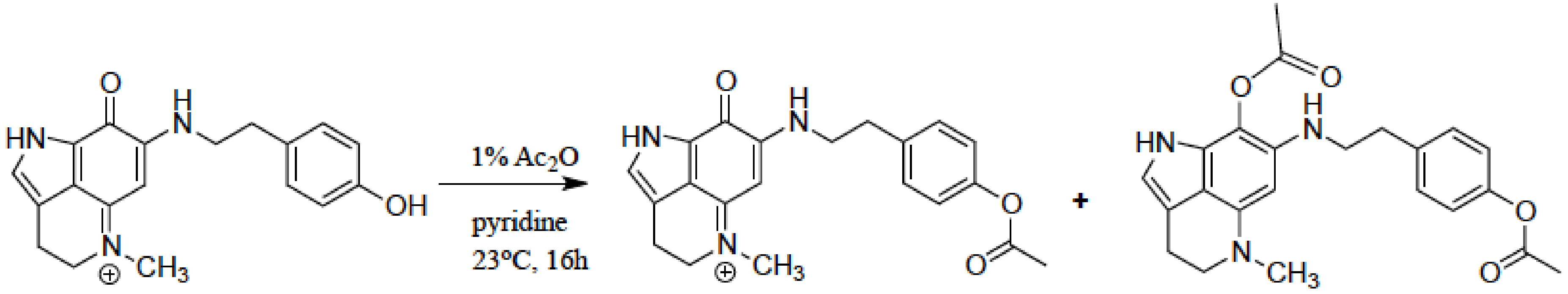

makaluvamine J (**9**)

15-*O*-acetyl makaluvamine J (**15**)

8,15-*O*-diacetyl-8-hydroxy-5a,7,8a-trien-makaluvamine J (**16**)
